# Ascorbic acid-mediated reactive oxygen species homeostasis modulates the switch from tapetal cell division to cell differentiation in Arabidopsis

**DOI:** 10.1093/plcell/koad037

**Published:** 2023-02-14

**Authors:** Si-Yuan Wu, Ling-Li Hou, Jun Zhu, Yi-Chen Wang, Yu-Ling Zheng, Jian-Qiao Hou, Zhong-Nan Yang, Yue Lou

**Affiliations:** Shanghai Key Laboratory of Plant Molecular Sciences, College of Life Sciences, Shanghai Normal University, Shanghai 200234, China; Shanghai Key Laboratory of Plant Molecular Sciences, College of Life Sciences, Shanghai Normal University, Shanghai 200234, China; Shanghai Key Laboratory of Plant Molecular Sciences, College of Life Sciences, Shanghai Normal University, Shanghai 200234, China; Shanghai Key Laboratory of Plant Molecular Sciences, College of Life Sciences, Shanghai Normal University, Shanghai 200234, China; Shanghai Key Laboratory of Plant Molecular Sciences, College of Life Sciences, Shanghai Normal University, Shanghai 200234, China; Shanghai Key Laboratory of Plant Molecular Sciences, College of Life Sciences, Shanghai Normal University, Shanghai 200234, China; Shanghai Key Laboratory of Plant Molecular Sciences, College of Life Sciences, Shanghai Normal University, Shanghai 200234, China; Shanghai Key Laboratory of Plant Molecular Sciences, College of Life Sciences, Shanghai Normal University, Shanghai 200234, China

## Abstract

The major antioxidant L-ascorbic acid (AsA) plays important roles in plant growth, development, and stress responses. However, the importance of AsA concentration and the regulation of AsA metabolism in plant reproduction remain unclear. In Arabidopsis (*Arabidopsis thaliana*) anthers, the tapetum monolayer undergoes cell differentiation to support pollen development. Here, we report that a transcription factor, DEFECTIVE IN TAPETAL DEVELOPMENT AND FUNCTION 1 (TDF1), inhibits tapetal cell division leading to cell differentiation. We identified *SKEWED5*-*SIMILAR 18* (*SKS18*) as a downstream target of TDF1. Enzymatic assays showed that SKS18, annotated as a multicopper oxidase-like protein, has ascorbate oxidase activity, leading to AsA oxidation. We also show that VITAMIN C DEFECTIVE1 (VTC1), an AsA biosynthetic enzyme, is negatively controlled by TDF1 to maintain proper AsA contents. Consistently, either knockout of *SKS18* or *VTC1* overexpression raised AsA concentrations, resulting in extra tapetal cells, while *SKS18* overexpression in *tdf1* or the *vtc1-3 tdf1* double mutant mitigated their defective tapetum. We observed that high AsA concentrations caused lower accumulation of reactive oxygen species (ROS) in tapetal cells. Overexpression of ROS scavenging genes in tapetum restored excess cell divisions. Thus, our findings demonstrate that TDF1-regulated AsA balances cell division and cell differentiation in the tapetum through governing ROS homeostasis.

## Teaser

AsA accumulation mediated by TDF1 controls the transition from cell division to differentiation in the tapetum through governing ROS homeostasis

**IN A NUTSHELL**

**Background:** The tapetum is the innermost layer of the 4 cell-layered anther wall. In Arabidopsis, the tapetum undergoes cell differentiation and transitions into secretory cells. Most nutrients for microspores and/or developing pollen grains are produced by, stored in, and transported from this secretory tapetum. Therefore, the tapetum of flowering plants plays a critical role in mature pollen production. DEFECTIVE IN TAPETAL DEVELOPMENT AND FUNCTION 1 (TDF1) is a tapetum-specific transcriptional factor. In *tdf1* mutants, tapetal cells fail to differentiate after the establishment of the anther wall, leading to complete male sterility.

**Question:** How does TDF1 promote tapetum differentiation?

**Findings:** We found that the loss of TDF1 function induces supernumerary cell divisions and disturbs the subsequent differentiation in the tapetum. We show that the TDF1-SKS18 module regulates the L-ascorbic acid (AsA) oxidation pathway and that the TDF1-VTC1 module regulates AsA biosynthesis. Genetic analyses supported the notion that TDF1 inhibits extra tapetal cell divisions via mediating proper AsA contents. Further studies revealed that AsA-mediated reactive oxygen species (ROS) homeostasis modulates cell divisions in the tapetum. Thus, our findings reveal that TDF1-regulated AsA concentrations contribute to the transition from division to differentiation of tapetal cells through governing ROS homeostasis.

**Next steps:** We will further investigate other pathways or mechanisms that function in tapetal cell division and/or differentiation. Moreover, we will explore whether other SKS members are required for the regulation of tapetum development.

## Introduction

Vitamin C (L-ascorbic acid; AsA), a highly abundant metabolite, has many important roles in growth and metabolism for both plants and animals. In humans, AsA is indispensable for health, since deficiency in AsA causes scurvy (Murad et al. 1981). In plants, AsA serves as a cofactor for many metal-containing enzymes (Smirnoff and Wheeler 2000) and as a required component for cell expansion and cell division (Citterio et al. 1994; Cordoba and Gonzalez-Reyes 1994; Smirnoff 1996; De Tullio et al. 1999). AsA is well known as an antioxidant that detoxifies reactive oxygen species (ROS). Indeed, AsA directly eliminates several types of ROS (Padh 1990). AsA also indirectly removes hydrogen peroxide (H_2_O_2_) via the activity of plant-specific ascorbate peroxidase (APX) in the symplasm (Foyer and Halliwell 1976; Asada and Takahashi 1987). Of note, since AsA is highly enriched in the apoplast (Vanacker et al. 1998), AsA is thought to be the major and likely the only antioxidant buffer in the apoplast (Pignocchi and Foyer 2003).

In plants, the D-mannose/L-galactose pathway is dominant among 4 AsA biosynthetic pathways (Wheeler et al. 1998). In this pathway, D-fructose-6-P is converted to AsA via 8 enzymatic steps (Ishikawa et al. 2018). In the third step, VITAMIN C DEFECTIVE1 (VTC1), a GDP-D-Mannose pyrophosphorylase, catalyzes the rate-limiting step of GDP-D-mannose formation ([Bibr koad037-B15][Bibr koad037-B14]). In Arabidopsis (*Arabidopsis thaliana*), a *vtc1-1* mutant harboring a point mutation in the gene only contains 25% to 30% of wild-type (WT) AsA levels, supporting its critical role in AsA biosynthesis (Conklin et al. 1999). Besides AsA biosynthesis, AsA oxidation and AsA recycling are also incorporated in AsA metabolism. In land plants, ascorbate oxidase (AAO) catalyzes the oxidation of AsA to produce oxidized AsA forms in the apoplast (Mertz 1964; Arrigoni et al. 1981; Pignocchi et al. 2003). Early work in maize (*Zea mays*) roots proposed that AAO oxidizes AsA to maintain quiescent center (QC) cells in a state of reduced mitotic activity (Kerk and Feldman 1995). In parallel, *AAO* mRNA and AAO activity increase in nondividing cells, whereas lower *AAO* expression maintained cell division (Kato and Esaka 1999; Pignocchi et al. 2003), supporting the idea that AAO is involved in mitotic activity. Following AsA oxidation, oxidized AsA is reduced by enzymes in the symplasm to maintain the redox state (Mittler 2002; Foyer and Noctor 2011; Gallie 2013). Thus, the biosynthesis, oxidation, and regeneration of AsA synergistically contribute to the AsA pool, and several regulators have been identified that tightly control AsA metabolism (Zhang et al. 2009; Yu et al. 2019; Broad et al. 2020; Chen et al. 2020; Liu et al. 2022; Ma et al. 2022). However, the regulatory mechanisms behind AsA homeostasis in plant reproduction have remained largely unexplored.

In plant reproduction, anther development is critical to the generation of male gametophytes (pollen grains). The tapetum, the innermost layer of a 4-layered anther wall, acts as a nursing tissue that provides nutrients and materials to microspores and/or developing pollen grains (Pacini et al. 1985). In Arabidopsis, tapetal cells undergo cell fate establishment, cell differentiation, and programmed cell death (PCD) (Sanders et al. 1999; Quilichini et al. 2014). Several studies have shown that multiple signaling pathways determine and maintain the fate of tapetal cells (Feng and Dickinson 2010; Huang et al. 2016; Li et al. 2017b; Cui et al. 2018; Chen et al. 2019). Following the formation of the tapetal monolayer, several morphological features indicate that tapetal cells start differentiating into a secretory tissue. Tapetal differentiation is exemplified by a dense cytoplasm, vacuolization, cell shrinkage, endoreduplication onset, and cell wall dissolution (Pacini 1990; Cecchetti et al. 2015; Huang et al. 2017; Jacobowitz et al. 2019; Valuchova et al. 2020), which allows for an increase in active metabolism and the polarized secretion of an array of metabolites for microspore growth (Dobritsa et al. 2010; Xu et al. 2010; Grienenberger et al. 2011; Cui et al. 2016; Wang et al. 2017; Goodman et al. 2021). Later, tapetal cells enter PCD and release their stored components for pollen wall formation (Wu and Cheung 2000; Huang et al. 2013; Xiong et al. 2016; Wang et al. 2018). Timely PCD of the tapetum is crucial for pollen development, which is tightly controlled by a sophisticated transcriptional regulatory network (Li et al. 2006; Vizcay-Barrena and Wilson 2006; Phan et al. 2011; Niu et al. 2013). Although significant progress has been made towards understanding tapetal development, the molecular mechanism underlying tapetal differentiation is not clear.

In Arabidopsis, several tapetum-expressed transcriptional factor (TF) genes cooperatively form a tapetal regulatory pathway (DYSFUNCTIONAL TAPETUM1 [DYT1]-TDF1- ABORTED MICROSPORES [AMS]- MALE STERILE188 [MS188]-MALE STERILITY1[MS1]) to control tapetum development and function (Wilson et al. 2001; Sorensen et al. 2003; Zhang et al. 2006, 2007; Ito et al. 2007; Yang et al. 2007; Zhu et al. 2011; Gu et al. 2014; Lou et al. 2014, 2018; Lu et al. 2020). Among these TFs, DEFECTIVE IN TAPETAL DEVELOPMENT AND FUNCTION 1 (TDF1) is an R2R3 MYB whose transcriptional cascades support tapetum pollen wall formation (Lou et al. 2018). A previous study showed that the tapetal cells in the *tdf1* mutant have a swollen shape with additional vacuolization and fail to transition to secretory tissue after the anther wall was established (Zhu et al. 2008). However, how TDF1 promotes tapetum differentiation remains unknown.

Here, we show that TDF1 is a negative regulator of AsA accumulation in anthers. TDF1 directly activates the expression of *SKU5 SIMILAR 18* (*SKS18*) encoding a multicopper oxidase (MCO)-like protein functioning in AsA oxidation with a copper cofactor. Additionally, TDF1 regulates AsA biosynthesis via negatively controlling the accumulation of VTC1. Our physiological, biochemical, and genetic evidence demonstrated that TDF1 prevents the supernumerary division of tapetal cells and enables tapetal differentiation via mediating a proper AsA level. We further showed that a high level of AsA leads to low ROS levels in the tapetum. Consistently, increasing the expression of genes encoding ROS scavenging enzymes restored excess tapetal cell divisions seen in WT anthers. Conversely, a low level of AsA allowed ROS to accumulate and repress tapetal cell divisions. Together, our findings reveal that TDF1-regulated AsA concentration contributes to the transition from division to differentiation of tapetal cells through governing ROS homeostasis.

## Results

### TDF1 regulates the transition from cell division to differentiation in tapetum

**Figure 1:**
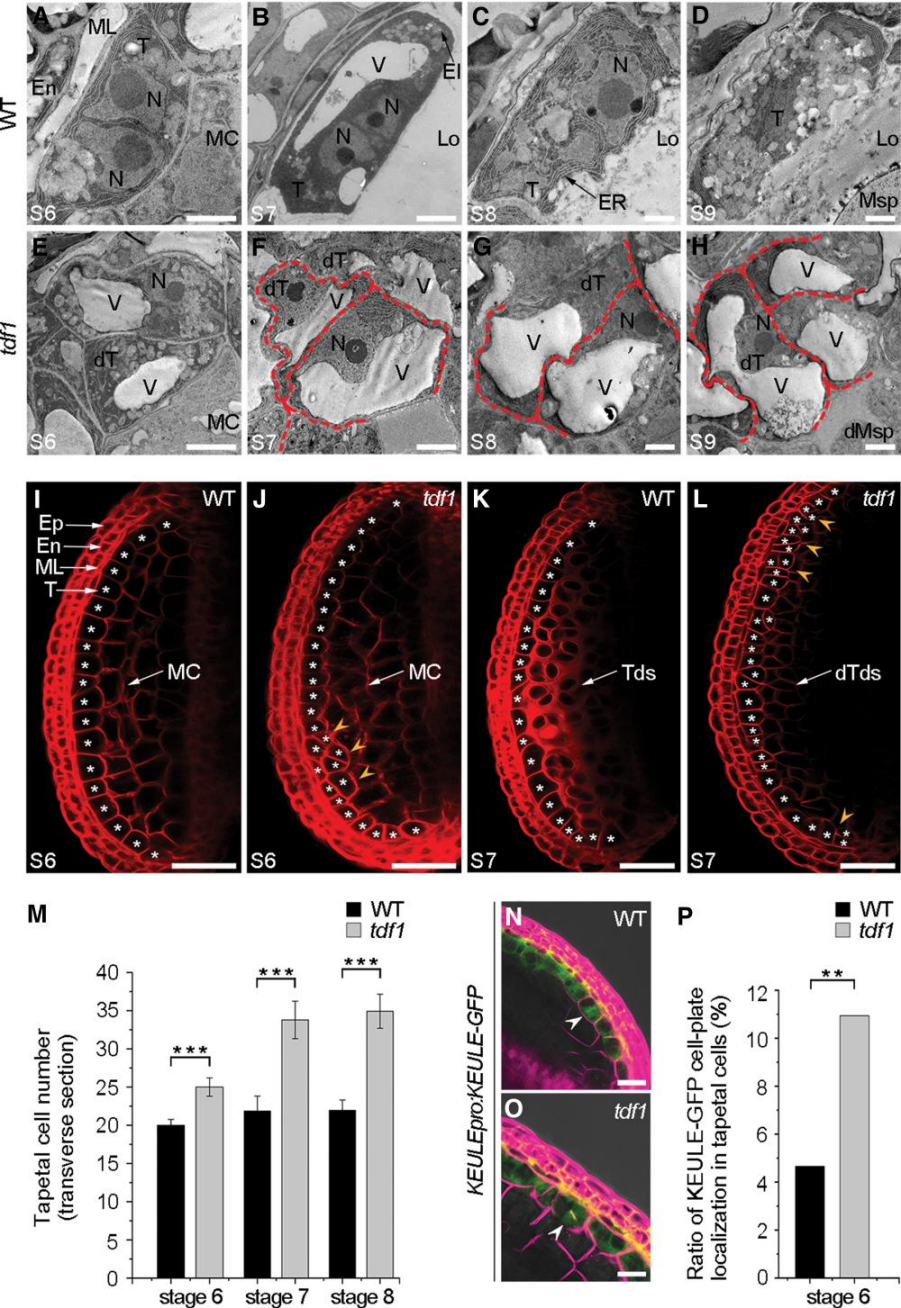
TDF1 regulates the transition from cell division to differentiation in tapetum. TEM images of secretory tapetum development in WT (A–D) and *tdf1* (E–H) from Stages 6 to 9. A–D) In WT, tapetal cells initiate the differentiation with 2 nuclei and vacuolization, later undergoing cell wall dissolution and a dense cytoplasm to support their active metabolisms. E–H) In *tdf1*, irregular tapetal cells are hypertrophic with more vacuoles and their boundaries are jigsaw-shaped (red dotted lines). Scale bars, 5 μm. I–L) One abaxial locule in anther is stained with FM4-64 in WT and *tdf1* at Stages 6 to 7. I and K) WT anther layers comprise 4 somatic monolayers: epidermis, endothecium, middle layer, and tapetum (asterisks). J and L) The multilayered tapetum (yellow arrowheads) is present in *tdf1*. Scale bars, 50 μm. M) Tapetal cell number based on the FM4-64 staining in WT and *tdf1* from Stages 6 to 8. Data are means ± SD. *n* = 30 anthers. ***P < 0.001 (*t*-test). N and O) Expression of *KEULEpro:KEULE-GFP* with FM4-64 in WT (N) and *tdf1* (O) anthers. Scale bars, 20 μm. White arrowheads indicate the cell-plate localization of KEULE-GFP. P) Rate of cell-plate localization of KEULE-GFP in WT and *tdf1* tapetal cells. *n* = 386 (WT tapetal cells), *n* = 329 (*tdf1* tapetal cells). **P < 0.01 (*z*-test). Ep, epidermis; En, endothecium; EI, elaioplast; ER, endoplasmic reticulum; Lo, locule; ML, middle layer; MC, meiocytes; Msp, microspore; *N*, nucleus; T, tapetum; Tds, tetrads; V, vacuole; dT, defective tapetum; dTds, defective tetrads; dMsp, defective microspore.

The *tdf1* mutant is defective in early tapetum development in Arabidopsis (Zhu et al. 2008). To characterize the developmental progression of the secretory tapetum, we examined anthers from WT and *tdf1* at Stages 6 to 9 by transmission electron microscopy (TEM). At Stage 6, the WT tapetal cells were binucleate and cytoplasmically condensed (Fig. 1A). At Stage 7, the cytoplasm of tapetal cells contained a large intracellular vacuole and the boundaries of tapetal cells were well defined in a spongy shape (Fig. 1B). At later stages, the tapetal cells were metabolically active with endoplasmic reticulum (ER), elaioplasts, and cytoplasmic lipid bodies, and their locule-facing edges became wavy to release more materials required for pollen growth following cell wall degradation (Fig. 1, C and D). These cytological characteristics are typical of the secretory tapetum in WT anthers. By contrast, some irregular tapetal cells appeared in *tdf1* initially at Stage 6 (Fig. 1E). In subsequent stages, the tapetal cells of the mutant were hypervacuolated and swollen with jigsaw-shaped boundaries (Fig. 1, F–H). Moreover, tapetal cells of *tdf1* had few organelles (Fig. 1, F–H). These observations suggest that the tapetum fails to differentiate into secretory cells in *tdf1* anthers.

Arabidopsis anthers have 2 large abaxial locules and 2 small adaxial locules (Scott et al. 2004). Since the 2 pairs of locules are symmetric, we stained somatic cell membranes in 1 abaxial locule with the dye FM4-64 to test whether abnormal cell proliferations occurred in *tdf1* tapetum. In WT anther locules, tapetal cells formed a highly ordered single layer of surrounding meiocytes (Fig. 1I). Following the end of meiosis, tapetal cells proliferate by anticlinal divisions within the monolayer (Fig. 1K and Supplemental Fig. S1A). However, in *tdf1* anther locules, we observed a multilayered tapetum at Stages 6 to 8 (Fig. 1, J and L and Supplemental Fig. S1B). We counted the number of tapetal cells based on the observations of FM4-64 staining. In the *tdf1* mutant, the number of tapetal cells was significantly higher than that of the WT (Fig. 1M). To determine if the over-proliferation in the tapetum was caused by excess cell divisions, we introduced the cell-plate-associated marker KEULE-GFP (green fluorescent protein), which characterizes the emergence of the cell plate from mitosis to cytokinesis, into WT and *tdf1* plants (Steiner et al. 2016). In agreement with previous findings, in these *KEULEpro:KEULE-GFP* transgenic plants, KEULE-GFP showed a cytosolic localization in nondividing cells (Fig. 1N and Supplemental Fig. S2) and had a localized cell plate throughout cytokinesis (Fig. 1, N and O, arrowheads). A quantification of the number of tapetal cells showing cell-plate localization relative to the total number of tapetal cells in these transgenic plants revealed that the ratio of cell-plate localization in *tdf1* tapetal cells is higher than that of the WT (Fig. 1P). Moreover, KEULE-GFP signals presented a misoriented cell-plate localization in *tdf1* tapetal cells (Fig. 1O), which coincided with the disordered tapetal cell division seen in *tdf1* (Fig. 1, J and M). Together, these findings indicate that TDF1 inhibits tapetal cell divisions and promotes the monolayer tapetum to differentiate into the secretory tissue.

### SKS18, encoded by a candidate target of TDF1, locates to the apoplast and cell wall of tapetal cells

**Figure 2:**
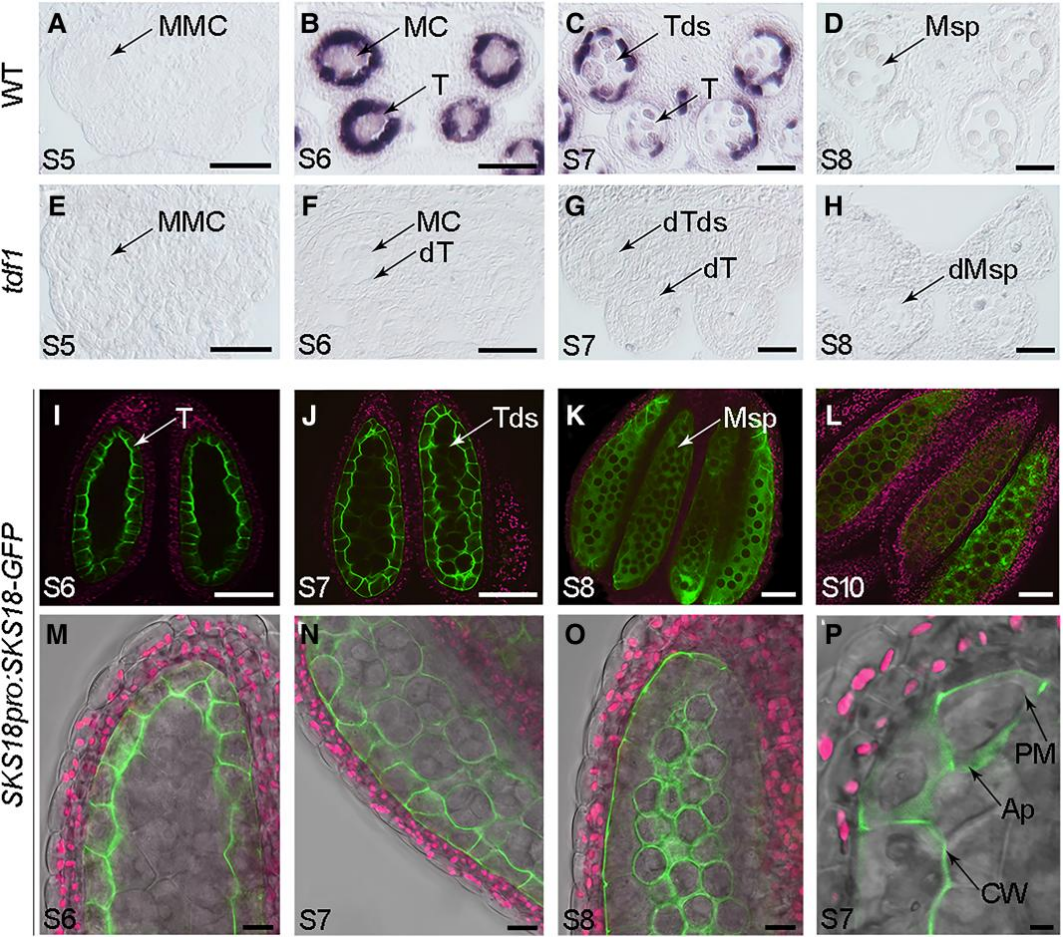
SKS18 locates to the apoplast and cell wall of tapetal cells. RNA in situ hybridization of *SKS18* transcripts in anthers of WT (A–D) and *tdf1* (E–H) using an antisense probe. Scale bars, 20 μm. I–L) Localization of SKS18-GFP from the *SKS18pro:SKS18-GFP* transgene at Stages 6 to 10. M–O) Higher magnification view of SKS18-GFP location in anthers. Chloroplast autofluorescence is shown in magenta. P) SKS18-GFP fluorescence is restricted to the apoplast and cell wall of tapetal cells after treatment with 100 mM mannitol. Scale bars, 20 μm. Ap, apoplast; CW, cell wall; PM, plasma membrane; MMC, mother microspore cell; MC, meiocytes; Msp, microspore; T, tapetum; Tds, tetrads; dT, defective tapetum; dTds, defective tetrads; dMsp, defective microspore.

Our previous microarray data identified 828 candidate genes targeted by TDF1. A MapMan analysis showed that 6 members (*SKS10*, *SKS11*, *SKS12*, *SKS13*, *SKS14*, *SKS18*) of the *SKS* gene family are categorized into the group of miscellaneous enzymes; this group is the most enriched group in the functional classification of these candidates (Lou et al. 2018). In this study, we confirmed that *SKS18* is specifically expressed in WT inflorescence by RT-PCR (Supplemental Fig. S3A); importantly, its expression level was greatly decreased in *tdf1* inflorescence (Supplemental Fig. S3C). To determine the spatiotemporal patterns of *SKS18* in anthers, we conducted mRNA in situ hybridization with an *SKS18* probe in anthers from WT and *tdf1* plants. In the WT, we detected little hybridization signal at Stage 5 (Fig. 2A). We detected *SKS18* signal throughout Stages 6 to 7, with a strong signal in the tapetum and a weak signal in meiocytes and tetrads (Fig. 2, B and C). The signal was much lower after the release of microspores (Fig. 2D and Supplemental Fig. S4A). However, we detected no hybridization signal in *tdf1* anthers (Fig. 2, E–H and Supplemental Fig. S4B). These results show that *SKS18* is preferentially expressed in tapetum during the differentiation stage, and its expression is abolished by the dysfunction of *TDF1*.

To monitor SKS18 localization, we introduced the construct *SKS18pro:SKS18-GFP* into WT plants. In these *SKS18pro:SKS18-GFP* transgenic plants, we initially observed SKS18-GFP fluorescence at the periphery of tapetal cells during meiosis, followed by a gradual accumulation in locules following the secretion of tapetum (Fig. 2, I–L). A close examination indicated that SKS18-GFP predominantly localizes to the inner tangential walls and the radial walls of tapetal cells (Fig. 2M). Following meiosis, SKS18-GFP fluorescence surrounded both tapetal cells and tetrads (Fig. 2N). Later, only a small amount of fluorescence localized to the outer tangential walls of tapetal cells, with the remainder filling in the locule to encase microspores (Fig. 2O). Given this peripherally localized fluorescence, we wondered whether SKS18-GFP was associated with the tapetal plasma membrane or cell wall. We thus treated anthers with 100 mM mannitol to plasmolyze tapetal cells, finding that the fluorescence in tapetal cells localized to the apoplast and cell wall rather than the plasma membrane (Fig. 2P). This protein localization data suggest that SKS18 may play a role in tapetum development and/or function.

### *SKS18* is a direct transcriptional target of TDF1

Combined with the expression patterns of *SKS18* in WT and *tdf1* anthers (Fig. 2, A–H), we hypothesized that *SKS18* is likely a target of TDF1. A previous study demonstrated that *ABORTED MICROSPORE* (*AMS*) was a direct target of TDF1 (Lou et al. 2018). To investigate whether *SKS18* is downregulated in *tdf1*, we measured the expression patterns of *SKS18* in *ams* anthers by mRNA in situ hybridization. We observed a hybridization signal for *SKS18* specifically at Stage 6 and not at stages (Supplemental Fig. S4, C–F), indicating that *SKS18* transcript levels are not largely affected at the early stage but are suppressed at later stages due to the abnormal tapetum degeneration of *ams*. Additionally, in *ams gSKS18pro:SKS18-GFP* transgenic plants harboring the genomic copy of SKS18, including the promoter, cloned in-frame and upstream of *GFP*, some SKS18-GFP fluorescence was still evident in tapetal cells (Supplemental Fig. S4H). However, we observed no protein fluorescence in *tdf1 gSKS18pro:SKS18-GFP* transgenic plants (Supplemental Fig. S4G). These results indicate that the expression of *SKS18* is specifically repressed by the mutation in *TDF1* rather than *AMS*.

**Figure 3:**
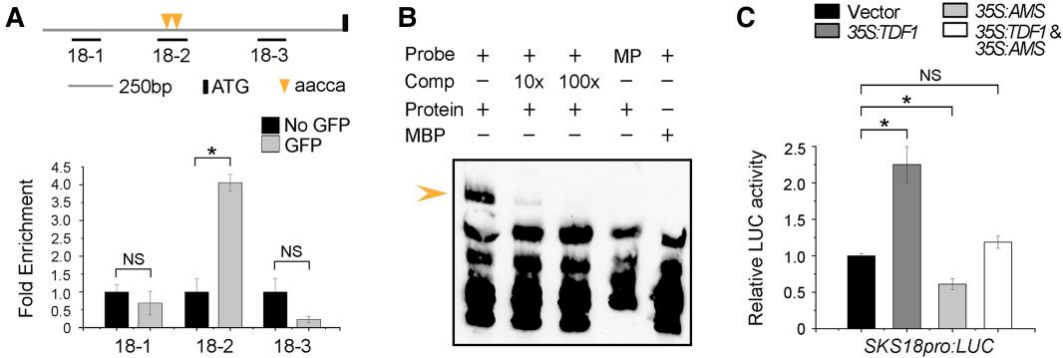
*SKS18* is a direct target of TDF1. A) Potential MYB-binding sites in the *SKS18* promoter region. Triangles indicate the MYB-binding sites. ChIP-qPCR assay shows TDF1 binding to the *SKS18* promoter region in the *tdf1 gTDF1pro:TDF1-GFP* complemented lines. Fold-enrichment calculations from 2 replicate qPCR assays in 3 independent ChIP experiments. Data are means ± SD. *P < 0.05; NS, not significant (*t*-test). B) EMSA with recombinant MBP-TDF1, biotin-labeled probes, mutated probes (MP), 10-fold and 100-fold excess of unlabeled competitor probes. MBP was used as a negative control. The arrowhead indicates a shift band. Three biological repeats were performed, with similar results. C) Transient dual-luciferase assay of TDF1 and AMS transactivating the *SKS18* promoter in Arabidopsis protoplasts. Data are means ± SD of 3 biological replicates. *P < 0.05; NS, not significant (*t*-test).

To identify whether TDF1 directly controlled *SKS18* expression, we performed a chromatin immunoprecipitation (ChIP) assay using inflorescences from *tdf1 gTDF1pro:TDF1-GFP* transgenic plants. TDF1 was reported to bind to the sequence AACC(T/A/C) (Lou et al. 2018). We scanned for this sequence and identified 2 such motifs (−970 to −965 bp and −850 to −845 bp relative to the ATG) in the *SKS18* promoter fragment used for the genetic complementation above. We thus designed primers to amplify these motifs to measure their enrichment in ChIP samples (Fig. 3A). Quantitative ChIP-PCR (ChIP-qPCR) showed that the 18-2 fragment containing the 2 copies of the motif is particularly enriched compared with the mock control (without the anti-GFP monoclonal antibody) (Fig. 3A). We also performed an electrophoretic mobility shift assay (EMSA) to test whether TDF1 binds to this site in the *SKS18* promoter. We generated biotin-labeled probes containing the 18-2 fragment and purified recombinant maltose-binding protein (MBP)-TDF1 in *Escherichia coli*. We determined that MBP-TDF1 can indeed bind to the biotin-labeled probe. Importantly, unlabeled competitor probes or labeled GGGG-containing mutated probes incubated with MBP-TDF1 abolished this shift, confirming the specificity of the binding (Fig. 3B). These results show that TDF1 directly binds the AACCA regions in the *SKS18* promoter.

To investigate the effect of TDF1 on *SKS18* activation, we carried out transient dual-luciferase (LUC) assays in Arabidopsis protoplasts. Since *TDF1* and *SKS18* were co-expressed in anthers (Fig. 2, I–L and Supplemental Fig. S5), we co-transformed the constructs for the reporter (*SKS18pro:LUC*) and effector (*35S:TDF1*) into protoplasts. The empty vector was included as a control. Co-infiltration of *35S:TDF1* and *SKS18pro:LUC* in *Nicotiana benthamiana* leaves resulted in strong LUC intensity, demonstrating that TDF1 activates *SKS18* transcription (Fig. 3C). Previous studies revealed that the TDF1-AMS complex facilitated the expression of some tapetum-specific genes in an additive manner (Lou et al. 2018). Thus, we co-transformed the construct *35S:AMS*, another effector, with the same reporter: the resulting LUC activity was low. Moreover, when both effectors were co-expressed with the *SKS18pro:LUC* reporter, LUC activity did not significantly increase. These results indicate that the TDF1-AMS complex has no significant effect on activating *SKS18* expression. Taken together, the above results demonstrate that *SKS18* is a direct and specific transcriptional target of TDF1.

### The TDF1-SKS18 module functions in the transition from tapetal cell division to differentiation

**Figure 4:**
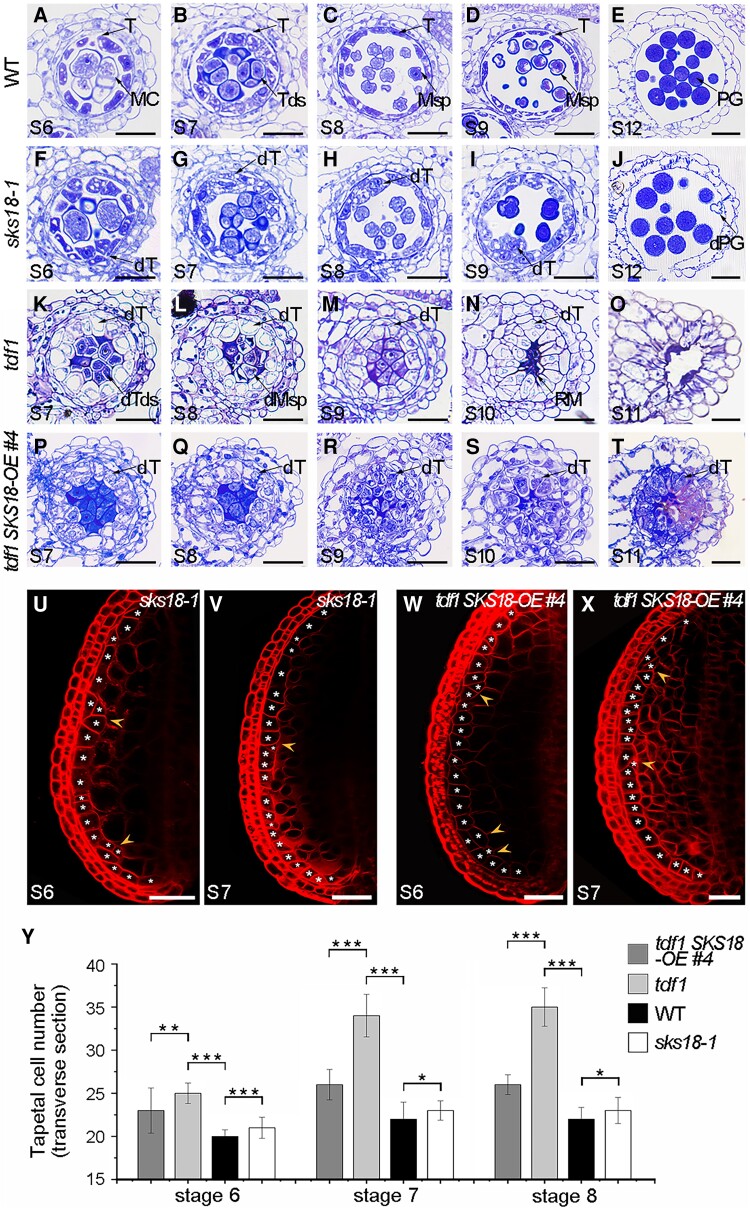
The TDF1-SKS18 module functions in transition from tapetal cell division to differentiation. Semi-thin sections of anthers from WT (A–E), *sks18-1* (F–J), *tdf1* (K–O), and *tdf1 SKS18-OE #4* plants (P–T) at the indicated stages. Scale bars, 20 μm. One abaxial locule in anther was stained with FM4-64 and extra tapetal cells (arrowheads) are present in *sks18-1* (U and V) and *tdf1 SKS18-OE #4* (W and X) plants. Scale bars, 50 μm. Y) Tapetal cell number based on the FM4-64 staining in WT, *tdf1*, *sks18-1*, and *tdf1 SKS18-OE #4* plants from Stages 6 to 8. Data are means ± SD. *n* = 30 anthers. *P < 0.05; **P < 0.01; ***P < 0.001 (*t*-test). MC, meiocytes; Msp, microspore; RM, remnants; T, tapetum; Tds, tetrads; PG, pollen grain; dT, defective tapetum; dTds, defective tetrads; dMsp, defective microspore; dPG, degenerated pollen grain.

To assess the role of SKS18 in tapetum development, we obtained a T-DNA insertion line with an insertion in the *SKS18* gene (WiscDsLoxHs032_08H) (Supplemental Fig. S3B). We confirmed the drastically lower transcript levels of *SKS18* in this mutant by RT-qPCR (Supplemental Fig. S3C); this mutant exhibited normal vegetative growth with a few aborted pollen grains (Supplemental Fig. S6A). We performed a histological analysis of tapetal cells from the WT and *sks18-1*. Compared with the WT, a discontinuous multilayered tapetum appeared in *sks18-1* at Stage 6 (Fig. 4, A and F). From Stages 7 to 9, tapetal cells in WT underwent vacuolization and took on a spongy appearance for their secretory activities (Fig. 4, B–D), whereas we observed expanded tapetal cells with cytoplasm loosening in *sks18-1* (Fig. 4, G–I). Nevertheless, tapetal degradation was similar in *sks18-1* and WT at Stage 12 (Fig. 4, E and J). We also targeted the *SKS18* locus by clustered regularly interspaced short palindromic repeat (CRISPR)/CRISPR-associated nuclease 9 (Cas9)-mediated gene editing (Supplemental Fig. S3B) and named this allele *sks18-2*. The *sks18-2* mutant exhibited a morphology defect similar to that of *sks18-1* (Supplemental Fig. S6, B–E), revealing that the knockout of *SKS18* leads to abnormal tapetum development. Genetic complementation also confirmed that the *sks18* mutant phenotype was attributable to the loss of SKS18 function (Supplemental Fig. S6, F–H). To explore whether the mutation of *SKS18* caused anomalous tapetum proliferation, we stained *sks18-1* locules with FM4-64 and counted the number of tapetal cells. These cytological observations indicated that tapetal cells of *sks18-1* show a similar but weaker phenotype than *tdf1* (Fig. 4, U, V, and Y and Supplemental, Fig. S1D), indicating that the *sks18* mutant partially phenocopies *tdf1* anthers.

We asked whether increasing the expression of *SKS18* might restore the tapetum defects seen in *tdf1*. To this end, we constructed an *SKS18* overexpression vector using the strong tapetum promoter from *DYT1550* (Gu et al. 2014) and introduced the resulting construct into *tdf1*/*TDF1* plants. We observed complete male sterility in all 21 *tdf1 gDYT1550pro:SKS18-GFP* (named *tdf1 SKS18-OE*) transgenic lines in the T1 generation. We chose several independent lines to measure their *SKS18* expression levels by RT-qPCR: *SKS18* transcript levels in these transgenic plants were at least 6 times higher than in *tdf1* plants (Supplemental Fig. S7A). We selected *tdf1 SKS18-OE #4* showing the highest *SKS18* expression for phenotypic characterization. Consistently, its SKS18-GFP protein location was similar to that seen in *SKS18pro:SKS18-GFP* transgenic plants (Supplemental Fig. S7, J–L). FM4-64 staining showed that although the tapetal cell number in *tdf1 SKS18-OE #4* anther is still higher than in WT, it was significantly lower compared to that in *tdf1* (Fig. 4, W–Y and Supplemental Fig. S1C), suggesting that overexpression of *SKS18* partially mitigates the supernumerary division of tapetum in the *tdf1* mutant.

Interestingly, Alexander's staining showed that pollen remnants stain green in *tdf1* anthers, whereas the same remnants stained purple in anthers from *tdf1 SKS18-OE* lines, suggesting that the cytoplasm of pollen was not fully degenerated (Supplemental Fig. S7, B–D). Cytological observations showed that the swollen tapetal cells in *tdf1 SKS18-OE* lines have a condensed cytoplasm and remain in the locule (Fig. 4, P–T and Supplemental Fig. S7, F–I), while the hypervacuolated tapetal cells in *tdf1* had degenerated at Stage 11 (Fig. 4, K–O). We used diethyloxadicarbocyanine iodide (DiOC_2_) to stain fatty acids in the tapetum. Like in WT, we observed a fluorescent signal in the tapetum from *tdf1 SKS18-OE #4* anthers (Supplemental Fig. S8, A–C and G–I), but not in the tapetum from *tdf1* anthers at Stages 7 to 11 (Supplemental Fig. S8, D–F). These results show that the defective tapetal cells in transgenic plants have partially restored function to synthesize some materials for microspore growth, suggesting that the TDF1-SKS18 module controls a division and/or differentiation switch in the tapetum that may benefit subsequent tapetum function.

### SKS18 catalyzes AsA oxidation with copper cofactor

**Figure 5:**
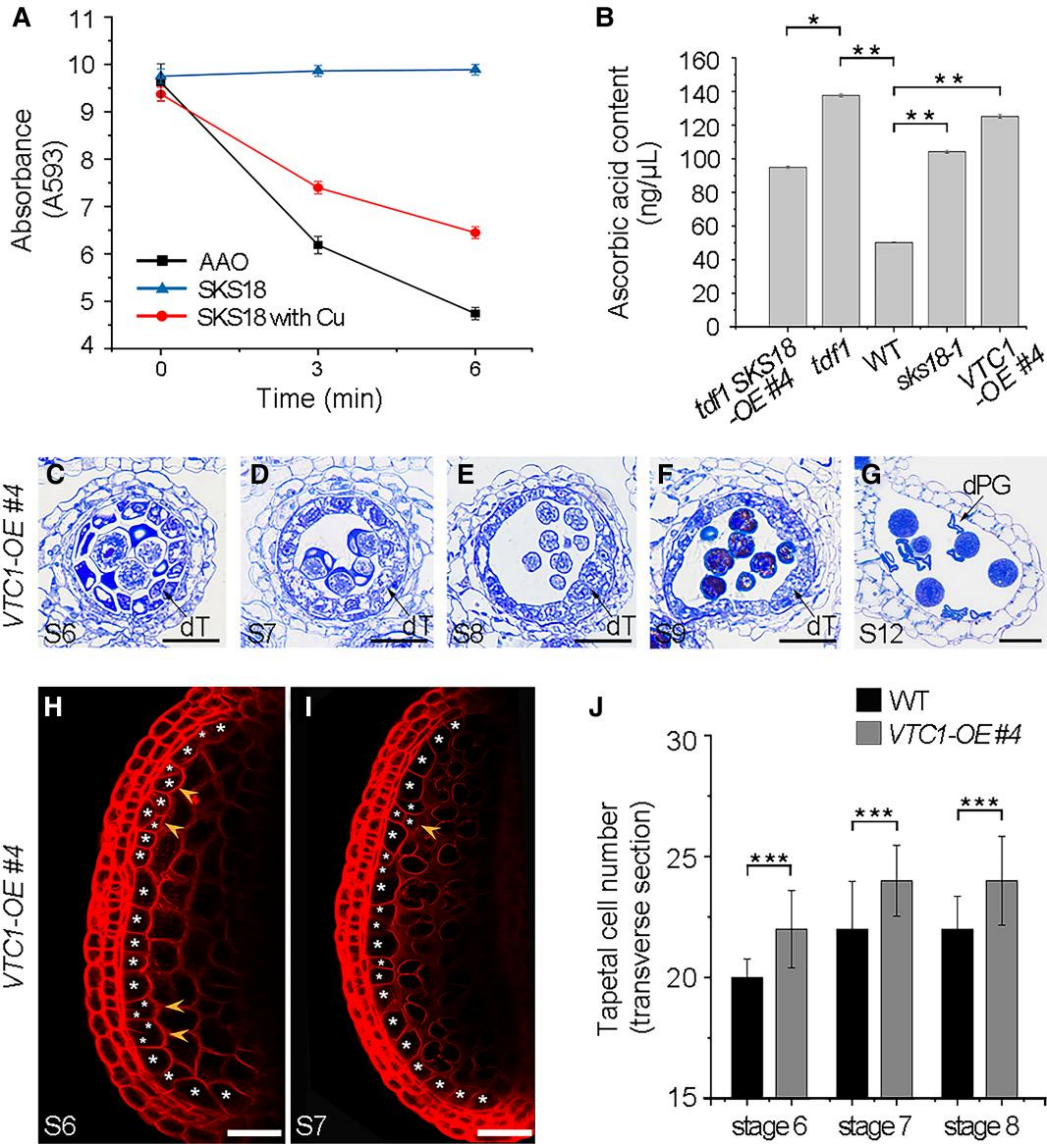
SKS18 shows ascorbate oxidase activity and high AsA contents lead to supernumerary tapetal cell divisions. A) Enzyme activity assay of recombinant SKS18-His. Purified SKS18 and empty vector proteins were incubated with a certain level of AsA and 10 μM CuSO_4_. AAO was used as a positive control. Data are means ± SD from 3 biological replicates. B) Measurement of AsA concentrations in inflorescences from WT, *tdf1*, *sks18-1*, *tdf1 SKS18-OE #4*, and *VTC1-OE #4* plants. Data are means ± SD from 3 biological replicates. *P < 0.05; **P < 0.01 (*t*-test). C–G) Semi-thin sections of anthers from *VTC1-OE #4* plants at the indicated stages. Scale bars, 20 μm. H and I) One abaxial locule in anther was stained with FM4-64 and extra tapetal cells (arrowheads) are present in *VTC1-OE #4* plants at Stages 6 to 7. Scale bars, 50 μm. J) Tapetal cell number based on the FM4-64 staining in WT and *VTC1-OE #4* plants from Stages 6 to 8. Data are means ± SD. *n* = 30 anthers. ***P < 0.001 (*t*-test). dT, defective tapetum; dPG, degenerated pollen grain.

*SKS18* encodes an MCO-like protein with a conserved copper-binding site (Sedbrook et al. 2002), although the predicted biochemical function of its encoded protein has not been experimentally verified. AAO belongs to 1 subgroup of MCOs (Ryden and Hunt 1993). AAO catalyzes the oxidation of AsA with the concomitant reduction of oxygen to water (Arrigoni et al. 1981). In maize root tips, a high level of AAO in QC cells is thought to be responsible for their low mitotic activity (Kerk and Feldman 1995). Additionally, AAO is predominantly present in the apoplast of land plants, which is consistent with the apoplast localization of SKS18 (Fig. 2P). Based on the above evidence, we hypothesized that SKS18 might have AAO activity to control tapetal cell divisions by oxidizing AsA. To test this hypothesis, we produced recombinant histidine (His)-tagged SKS18 (SKS18-His) from *E. coli* (Supplemental Fig. S9, A and B). As an antioxidant, AsA reduces Fe^3+^ to Fe^2+^, which results in a colored (593 nm) product (Buettner and Jurkiewicz 1996). AAO activity is associated with a decrease in absorbance at 593 nm. Compared with the activity of purified AAO, SKS18-His induced no colorimetric change (Fig. 5A). Previous studies had shown that the AAO activity of pumpkin (*Cucurbita* spp.) callus markedly increased by adding copper (Esaka et al. 1988, 1992), hinting that SKS18 might require a copper cofactor. We thus incubated recombinant SKS18-His or proteins purified from the empty vector control with 10 μM CuSO_4_. Notably, absorbance at 593 nm gradually decreased in the presence of SKS18 with copper added (Fig. 5A), indicating that SKS18 behaves as an AAO with a copper cofactor in vitro.

If SKS18 has AAO activity, the *sks18* mutant might exhibit a defect in AsA metabolism. To test this idea, we measured the concentration of AsA in inflorescences from homogenates of WT, *tdf1*, *sks18-1*, and *tdf1 SKS18-OE #4* plants. The levels of AsA in *sks18-1* were 2-fold higher than in the WT. Similarly, we detected high levels of AsA in *tdf1*. Moreover, AsA levels in *tdf1 SKS18-OE #4* were lower than those in *tdf1*, although they were still higher than those from WT (Fig. 5B). These results indicate that high AsA levels are negatively correlated with *SKS18* expression levels. We tested the AsA-deficient *vtc1* mutant in Arabidopsis as a negative control. In *vtc1-3* inflorescences, we measured a low concentration of AsA (Supplemental Fig. S10A). We confirmed these results via a quantitative analysis of AsA by liquid chromatography-tandem mass spectrometry (LC-MS/MS) (Supplemental Fig. S10B). Therefore, these results suggest that SKS18 can oxidize AsA to regulate AsA metabolism in vivo.

### High AsA concentrations lead to supernumerary cell divisions in tapetum

Based on the above results (Figs. 4Y and 5B), we suspected that increased concentrations of AsA might lead to extra divisions of tapetal cells. VTC1 plays a critical role in the AsA biosynthetic pathway (Conklin et al. 1999). To date, the regulation of AsA biosynthesis has been shown mainly to occur at the VTC1-catalyzed step in Arabidopsis (Zhang et al. 2012; Wang et al. 2013; Sawake et al. 2015). We constructed a *VTC1* overexpression vector and introduced it in WT plants. Of the *DYT1550pro:VTC1-GFP* (named *VTC1-OE*) transgenic lines generated, 2 independent lines (*VTC1-OE #4* and *VTC1-OE #5*) with high *VTC1* expression showed compromised pollen viability (Supplemental Fig. S11, A–C). Moreover, we observed a partial multilayered tapetum in both lines (Fig. 5, C–G and Supplemental Fig. S11, D–G). In *VTC1-OE #4* anthers, VTC1-GFP fluorescence was present in the tapetum (Supplemental Fig. S11, H–K). We also measured high levels of AsA in *VTC1-OE #4* inflorescences (Fig. 5B), confirming that overexpression of *VTC1* in the tapetum generated more AsA. Notably, *VTC1-OE #4* anthers displayed an over-proliferation of tapetal cells (Fig. 5, H–J). All these results indicate that increasing AsA concentrations perturb tapetum differentiation with excessive cell divisions that subsequently affect male fertility.

### TDF1 is a negative regulator of AsA accumulation for the control of cell division and subsequent differentiation of tapetum

**Figure 6:**
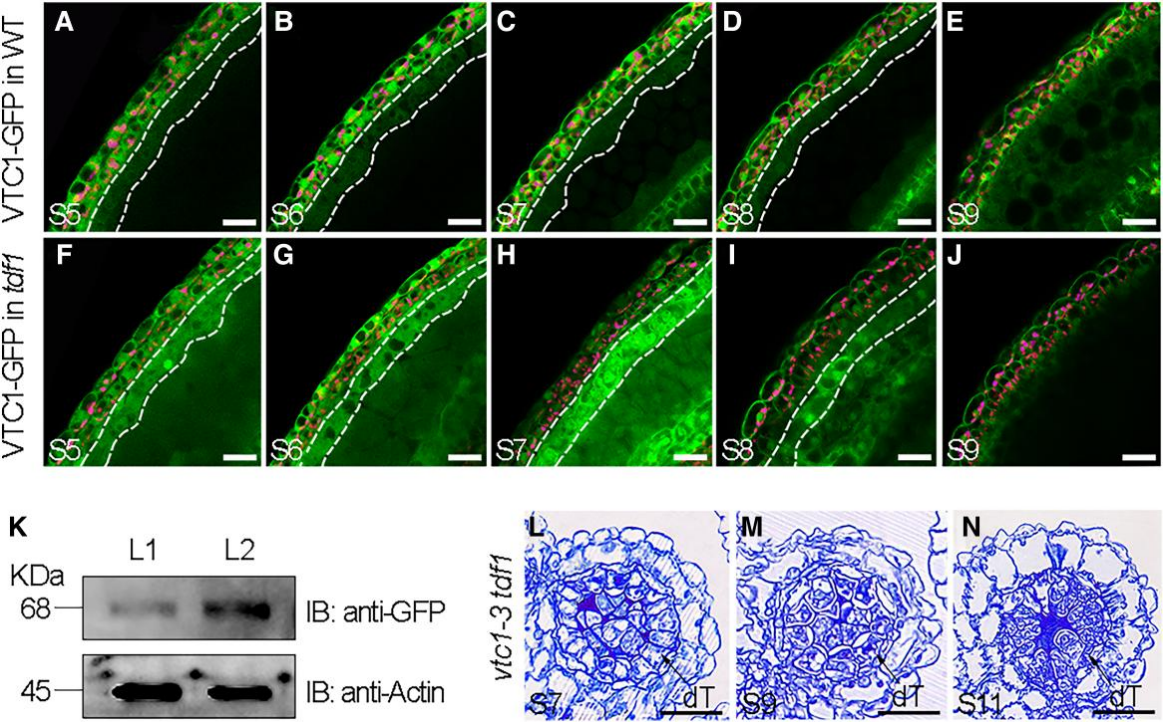
TDF1 is a negative regulator of AsA accumulation to control cell division and differentiation in tapetum. A–E) VTC1-GFP fluorescence localizing to epidermis, endothecium, and tapetum (dotted lines) from Stages 5 to 9. F–J) VTC1-GFP fluorescence is largely elevated in the *tdf1* defective tapetum (dotted lines). Chloroplast autofluorescence is shown in magenta. K) Immunoblotting of VTC1-GFP abundance with anti-GFP antibodies in total proteins extracts from *gVTC1pro:VTC1-GFP* in WT (L1) and *tdf1 gVTC1pro:VTC1-GFP* transgenic (L2) plants. Anti-β-actin was used as a loading control. L–N) Semi-thin sections of anthers from the *vtc1-3 tdf1* double mutant at the indicated stages. Scale bars, 20 μm. dT, defective tapetum.

AsA content represents the balance of its biosynthesis and metabolism. According to the severe phenotype of *tdf1* (Figs. 1M and 5B), we asked whether the AsA biosynthetic pathway in *tdf1* is also altered. *VTC1* expression in WT and *tdf1* showed no obvious difference during early anther development (Supplemental Fig. S12). We also created the translational reporter *VTC1pro:VTC1-GFP* and determined the spatiotemporal pattern of the encoded fusion protein in WT and *tdf1* anthers. In *VTC1pro:VTC1-GFP* anthers, from Stages 5 to 9, we detected VTC1-GFP fluorescence predominantly in the epidermis and endothecium with a stable intensity (Fig. 6, A–E). However, fluorescence changed in tapetal cells. We first detected a weak signal in the tapetum at Stage 5 (Fig. 6A), which was later followed by a relatively strong signal in tapetal cells along with their transition to the secretory type (Fig. 6, B–D). At Stage 9, the signal had filled the locule surrounding microspores (Fig. 6E). These results showed that VTC1 predominantly accumulates in the anther wall. By contrast, in *tdf1 gVTC1pro:VTC1-GFP* anthers, VTC1-GFP signal intensity was much higher in defective tapetal cells during Stages 5 to 7 relative to WT (Fig. 6, F–H), although it also remained in the epidermis and endothecium. Furthermore, VTC1-GFP fluorescence was widely present in microsporocytes and defective tetrads (Fig. 6, F–I). Tapetum and locules produced no fluorescence from VTC1-GFP at a late stage (Fig. 6J). We confirmed these observations with an antibody against GFP in extracts prepared from inflorescences of the above transgenic lines: VTC1 clearly accumulated to higher levels in the *VTC1-GFP* lines in the *tdf1* background compared with WT lines (Fig. 6K). These data indicate that TDF1 negatively governs the accumulation of VTC1.

To explore the genetic relationship between *TDF1* and *VTC1*, we crossed the *vtc1-3* and *tdf1* mutants. In the *vtc1-3 tdf1* double mutant, the defective phenotype of tapetal cells was abolished (Fig. 6, L–N and Supplemental Fig. S7E), which was reminiscent of the *tdf1* lines rescued by overexpressing *SKS18* (Fig. 4, P–T and Supplemental Fig. S7, C and D). These observations substantiate the notion that TDF1 functions as a negative regulator of AsA accumulation to control cell division and/or differentiation in the tapetum.

### Lower ROS levels by excessive AsA induce increased cell divisions in tapetum

**Figure 7:**
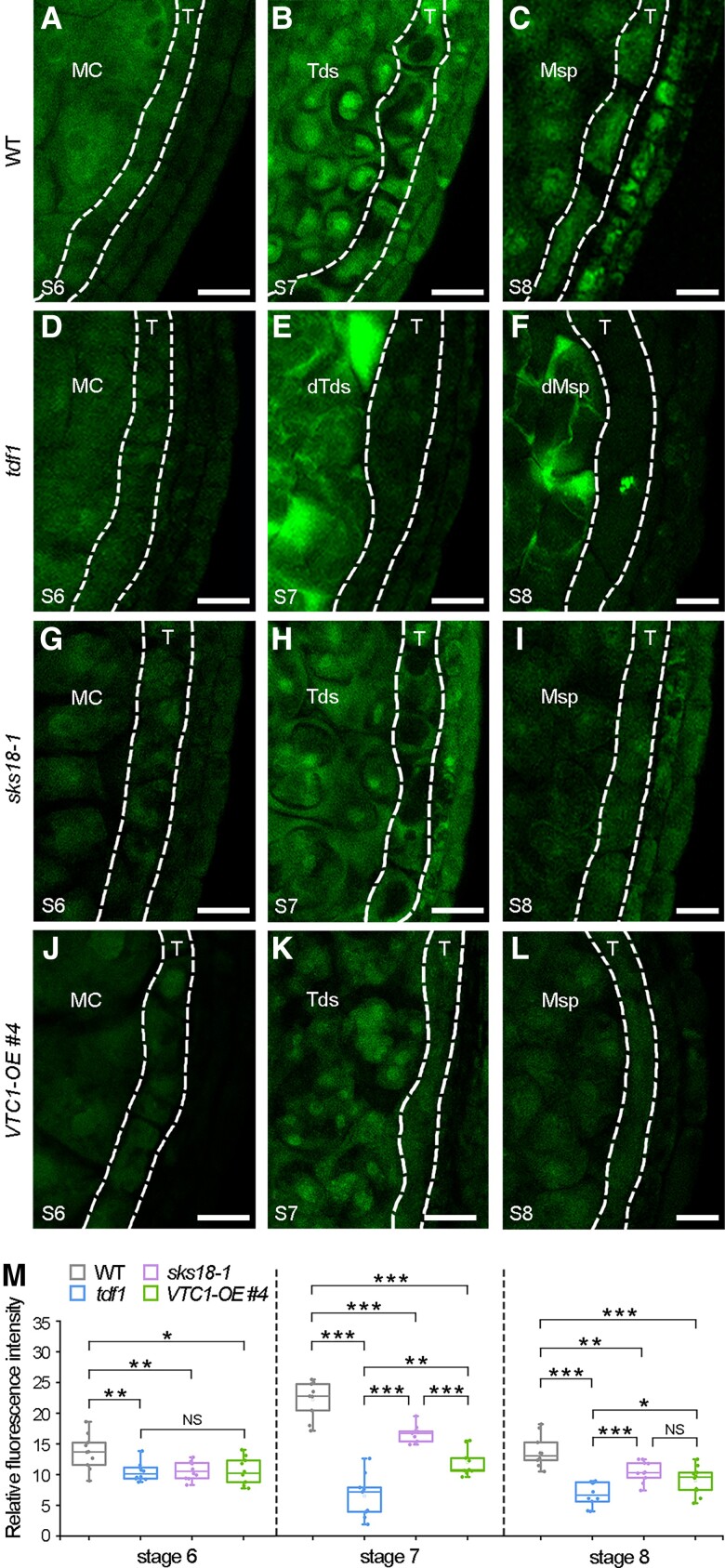
Analyses of H_2_O_2_ in tapetum from WT, *tdf1*, *sks18-1*, and *VTC1-OE* anthers. H_2_DCF-DA staining analysis of H_2_O_2_ in tapetum (dotted lines) from WT (A–C), *tdf1* (D–F), *sks18-1* (G–I), and *VTC1-OE #4* (J–L) anthers at Stages 6 to 8. Scale bars, 20 μm. M) Fluorescence quantification of H_2_O_2_ levels in tapetum from WT, *tdf1*, *sks18-1*, and *VTC1-OE #4* at Stages 6 to 8 based on H_2_DCF-DA staining. Data are shown as boxplots, with the box representing the interquartile range and the central line indicating the median; the Tukey-style whiskers extend to a maximum of 1.5× interquartile range from the 25th and 75th percentiles. *n* = 10 (anthers at Stage 6), *n* = 9 (anthers at Stage 7), and *n* = 10 (anthers at Stage 8). *P < 0.05; **P < 0.01; ***P < 0.001; NS, not significant (*t*-test). MC, meiocytes; Msp, microspore; T, tapetum; Tds, tetrads; dT, defective tapetum; dTds, defective tetrads; dMsp, defective microspore.

In plants, AsA is the major antioxidant buffer that scavenges ROS to modify the redox state of the cell via a nonenzymatic mechanism (Noctor and Foyer 1998). Considering the fact that elevated H_2_O_2_ levels are required for cell differentiation and the accumulation of superoxide ($O_{2}^{-}$) maintains cell proliferation in the root (Tsukagoshi et al. 2010; de Simone et al. 2017), we asked whether increased levels of AsA affected ROS accumulation and whether these changes in ROS levels might affect cell division and/or differentiation in the tapetum. Accordingly, we measured anther H_2_O_2_ levels with 2′, 7′-dichlorodihydrofluorescein diacetate (H_2_DCF-DA) staining (Fichman et al. 2019). In WT anthers, H_2_O_2_ levels in the tapetum gradually increased from Stages 6 to 7 and slightly declined at Stage 8 (Fig. 7, A–C and M). However, in *tdf1* anthers, we detected significantly lower H_2_O_2_ levels in the tapetum from Stages 6 to 8 (Fig. 7, D–F and M). Moreover, we detected high H_2_O_2_ levels in the defective tetrads of *tdf1* (Fig. 7, E and F). Looking at *sks18-1* and *VTC1-OE #4* anthers, their tapetal H_2_O_2_ levels were lower than in WT (Fig. 7, G–M), but compared with *tdf1*, their H_2_O_2_ levels tended to increase at Stages 7 to 8 (Fig. 7, H–M). Combined with the increased AsA contents of these mutants (Fig. 5B), these results indicate that excess AsA leads to a pronounced drop in H_2_O_2_ levels in the tapetum.

We measured $O_{2}^{-}$ levels via dihydroethidium (DHE) staining. In both WT and *tdf1* anthers, the $O_{2}^{-}$ levels in the tapetum were similar at Stage 6. The $O_{2}^{-}$ levels then decreased in WT but remained high in *tdf1* at Stage 7 (Supplemental Fig. S13). Therefore, both increasing $O_{2}^{-}$ levels and decreasing H_2_O_2_ levels led to tapetal cell division, which agreed with the influences of ROS in the root (Tsukagoshi et al. 2010). As H_2_O_2_ levels varied much more than those of $O_{2}^{-}$ in *tdf1* (Fig. 7M and Supplemental Fig. S13G), we speculated that H_2_O_2_ levels played a dominant role in repressing tapetal cell division.

**Figure 8:**
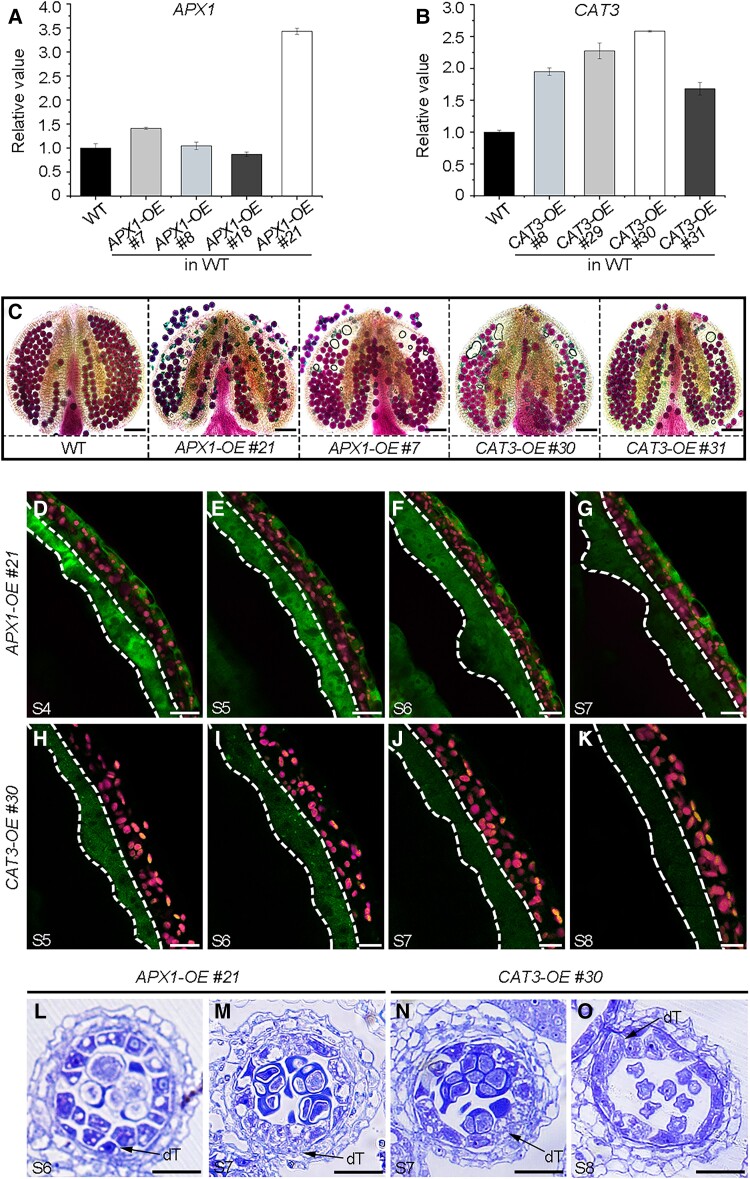
Multilayered tapetum is caused by overexpressing genes encoding ROS-scavenging enzymes. Expression levels of *APX1* (A) and *CAT3* (B) in inflorescences from WT and different transgenic plants by RT-qPCR analysis. Data are means ± SD. Three biological repeats were performed, with similar results. Each biological replicate was performed with 3 technical replicates. C) Alexander's staining of anthers from WT with viable pollen grains, stained purple, and anthers from transgenic plants overexpressing genes encoding ROS scavenging enzymes with remnants of aborted pollen, stained green. Scale bars, 20 μm. D–G) Fluorescence of APX1-GFP in *DYT1550pro:APX1-GFP* transgenic plants is mainly present in tapetum (dotted lines) at Stages 4 to 7. H–K) Fluorescence of CAT3-GFP in *DYT1550pro:CAT3-GFP* transgenic plants is mainly present in tapetum (dotted lines) at Stages 5 to 8. Chloroplast autofluorescence is shown in magenta. Semi-thin sections of anthers from *APX1-OE #21* (L and M) and *CAT3-OE #30* transgenic plants (N and O) at the indicated stages. Scale bars, 20 μm. dT, defective tapetum.

ASCORBATE PEROXIDASE 1 (APX1) and CATALASE 3 (CAT3) are 2 important H_2_O_2_ scavenging enzymes in plants (Frugoli et al. 1996; Asada 1997). To address whether low ROS levels lead to high mitotic activity in the tapetum, we individually overexpressed *APX1* or *CAT3* in WT plants using the *DYT1550* promoter. We examined 15 *DYT1550pro:APX1-GFP* (named *APX1-OE*) transgenic lines. Among these lines, *APX1-OE #21* had a higher expression of *APX1* than the WT, showing severe pollen abortion (Fig. 8, A and C). Similarly, 3 of the 13 *DYT1550pro:CAT3-GFP* (named *CAT3-OE*) lines exhibited compromised male sterility with the higher expression of *CAT3* (Fig. 8, B and C). We detected both APX1-GFP and CAT3-GFP mainly in the tapetum (Fig. 8, D–K). Cytological data further demonstrated that these transgenic plants have a multilayered tapetum reminiscent of *tdf1*, *sks18*, and *VTC1-OE* plants (Figs. 1L, 4, F–H, 5, C–E, and 8, L–O), suggesting that insufficient ROS promote mitotic activity in the tapetum.

We also overexpressed *SKS18* in WT plants. Among these *DYT1550pro:SKS18-GFP* transgenic lines (named *SKS18-OE*), *SKS18-OE #30* had the highest expression level of *SKS18* (Supplemental Fig. S14A). We measured a low level of AsA in *SKS18-OE #30* inflorescences (Supplemental Fig. S14B), confirming that overexpression of *SKS18* reduced the amount of AsA. In *SKS18-OE #30* anthers, H_2_O_2_ levels in the tapetum were higher than those in WT (Supplemental Fig. S14, C and D). FM4-64 staining indicated that the size of tapetal cells is enlarged and the number of tapetal cells decreases in *SKS18-OE #30* relative to WT (Supplemental Fig. S14, E and F), suggesting that excessive ROS repress mitotic activity and induce cell expansion in the tapetum. These results confirm that appropriate AsA-mediated ROS scavenging constrains cell divisions in the tapetum that are necessary for tapetal differentiation.

## Discussion

**Figure 9:**
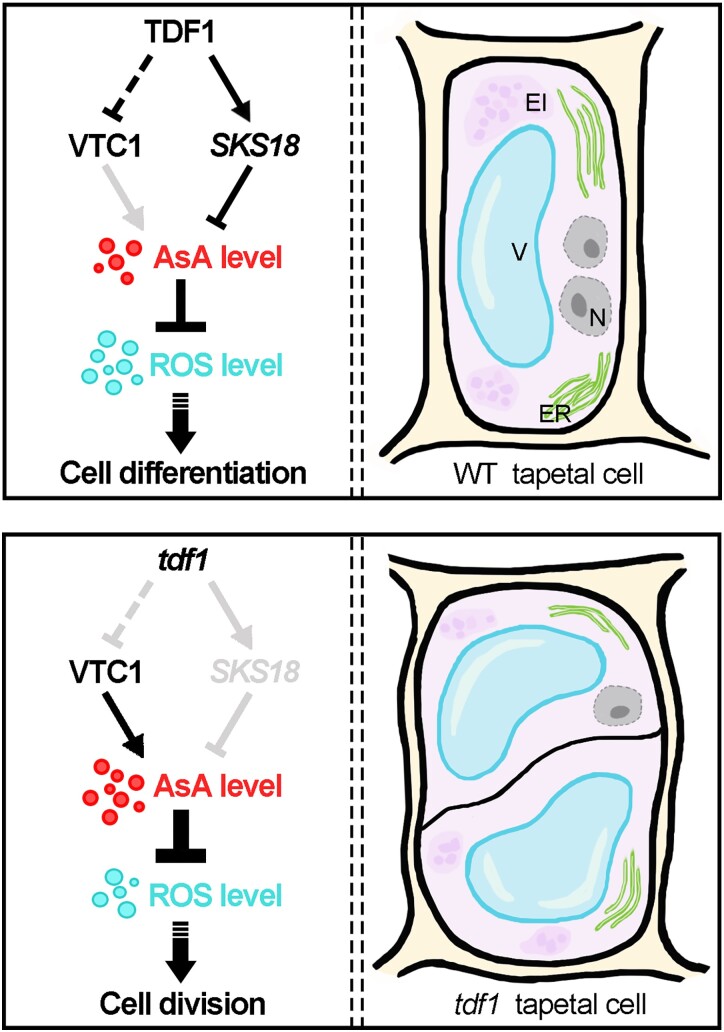
AsA-mediated ROS homeostasis balances tapetal cell division and cell differentiation through a TDF1-dependent regulatory module. Schematic model depicting a molecular circuit that balances cell division and cell differentiation in tapetum required for late pollen development. In this model, TDF1 is a negative regulator of AsA content via activating *SKS18* expression for AsA oxidation and controlling VTC1 accumulation for AsA biosynthesis. The appropriate ROS homeostasis in tapetum is supervised by AsA levels, which promote tapetal cells transition from cell proliferation to cell differentiation. EI, elaioplast; ER, endoplasmic reticulum; N, nucleus; V, vacuole.

After cell fate determination, the tapetum undergoes cell differentiation to initiate its secretory role for microspores and pollen development in both monocots and dicots. In maize, MS32, a basic helix-loop-helix (bHLH) TF, is essential for restricting cell divisions after tapetal cells are specified (Moon et al. 2013). The ortholog of *MS32* in Arabidopsis is *DYT1*. However, although DYT1 is critical for early tapetum development, the *dyt1* mutant shows no extra cell divisions (Nan et al. 2017). The data presented here showed that *TDF1*, a direct target of DYT1, acts as a switch that triggers exit from cell division and entry into cell differentiation in the tapetum (Fig. 9). The initiation of tapetal differentiation involves coordinated changes in the nucleus, cytoplasm, and cell wall. Endoreduplication is postulated to support the necessary high metabolic activity (Nagl 1976; Edgar and Orr-Weaver 2001). One nuclear division of a WT tapetal cell occurs without cytokinesis following the meiotic stage, giving rise to 2 nuclei through the endoreduplication cycle (Fig. 1, A and B). However, tapetal cells in *tdf1* usually have disordered cytokinesis (Fig. 1O), randomly exiting from the endocycle into the mitotic cycle (Fig. 1, J, L, and M). Consistently, mutations in putative genes involved in the switch from cell division to differentiation usually cause cellular hypertrophy (Faure et al. 1998; Frank et al. 2002; Sieberer et al. 2003), which is in agreement with the hypertrophic tapetal cells observed in *tdf1* (Zhu et al. 2008; Fig. 1, E–H). Moreover, TDF1 likely affects tapetal cell wall loosening by activating the expression of *EXPB5*, encoding a beta-expansin family protein, to permit turgor-driven cell enlargement (Lou et al. 2018). Therefore, this evidence supports the idea that TDF1 promotes tapetal differentiation by inhibiting cell divisions to guarantee subsequent tapetum function.

We previously showed that TDF1 has 828 candidate target genes, among them were several *SKS* genes (Lou et al. 2018). Here, we confirmed *SKS18* as a direct transcriptional target of TDF1 and that the TDF1-SKS18 module functions in tapetum differentiation (Figs. 2–4). The SKS family is classified as a subgroup of MCOs that catalyzes the 4-electron reduction of molecular oxygen to water with concomitant oxidation of a substrate (Ryden and Hunt 1993). In contrast to MCOs with 3 conserved copper-binding sites, SKS proteins are monocopper oxidases due to the absence of 2 copper center motifs, making their biochemical function unclear (Sedbrook et al. 2002). MCOs are a family of enzymes comprising AAO, laccases, ferroxidases, and ceruloplasmin. AAO catalyzes the oxidation of AsA. Our biochemical data showed that the mutation of SKS18 led to high AsA concentrations in inflorescences (Fig. 5B and Supplemental Fig. S10B), which agrees with the observation that AAO activity is negatively associated with AsA contents in rice panicle and maize roots (Reddy et al. 1986; Kerk and Feldman 1995). Additionally, we demonstrated that SKS18 enzyme activity is depended on a copper cofactor (Fig. 5A). All these results demonstrated that TDF1 activated the expression of *SKS18*, whose encoding enzyme functions as an AAO with a copper cofactor to regulate AsA oxidization.

The SKS family counts 19 members in Arabidopsis (Sedbrook et al. 2002). A recent study indicated that mutating the last histidine residue in the copper center of SKS11 produced a protein with enough activity to rescue the defects of the *sks11 sks12* double mutant (Duan et al. 2021). We, therefore, suspect that some SKS proteins may have evolved new functions that do not require their copper cofactor, while others like SKS18 behave as AAOs and rely on copper stimulation. CLUSTERED PRIMARY BRANCH 1 (CPB1), which has high sequence similarity to SKU5, is a copper-binding protein in maize (Shimomura 2006). Alternatively, other amino acid residues might be involved in copper binding in addition to histidine. In the case of galactose oxidase (GO), a monocopper oxidase, copper is ligated by histidine and tyrosine residues. Tyr-272 creates a radical-forming cofactor via a thioether bond, which is the active and oxidized state of GO (Ito et al. 1991). In the amino acid sequences of SKS members, a tyrosine residue is located 3 residues downstream from the conserved histidine (Supplemental Fig. S15), suggesting that these tyrosine residues might play a role in copper binding.

As a major regulator for AsA biosynthesis, *VTC1* is strictly regulated at the transcriptional, translational, and posttranslational levels (Zhang et al. 2009, 2012; Sawake et al. 2015). We showed that TDF1 negatively affected VTC1 accumulation rather than that of *VTC1* transcripts in anthers (Fig. 6, F–K and Supplemental Fig. S12). Since TDF1 is a transcriptional activator (Feng et al. 2012), we considered 2 possible explanations for how TDF1 represses VTC1 accumulation. A previous study showed that the photomorphogenic factor CONSTITUTIVE PHOTOMORPHOGENIC 9 (COP9, subunit 5B of the signalosome [CSN5B]) interacts with VTC1 in seedlings, which promotes ubiquitination-dependent VTC1 degradation through the 26S proteasome in the dark (Wang et al. 2013; Li et al. 2016). Given that genes related to protein degradation are enriched in TDF1-specific genes (Li et al. 2017a), we, therefore, hypothesized that TDF1 might mediate VTC1 degradation via regulating those genes involved in the ubiquitin/26S proteasome system. Alternatively, Zinc finger 3 (SlZF3), a tomato (*Solanum lycopersicum*) Cys2/His2-type zinc-finger protein, directly binds to CSN5B to allow the accumulation of VTC1 (Li et al. 2018). The loss of TDF1 function may, therefore, prevent VTC1 degradation by over-accumulating proteins that competitively interact with CSN5B. As TDF1 regulated *SKS18* expression (Fig. 3), we propose TDF1 as a negative regulator of AsA accumulation via controlling AsA biosynthesis and AsA oxidation pathways.

AsA acting as a redox buffer in plant cells is one of its most important attributes (Foyer and Noctor 2005). We presented evidence that demonstrated how SKS18 modulated ROS levels via lowering AsA contents (Figs. 5, A and B, 7, G–I, Supplemental Figs. S10, B and S14, B–D), in agreement with the synergistic regulation of redox state by AAO and AsA in plants (Pignocchi and Foyer 2003). Functions for a number of SKS proteins have been explained (Sedbrook et al. 2002; Jacobs and Roe 2005; Zhou 2019); in all cases, they were related to the redox state of the cell. For instance, ZmSKS13 influences kernel development through modulating ROS homeostasis (Zhang et al. 2021). In Arabidopsis, SKS11 and SKS12 are required for pollen tube integrity, growth, and guidance by regulating ROS levels (Duan et al. 2021). Pollen-expressed *SKS13* affects pollination/fertilization (Ji et al. 2019). Moreover, overexpression of *SKS13* in leaves caused a decline in aphid populations predating on plants, possibly due to the accumulation of ROS (Chen et al. 2014). A higher AsA concentration has been reported to lead to vigorous aphid fecundity by decreasing ROS levels (Kerchev et al. 2012). We, therefore, infer that other SKS proteins might also have AAO activity to maintain the redox balance via controlling AsA contents; these SKS members may exhibit a functional redundancy with SKS18 during tapetum development.

As signaling molecules, ROS are crucial for cell growth in anthers; importantly, ROS spatiotemporal pattern is conserved in both Arabidopsis and rice (Xie et al. 2014; Zhang and Yang 2014). During the formation of the 4-lobed anther pattern, ROS are hardly detectable (Yang et al. 2016, 2018). Following microsporocyte entry into meiosis, ROS initially accumulated in anthers and gradually peaked in tapetal cells at the tetrad stage (Fig. 7, A–C). After tapetum entered PCD, ROS levels quickly fell and disappeared until mature pollen formation (Hu et al. 2011; Zhu et al. 2020). Many reports have shown that ROS exert multiple functions following their spatiotemporal changes during anther development. Based on these data, we speculated that the reduction state is suitable for specifying anther morphology before meiosis, similar to hypoxia triggering germ cell fate in maize (Kelliher and Walbot 2012). Following meiosis, an oxidized cellular state takes center stage and triggers tapetal cell differentiation (Fig. 7, A–C) and PCD (Hu et al. 2011; Luo et al. 2013; Xie et al. 2014; Zheng et al. 2019). Finally, the return of ROS is essential for pollen germination and pollen tube growth (Luria et al. 2019; García-Quirós et al. 2020). Importantly, the range of ROS produced plays an essential role in tapetum development. Indeed, dropping below the normal ROS range in tapetum during Stages 6 to 8 will disturb tapetal cell differentiation (Figs. 4, 7, and8). There was more compromised pollen due to the defective tapetum in *VTC1-OE #4* than in *sks18* (Supplemental Figs. S6 and S11). Considering that ROS levels in *VTC1-OE #4* were lower than in *sks18* anthers, especially at Stage 7 (Fig. 7M), we suggest that tapetum differentiation is more susceptible to the ROS range at the tetrad stage than at other developmental stages, and falling below an ROS threshold may be associated with male sterility. Given that the AsA-mediated ROS scavenging system is monitored by the TDF1-dependent regulatory module (Fig. 9), our findings reveal that this molecular surveillance achieves a local ROS generation/accumulation cycle that ensures successful tapetum development and mature pollen production.

## Materials and methods

### Plant materials and growth conditions

The Arabidopsis (*A. thaliana*) Columbia-0 (Col-0) and Landsberg *erecta* (Ler) accessions were used in this study. The homozygous *tdf1* (Zhu et al. 2008) and *ams* (Sorensen et al. 2003) mutants were described previously. All other mutants were obtained from the Arabidopsis Biological Resource Center (ABRC): *sks18-1* (WiscDsLoxHs032_08H) and *vtc1-3* (SAIL_611_D10.v1). The double mutant, *vtc1-3 tdf1*, was generated by a genetic cross. Primer sequences for genotyping are listed in Supplemental Data Set S1. Arabidopsis seeds were stratified for 3 d at 4 °C in the dark and sown on a mixture of vermiculite and nutritive soil (1:1) for growth at a light intensity of approximately 100 μmol m^−2^ s^−1^ (Philips Lifemax Cool White fluorescent bulbs) under long-day conditions (16-h light/8-h dark) in a growth room at 24 °C. For the measurement of AsA levels, *vtc1-3* was grown under short-day conditions as described (Veljovic-Jovanovic et al. 2001).

### Cytological analysis

Individual anthers were dissected and stained with Alexander's solution in darkness for 2 h at room temperature (Alexander 1969). The stained anthers were visualized and photographed with an Olympus BX51 microscope (Olympus, http://www.olympus-global.com). For semi-thin sections, inflorescences from different genotypes were fixed for 2 to 3 d in FAA (ethanol 50%, acetic acid 5%, formaldehyde 3.7%, all v/v) and were embedded in Spurr's resin. Semi-thin sections (1 μm in thickness) were stained in a 0.01% (w/v) toluidine blue/sodium borate solution at 45 °C for 5 min. After washing off the excess solution with water, the sections were photographed with an Olympus BX51 microscope in a bright field.

For TEM, buds from WT and *tdf1* inflorescences were placed in ice-cold 2.5% (v/v) glutaraldehyde in 10 mM phosphate buffer (pH 7.4). Samples were postfixed in 1% (w/v) osmium tetroxide and dehydrated in an ethanol/water series (30%, 50%, 75%, 85%, 90%, 95%, and 100%, all v/v). Samples were dehydrated twice with 100% propylene oxide and subsequently transferred to propylene oxide/Spurr's resin mixtures with ratios of 1:1, 1:3, and 3:1 (v/v), respectively. Later, samples were embedded in Spurr's resin and polymerized at 65 °C for 48 h. Ultrathin sections (50 to 70-nm thick) were stained in a solution of uranyl acetate and lead citrate. The images were viewed by a JEM-1230 TEM.

For anther layer observations, anthers were dissected from inflorescences and incubated in 10 μM FM4-64 (Thermo Fisher, USA) solubilized in 10% (v/v) glycerol for 2 to 3 h at 4 °C in the dark. Samples were subsequently mounted on slides and visualized with an Olympus FV3000 laser scanning microscope. For monitoring of FM4-64 signal, the 514-nm laser line was used for excitation and a 620- to 680-nm bandpass filter was used for detection. For analysis of tapetum development, semi-thin sections were orderly stained with toluidine blue (10 mg/mL) for 5 min, tinopal (10 μg/μL) for 15 min, and 3,3′-diethyloxacarbocyanine iodide (DiOC_2_, 5 μL/mL) for 5 min as described (Lou et al. 2014). Images were captured with an Olympus BX51 fluorescence microscope.

### ROS staining and quantification in anthers

2′,7′-dichlorodihydrofluorescein diacetate (H_2_DCF-DA, Sigma-Aldrich, USA) staining of H_2_O_2_ in anthers was carried out as described (Xie et al. 2014). Individual anthers were dissected and submerged in 5 μM H_2_DCF-DA staining solution and vacuum-infiltrated for 5 min. Anthers were subsequently incubated at 25 °C for 2 to 3 h. DHE (Invitrogen, USA) staining of $O_{2}^{-}$ was performed as previously described (Tsukagoshi et al. 2010). Individual anthers were submerged in 10 μM DHE staining solution and vacuum-infiltrated for 5 min. Anthers were subsequently incubated at 25 °C for 2 to 3 h in the dark. The undamaged samples were washed in water and immediately observed on slides with an Olympus FV3000 laser scanning microscope. H_2_DCF-DA fluorescence was visualized by a filter set with 488 nm excitation and 498 to 532 nm bandpass. DHE fluorescence was visualized by a filter set with 518 nm excitation and 606 nm bandpass. The fluorescence intensities were quantified using ImageJ software. Based on the ROS fluorescent images, tapetal ROS intensities represent the mean value from all tapetal cells in 1 abaxial locule, calculated from about 10 independent images. Experiments were performed at least 3 times with different batches of plants. ROS fluorescent images used to produce these data are classified into 7 groups and provided in Supplemental Data Set S3.

### Generation of transgenic plants

To generate the complementation construct, a 3,797-bp genomic fragment, which included a 2,406-bp genomic fragment without stop codon of *SKS18* and a 1,391-bp fragment upstream from the initiation codon, was amplified from Col-0 genomic DNA by KOD polymerase (Takara, Japan). The resulting amplicon was digested by restriction enzymes (Takara, Japan) and ligated into a modified GFP-pCAMBIA1300 vector (CAMBIA, Australia). This construct was transformed into *sks18* homozygous plants. To obtain the overexpressing lines, the genomic sequences of *SKS18*, *VTC1*, *APX1,* and *CAT3* (without stop codons) were individually amplified and recombined into a modified GFP-pCAMBIA1300 vector, where their expression was driven by the *DYT1* promoter (550 bp) (Gu et al. 2014). These constructs were transformed into Col-0, *tdf1*/*TDF1* and *ams*/*AMS* plants, respectively. For expression analysis, the genomic sequence of *KEULE* excluding the stop codon plus its native promoter (1,514 bp) was amplified and cloned into the modified GFP-pCAMBIA1300 vector. This construct was transformed into Col-0 and *tdf1*/*TDF1* plants, respectively. The genomic sequence of *VTC1* without the stop codon plus its native promoter (1,581 bp) was amplified and cloned into the modified GFP-pCAMBIA1300 vector. This construct was transformed into Col-0 and *tdf1*/*TDF1* plants. The genomic sequence of *TDF1* including the stop codon plus its native promoter (816 bp) was amplified and cloned into the modified VENUS-pCAMBIA1300 vector. This construct was transformed into Col-0 plants. All constructs were confirmed by DNA sequencing, introduced into Agrobacterium (*Agrobacterium tumefaciens*) strain GV3101, and transformed into plants by floral dipping (Clough and Bent 1998). All transformants were selected using 20 mg/L hygromycin and transferred to soil to check their genotype by PCR. Primer sequences are provided in Supplemental Data Set S1. At least 3 independent lines with the same relative expression levels of the transgene and localization pattern of the GFP fusion protein were selected for phenotypic and fluorescence analyses.

CRISPR/Cas9 vectors were constructed as previously described (Yan et al. 2015). Two designed single guide RNAs (sgRNAs) targeting *SKS18* were inserted into the AtU6-26-sgRNA vector. The sgRNA cassette was digested with the enzymes *Nhe*I and *Spe*I, and ligated into the *Spe*I site of the *ProYAO*:hSpCas9 construct to generate the CRISPR/Cas9 vector. This construct was transformed into WT plants via Agrobacterium-mediated floral dipping. T1 transgenic plants were confirmed by PCR and were crossed with their respective backgrounds to obtain *Cas9*-free plants with mutations in the T2 progeny.

### Total RNA isolation, RT-PCR and RT-qPCR analysis

Root, stem, rosette leaves, inflorescences after plant bolting, and 21-d-old seedlings were harvested and quickly frozen in liquid nitrogen. Total RNA was extracted with a Trizol kit (Invitrogen, USA). First-strand cDNA synthesis was performed using Fly First-Strand cDNA Synthesis SuperMix (TransGen, China) following the manufacturer's protocol. The transcript level of *SKS18* was measured by RT-PCR for 28 cycles. The qPCR reactions were carried out using a Real-time PCR System (Applied Biosystems) with a SYBR Green Real-time PCR Master Mix (Toyobo, Japan). At least 3 biological replicates and 3 technical replicates were performed for each combination of cDNA samples and primer pairs. *β-TUBULIN* served as an internal control. All PCR experiments were performed under the following conditions: 95 °C for 5 min, 40 cycles of 95 °C for 10 s, and 60 °C for 1 min. Under the same conditions, the ΔCt values were calculated and report the 2^−ΔCt^ values as the fold-enrichment relative to *β-TUBULIN*. The primer sequences are listed in Supplemental Data Set S1.

### RNA in situ hybridization

Inflorescences were fixed in FAA buffer containing 3.7% (v/v) formaldehyde and vacuum-infiltrated for 15 min on ice. Samples were dehydrated in a graded ethanol series and stained with safranine in xylene/ethanol solutions. Samples were placed into a 60 °C oven for 1 wk and finally embedded in Paraplast (Leica, Germany). Transverse sections of 8 μm in thickness were transferred onto poly-L-lysine coated glass slides (Sigma-Aldrich, USA) for hybridization. RNA in situ hybridization was performed using a Digoxigenin RNA Labeling Kit (Roche, USA). A 497-bp *SKS18* cDNA fragment and a 449-bp *VTC1* cDNA were amplified and cloned into the pBluescriptSK vector. These plasmids were individually digested by BamHI or EcoRI and used as templates. Sense and antisense probes were transcribed using the above templates by the T3 or T7 RNA polymerase (Roche, USA), respectively. The hybridization for *SKS18* transcripts in WT and *tdf1* anthers was performed in a single batch, while that in *ams* was performed in another batch. The hybridization for *VTC1* transcripts in WT and *tdf1* anthers was performed in a single batch. Primer sequences are provided in Supplemental Data Set S1.

### ChIP assay

The ChIP procedure was performed as described with minor modifications. One gram of closed buds from *tdf1 gTDF1pro:TDF1-GFP* complemented plants frozen in liquid nitrogen was crosslinked in 0.4 M sucrose-1% (v/v) formaldehyde buffer. Nuclei were isolated with extraction buffer and lysed with lysis buffer. The chromatin was sheared with sonication, resulting in most DNA fragments having a size between 200 and 800 bp. After pre-absorption using pre-immune serum with sheared salmon sperm DNA/protein A agarose mix (Millipore, USA) for 1 h, the DNA–protein complex was immunoprecipitated at 4 °C overnight using an anti-GFP monoclonal antibody (Millipore, USA) (1:100 dilution). Seventy microliters of magnetic beads coupled with protein G (Invitrogen, USA) were added to precipitate the antibody–protein/DNA complexes. After washing, the samples were incubated at 65 °C overnight to reverse the crosslinking. The co-precipitated DNA was purified and analyzed by qPCR as described above. Under the same conditions, the ΔCt values (Ct of each sample—Ct of the No antibody control) were calculated and reported 2^−ΔCt^ as the fold enrichment. Primer sequences are listed in Supplemental Data Set S1.

### Protein production and purification

The coding sequences of *TDF1* and *SKS18* were cloned into the pMAL-p5X vector (NEB, USA) and pCold TF DNA vector (TaKaRa, Japan), respectively. These constructs were transformed into *E. coli* Rosetta Competent Cells (Millipore, USA) for protein production and purification. The transformed cells were cultured at 37 °C until the OD600 reached 0.6 to 0.8; protein production was induced with 1 mM isopropyl-β-d-thiogalactopyranoside (IPTG) and growth at 18 °C overnight. Following centrifugation at 8,000×g for 5 min at 4 °C, cells were lysed by sonication on ice and supernatants were collected. For the MBP-TDF1 fusion protein, the supernatant was purified on Amylose resin (NEB, USA). For the SKS18-His fusion protein, the supernatant was purified on Ni-NTA resin (GE Healthcare, USA) according to the manufacturer's instructions. The purified proteins were quantified via SDS-PAGE and A280 absorbance using BSA as a standard, respectively. Primer sequences are listed in Supplemental Data Set S1.

### Determination of AsA level

AsA contents were measured using an ascorbic acid assay kit II (Sigma-Aldrich, USA). Freshly collected closed buds (anthers before Stage 12) (10 mg) were homogenized in cold Ferric Reducing/Antioxidant and Ascorbic Acid (FRASC) buffer. The homogenate was centrifuged at 13,000×g for 10 min at 4 °C and the supernatants were diluted 10 times in a final volume of 100 μL for AsA determination. To each sample, 10 μL of water was added, while 10 μL of ascorbic acid oxidase was added to the blank wells. The reaction was preincubated for 15 min at room temperature. A master reaction mix (80 μL FRASC buffer, 10 μL AsA probe, and 10 μL iron chloride solution) was added to each sample. After incubation for 2 min, AsA content was determined by measuring the absorbance at 593 nm. The value from the blank sample (containing AAO) was subtracted from all readings to correct for background. AsA levels were then calculated based on an AsA standard curve. Similar results were obtained in at least 3 biological replicates.

AsA contents were also measured using an Agilent 1290 Infinity II series UHPLC System (Agilent Technologies) equipped with Waters BEH C18 (2.1 × 100 mm, 1.7 μm, Waters). Freshly collected closed buds were incubated with 500 μL extraction solution (acetonitrile:methanol:water = 2:2:1, v/v/v) and sonicated for 5 min in an ice-water bath 3 times. After centrifugation at 13,400×g for 15 min at 4 °C, the supernatants from each sample were extracted and diluted 100-fold with extraction solution. Mobile phase A was 0.1% (v/v) formic acid in water and mobile phase B was acetonitrile. The gradient of mobile phase B was as follows (all v/v): 0 min, 5%; 2 min, 5%; 4 min, 90%; 6 min, 90%; 7 min, 5%; and 11 min, 5%. The flow rate was 300 μL/min. The column temperature was set to 35 °C and the auto-sampler temperature was set to 10 °C. The injection volume was 1 μL. An Agilent 6495 triple quadrupole mass spectrometer (Agilent Technologies), equipped with an AJS electrospray ionization (AJS-ESI) interface, was used for the assay. LC/MS-MS assays were performed by Lixinheng Technology Co., Ltd (Wuhan, China).

### Determination of SKS18 enzyme activity

Ascorbic acid oxidase activity was determined by monitoring the formation of Fe^2+^, which shows a decrease in absorbance at 593 nm due to AsA oxidation in the reaction mixture containing Fe^3+^. The reaction system consisted of 0.06 mM AsA (Sigma-Aldrich, USA), 10 μM CuSO_4_, and the master reaction mix (80 μL FRASC buffer, 10 μL AsA probe, and 10 μL iron [Fe^3+^] chloride solution). The addition of recombinant purified SKS18-His protein (113 kD) to the sample wells initiated the reaction. At different time points, the reaction mixture was dispensed into a cuvette and its absorbance measured using a spectrophotometer (Eppendorf). Reactions with ascorbic acid oxidase (Sigma-Aldrich, USA) were used as a positive control. For negative controls, an equivalent volume of purified protein from *E. coli* cells harboring the empty pCold TF DNA vector was added.

### Electrophoretic mobility shift assay

The 5′ biotin-labeled probes used in this study were synthesized by Generay (Shanghai). EMSA was performed using a Lightshift Chemiluminescent EMSA Kit (Thermo Scientific, USA). Recombinant purified MBP-TDF1 protein was incubated with biotin-labeled probes in binding buffer (10 mM Tris-HCl, pH 7.5, 50 mM KCl, 1 mM dithiothreitol). Competition experiments were performed by adding unlabeled DNA probes or labeled mutated probes. Each 20-μL binding reaction was incubated at room temperature for 20 min before separation on a 6% polyacrylamide gel in 0.5 × Tris-borate EDTA (TBE) buffer at 100 V for 90 min, after a pre-electrophoresis of 60 min. Separated samples were transferred onto a nylon membrane at 380 mA for 30 min. After crosslinking under UV light for 2 min, the subsequent experiments were conducted according to the manufacturer's protocol. The signals from the probes were captured with a Tanon-5500 Chemiluminescent Imaging System (Tanon, China). Primer sequences are listed in Supplemental Data Set S1.

### Transient transcription dual-luciferase assay

The promoter fragment of *SKS18* (1,391 bp) was PCR amplified from Col-0 and inserted into the pGreenII-0800-LUC vector. The resulting reporter construct contains 2 cassettes—the target promoter driving a firefly luciferase (*LUC*) reporter gene and a CaMV 35S promoter driving the *Renilla* luciferase (*REN*) gene as an internal control. To generate the effector constructs *35S:TDF1* and *35S:AMS*, the *TDF1* and *AMS* coding sequences were amplified and cloned into pCAMBIA1300*-35S* vector. Rosette leaves from Arabidopsis plants grown for 28 d were harvested and digested in an enzyme solution consisting of 0.8 M mannitol, 1 M KCl, 0.2 M MES (pH 5.7), 1.5% (w/v) cellulase R10, and 0.4% (w/v) macerozyme R10 (Yakult Honsha, Tokyo). After sequencing, the above plasmids were transfected into mesophyll cell protoplasts via polyethylene glycol and incubated at room temperature for 16 h in the light. Following lysis, the supernatants were extracted from the transfected protoplasts. The firefly and Renilla luciferase activities were quantified using a Dual-Luciferase Assay Kit (Promega, USA) and detected with a Synergy 2 multimode microplate (Bio-Tek) according to the manufacturer's instructions. The ratio of LUC/REN activities is shown. Primer sequences are listed in Supplemental Data Set S1.

### Accession numbers

Sequence data from this article can be found in TAIR under the following accession numbers: *TDF1* (At3g28470), *SKS18* (At1g75790), *VTC1* (At2g39770), *KEULE* (At1g12360), *DYT1* (At4g21330), *AMS* (At2g16910), *APX1* (At1g07890), *CAT3* (At1g20620).

## Supplemental data

The following materials are available in the online version of this article.

**Supplemental Figure S1**. Extra tapetal cells in *tdf1*, *tdf1 SKS18-OE #4*, and *sks18-1* anthers.

**Supplemental Figure S2**. Localization of KEULE-GFP in *KEULEpro:KEULE-GFP* transgenic plants.

**Supplemental Figure S3**. Expression analysis of *SKS18* and identification of the *sks18-2* mutant.

**Supplemental Figure S4**. *SKS18* transcript and SKS18 protein accumulation patterns in *tdf1* and *ams* mutants.

**Supplemental Figure S5**. TDF1 is a nucleus-localized protein in tapetal cells.

**Supplemental Figure S6**. *sks18*-*2* exhibits extra tapetal cells in anthers.

**Supplemental Figure S7**. Overexpression of *SKS18* partially rescues the defective tapetum seen in *tdf1*.

**Supplemental Figure S8**. Pollen wall material biosynthesis in *tdf1 SKS18-OE* transgenic plants.

**Supplemental Figure S9**. SDS-PAGE analysis of recombinant SKS18 protein.

**Supplemental Figure S10**. AsA content measurements.

**Supplemental Figure S11**. Extra tapetal cells observed in the *VTC1-OE* transgenic plants.

**Supplemental Figure S12**. Expression analysis of *VTC1* in the *tdf1* mutant.

**Supplemental Figure S13**. Analyses of $O_{2}^{-}$ in tapetum from wild-type and *tdf1* anthers.

**Supplemental Figure S14**. *SKS18-OE* transgenic plants have fewer tapetal cells.

**Supplemental Figure S15**. Multiple protein sequence alignment of SKS family proteins in Arabidopsis.

**Supplemental Data Set S1**. Primers used in this study.

**Supplemental Data Set S2**. Summary of statistical analyses.

**Supplemental Data Set S3**. ROS fluorescent images.

**Supplemental Data Set S4**. Multiple protein sequence of SKS family in FASTA format.

## Supplementary Material

koad037_Supplementary_Data
