# Supplementary material for: Ascorbic acid-mediated reactive oxygen species homeostasis modulates the switch from tapetal cell division to cell differentiation in Arabidopsis
Source: Plant Cell. 2023 Feb 14;35(5):1474–95. doi: 10.1093/plcell/koad037 (PMC10118275; doi:10.1093/plcell/koad037)
Supplement: koad037_Supplementary_Data [file koad037_supplementary_data.zip › TPC2022RA00796 Supplemental Data Set S4.pdf]

Multiple protein sequence of SKS family in FASTA format

```
>SKS5
-----------MAGSASFAAALFIGLSLLFAVTAEDPYRFFEWNITYGDIYPLGVRQQGI
LINGAFPGPDIHSVTNDNLIINVYNSLDEPFLLSWNGIQQRRNSFVDGVYGTTCPIPPGK
NYTYILQMKDQIGSFYYFPSLGFHKAAGGFGGIRILSRPRIPVPFPDPAGDTTVLIGDWY
K-ANHTDLRAQLDNGKKLP-LPDGILINGR-----------SSG--ATLNVEQGKTYRFR
ISNVGLQDSLNFRIQDHKMKVVEVEGTHTLQTTFSSLDVHVGQSYSVLVTADQTPR----
DYYVVVSSRFTSN----VLTTTGIFRYSNSAGGVSGPIP-----GGPTIQIDWSLNQARA
IRTNLSASGPRPNPQGSYHYGMINTTRTIRLAS-SAGQVDGKQRYAVNSVSFKPADTPLK
IADYFKIDG-VYRSGSIQYQP--TGG-GIYLDTSVMQVDYRTFVEIIFENSEDIVQSWHL
DGYSFWVVGMDGGQWSPDSRNEYNLRDAVARCTVQVYPSSWTAILIALDNVGMWNLRSEF
WARQYLGQQLYLRVYT------PSTSL-RDEYPIPKNALLCGRASGRSTRPL--------
------------------------------------------------------------
-
>SKS6
----------MMAVGRSGGTILLFCLSFFAAVTAESPYRFFDWNVTYGDIYPLGVRQQGI
LINGQFPGPDIHSVTNDNLIINVHNSLDEPFLISWNGVQNRRNSYVDGMYGTTCPIPPRS
NYTYILQVKDQIGSFYYFPSLAFHKAAGGFGGIRILSRPGIPVPFADPAGDYTVLIGDWY
K-FNHTDLKSRLDRGRKLP-SPDGILINGR-----------SNG--ATLNVEQGKTYRLR
ISNVGLQDSLNFRIQNHRMKLVEVEGTHTLQTMFSSLDVHVGQSYSVLITADQSPR----
DYYVVVSSRFTDK----IITTTGVLRYSGSSTPASGPIP-----GGPTIQVDWSLNQARA
IRTNLTASGPRPNPQGSYHYGLIPLIRTIVFGS-SAGQINGKQRYGVNSVSFVPADTPLK
LADFFKISG-VYKINSISDKP--TYG-GLYLDTSVLQVDYRTFIEIVFENQEDIVQSYHL
NGYSFWVVGMDGGQWKTGSRNGYNLRDAVSRSTVQVYPKSWTAIYIALDNVGMWNLRSEF
WARQYLGQQLYLRVFT------SSTSL-RDEYPIPKNSRLCGRARGRHTRPL--------
------------------------------------------------------------
-
>SKS7
----------MKVKSMNTRAMITTLLFLISLAFAEDPYRFFEWHVTYGNISPLGVAQQGI
LINGKFPGPDIISITNDNLIINVFNHLDEPFLLSWNGIRNWKNSFQDGVYGTMCPIPPGK
NYTYALQVKDQIGSFYYFPSLGFHKAAGGFGGIRISSRALIPVPFPTPADDYTLLVGDWY
K-TNHKDLKAQLDNGGKLP-LPDGILINGR-----------SSG--ATLNIEPGKTYRLR
ISNVGLQNSLNFRIQNHTMKLVEVEGRYTIQNLFSSLDVHVGQSYSVLITADQPAK----
DYYVVVSSRFTSK----ILTTTGVLHYSNSVAPVSGPIP-----DGP-IKLSWSFNQARA
IRTNLTASGPRPNPQGSYRYGVINITRTIRLAN-NLGHIEGKQRYAVNSASFYPADTPLK
LVDYFKIDG-VYKPGSISDQP--TNG-AIFPTTSVMQADFRAFVEVIFENSEDIVQSWHL
DGYSFYVVGMELGKWSPASRKVYNLNDAILRCTIQVYPRSWTAIYIALDNVGMWNMRSEI
WERQYLGQQFYMRVYT------TSTSL-RDEYLIPKNALLCGRASSSHR-----------
------------------------------------------------------------
-
```

>SKS8
----------MEVKSVNTTAMILGLFFLISFVAAEDPYKFFEWHVTYGNISPLKVAQQGI
LINGKFPGPDIAAVTNDNLIINVFNHLDEPFLISWSGIRNWRNSYQDGVYGTTCPIPPGK
NYTYALQVKDQIGSFYYFPSLGFHKAAGGFGAIRISSRPRIPVPFPAPAGDYTVLIGDWY
K-TNHKDLRAQLDNGGKLP-FPDGILINGR-----------GSG--ATLNIEPGKTYRLR
ISNVGLQNSLNFRIQNHKMKLVEVEGTHTIQTPFSSLDVHVGQSYSVLITADQPAK----
DYYIVVSSRFTSK----ILITAGVLHYSNSAGPVSGPIP-----EAP-IQLRWSFDQARA
IKTNLAASGPRPNPQGTYHYGKIKVTRTIKLAS-SAGNINGKQRYAVNSASFYPTDTPLK
LADYFKIAG-VYNPGSIPDQP--THG-AIYPVTSVMQTDYKAFVEIVFENWEDIVQTWHL
DGYSFFVVGMELGKWSAASRKVYNLNDAVSRCTVQVYPRSWTAIYVSLDNVGMWNLRSEL
WERQYLGQQFYMRVYT------PSTSL-RDEYLIPKNALLCGRATGHHTTTPGPLSEGSE
RF--
-
>SKS4
-------------MRGSCKVSIVLLLVLINGVLGDNPYRFFTWKITYGDIYPLGVKQQGI
LINGQFPGPHIDAITNDNIIISVFNYLKEPFLISWNGVQQRKNSWQDGVVGTTCPIPPGK
NFTYVIQVKDQIGSFYYFPSLAFHKAAGAFGAIRVWSRPRIPVPFSPPDGDFWLLAGDWY
K-TNHYVLRRLLEAGRNLP-NPDGVLINGR-----------GWGG-NTFTVQPGKTYRFR
ISNVGVATSLNFRIQGHTMKLVEVEGSHTVQNIYTSLDIHLGQSYSVLVTANQAPQ----
DYYIVISSRFTRK----VLTTTSILHYSNSRKGVSGPVP-----NGPTLDIASSLYQART
IRRNLTASGPRPNPQGSYHYGLIKPGRTIILAN-SAPWINGKQRYAVNGASFVAPDTPLK
LADYFKIPG-VFNLGSIPTSP--SGGNGGYLQSSVMAANFREFIEVVFQNWENSVQSWHV
SGYSFFVVGMDGGQWTPGSRAKYNLRDAVSRSTVQVYPRAWTAIYIALDNVGMWNIRSEN
WARQYLGQQFYLRVYT------SSTSY-RDEYPPPKNALMCGRAKGRHTRPF--------

-
>SKS9
---------MCWWLNGAVWTMMMMTISIISFVQADDPYRFFDWRVTYGNISPLGIPQRGI
LINGQYPGPDIYSVTNDNLIINVHNDLDEPFLLSWNGVQLRKNSYQDGVYGTTCPIPPGK
NYTYAIQVKDQIGSFFYFPSLAVHKAAGGFGGFRILSRPRIPVPFPEPAGDFTFLIGDWF
K-HDHKVLKAILDRGHKLP-LPQGVLINGQ-----------GVSYMSSITVHKGKTYRFR
ISNVGLQHTLNFRIQGHQMKLVEVEGTHTVQSMYTSLDIHVGQSYSVLVTMDQPDQ----
DYDIVVSTKFVAK----KLLVSSTIHYSNSRHSHSSSANSV-HVQQPADELDWSIKQARS
IRTNLTASGPRPNPQGSYHYGRIKISRTLILES-SAALVKRKQRYAINGVSFVPGDTPLK
LADYFKIKG-VFKMGSIPDKP--RRGRGMRMETSVMGAHHRDFLEIIFQNREKIVQSYHL
DGYSFWVVGTDRGTWSKASRREYNLRDAISRSTTQVYPESWTAVYVALDNVGMWNLRSEY
WARQYLGQQFYLRVYS------PTHSL-RDEYLLPKNALLCGRASNKHT---TP------

-
>SKS10
---------MEWWLNGGVW-MMMMTTTIISFVKAEDTL-FYNWRVTYGKIALDTLPRRGI

LINGQFPGPEIRSLTNDNLVINVQNDLDDPFLLSWNGVHMRKNSYQDGVYGTNCPIPPGK
NYTYDFQVKDQVGSYFYFPSLAVQKAAGGYGSLRIYSLPRIPVPFPEPAEDFTFLVNDWY
R-RNHTTLKKILDGGRKLPLMPDGVMINGQ-----------GVSTVYSITVDKGKTYRFR
VSNVGLQTSLNLEILGHQLKLIEVEGTHTVQTMYTSLDIHVGQTYSFLVTMDQPPQ----
NYSIVVSTRFINA----EVVIRATLHYSNSKGHKIITAR-----RPDPDDVEWSIKQAQS
IRTNLTASGPRTNPQGSYHYGKMKISRTLILES-SAALVKRKQRYAINGVSFVPSDTPLK
LADHFKIKD-VFKVGTIPDKP--RRGGGIRLDTAVMGAHHNAFLEIIFQNREKIVQSYHL
DGYNFWVVGINKGIWSRASRREYNLKDAISRSTTQVYPKSWTAVYVALDNVGMWNLRSQF
WARQYLGQQFYLRVHS------PNHSP-KDEYPLPKNALLCGRASNKNMSIITP------

-
>SKS11
----------MRG-VKLLAACLYLAAAATVVVRAEDPYFHHVWNVTYGTVSPLGVPQQVI
LINGQFPGPNVNSTSNNNVIINVFNNLDEPFLLTWNGIQHRKNCWQDGTPGTMCPIMPGT
NYTYHFQPKDQIGSYFYYPSTAMHRSAGGFGGLRVNSRLLIPVPYADPEDDYTVLIGDWY
T-KSHTQLKKFLDSGRTLG-RPDGILINGKS-------GKGDGSDAPLFTLKPGKTYRVR
ICNVGLKTSLNFRIQNHKLKLVEMEGSHVLQNDYDSLDVHVGQCYGTILTANQEAK----
DYYMVASSRFLKS----VITTTGLLRYEGGKGPASSQLP-----PGPVG-WAWSLNQFRS
FRWNLTASAARPNPQGSYHYGKINITRTIKLVN-TQGKVDGKLRYALNGVSHTDPETPLK
LAEYFGVADKVFKYDSITDNPTPEQIKSIKIVPNVLNITHRTFIEVVFENHEKSVQSWHL
DGYSFFAVAVEPGTWTPEKRKNYNLLDAVSRHTVQVYPKCWAAILLTFDNCGMWNVRSEN
SERRYLGQQLYASVLS------PEKSL-RDEYNMPETSLQCGLVKGTPKPNPYAGA----

-
>SKS12
----------MKGGVKLLAVCLCVATATVMMVQAEDPYFHHVWNVTYGTASPLGVPQQVI
LINGQFPGPNINSTSNNNVIVNVFNNLDEPFLITWAGIQHRKNCWQDGTAGTMCPIPPGQ
NFTYHFQPKDQIGSYFYYPTTAMHRAAGGFGGLRVNSRLLIPVPYADPEDDYTILINDWY
T-KSHTQLKKFLDSGRTIG-RPDGILINGKS-------GKTDGSDKPLFTLKPGKTYRVR
ICNVGLKASLNFRIQNHKMKLVEMEGSHVLQNDYDSLDVHVGQCFGVIVTADQEPK----
DYYMIASTRFLKK----PLTTTGLLRYEGGKGPASSQLP-----AAPVG-WAWSLNQYRS
FRWNLTASAARPNPQGSYHYGKINITRTIKLVN-TQGKVDGKLRYALSGVSHTDPETPLK
LAEYFGVADKVFKYDTISDNPNPDQIKNIKIEPNVLNITHRTFIEVVFENHERSVQSWHL
DGYSFFAVAVEPGTWTPEKRKNYNLLDAVSRHTVQVYPKCWAAILLTFDNCGMWNIRSEN
AERRYLGQQLYASVLS------PEKSL-RDEYNMPETSLQCGLVKGKPKVNPYAGA----

-
>SKS13
-----------MQGGRLLTVLVCLAS-TVALVSAGDPYFYYTWNVTYGTAAPLGIPQQVI
LINGQFPGPNLNSTSNNNVVINVFNNLDEPFLLTWSGLQHRKNSWQDGVTGTSCPIPAGT
NFTYHFQPKDQIGSYFYYPSTALHRFAGGFGGLRVNSRLLIPVPYADPEDDRTILINDWY

A-KSHTALKNFLDSGRTLG-SPDGVLINGKS-------GKLGGNNAPLFTMKPGKTYKYR
ICNVGFKSTLNFRIQGHKMKLVEMEGSHVLQNDYDSLDVHVGQCFAVLVTADQVAK----
NYYMVASTRFLKK----EVSTVGVMSYEGSNVQASSDIP-----KAPVG-WAWSLNQFRS
FRWNLTASAARPNPQGSYHYGKINITRTIKLAN-TKNLVNGKVRFGFNGVSHVDTETPLK
LAEYFGMSEKVFKYNVIKDEP-AAKITTLTVEPNVLNITFRTFVEVVFENHEKSMQSFHL
DGYSFFAVASEPGRWTPEKRNNYNLLDAVSRHTVQVYPKSWSAILLTFDNAGMWNIRSEN
WERRYLGQQLYVSVLS------PEKSL-RDEYNIPLNTNLCGIVKGLPLPTPYTI-----

-
>SKS14
-----------MEG-RLLTVLVCLVS-TVAIVNAGDPYFFHTWNVTYGTASPLGVPQKVI
LINGQFPGPNLNSTSNNNVVINVFNHLDEPFLLTWSGIQHRKNCWQDGVAGTSCPIPAGQ
NFTYHFQPKDQIGSYFYYPTTSLHRFAGGFGGLRVNSRLLIPVPYADPEDDYTVLLGDWY
T-AGHTALKNFLDSGRTLG-LPNGVLINGKS-------GKVGGKNEPLFTMKPGKTYKYR
LCNVGFKSTLNFRIQNHKMKLVEMEGSHVIQNDYDSLDVHVGQCFSVLVTANQAAK----
DYYMVASTRFLKK----ELSTVGVIRYEGSNVQASTELP-----KAPVG-WAWSLNQFRS
FRWNLTSNAARPNPQGSYHYGKINITRSIKLVN-SKSVVDGKVRFGFNGVSHVDTETPLK
LAEYFQMSEKVFKYNVIKDEP-AAKITALTVQPNVLNITFRTFVEIIFENHEKTMQSFHL
DGYSFFAVASEPGRWTPEKRENYNLLDAVSRHTVQVYPKSWSAILLTFDNAGMWNIRSEN
LERKYLGEQLYVSVLS------PEKSL-RDEYNIPLNTNLCGIVKGLPLPAHYS------

-
>SKS15
-----MKQTNLLVCKLFIGALFWLG---SVLVNAEDPYMFYTWTVTYGTRSPLGVPQQVI
LINGQFPGPAIEAVTNNNIVVNLINKLDEPFLITWNGVKQRRTSWQDGVLGTNCPIQPNS
NWTYQFQLKDQIGTYTYFASTSLHRASGAFGALNINQRSVITTPYPTPDGDFTLLVSDWF
SNMTHKDLRKSLDAGSALP-LPDALLING-------------VSKGLIFTGQQGKTYKFR
VSNVGIATSINFRIQNHTMSLIEVEGAHTLQESYESLDVHVGQSMTVLVTLKASVR----
DYFIVASTRFTKP----VLTTTASLRYQGSKNAAYGPLP-----IGPTYHIHWSMKQART
IRMNLTANAARPNPQGSFHYGTIPINRTLVLAN-AATLIYGKLRYTVNRISYINPTTPLK
LADWYNISG-VFDFKTIISTP---TTGPAHIGTSVIDVELHEFVEIVFQNDERSIQSWHM
DGTSAYAVGYGSGTWNVTMRKRYNLVDAVPRHTFQVYPLSWTTILVSLDNKGMWNLRSQI
WSRRYLGQELYVRVWN------DEKSL-YTEAEPPLNVLYCGKAKRPL------------

-
>SKS16
-----MKQKHLLLLGFLL-AYCFSS---VFVINAEDPYLFFTWTVTYGTRSPLGVPQQVI
LINGQFPGPPIEGVTNNNIVVNVINKLDEPFLITWNGIKQRKMSWQDGVLGTNCPIQPKS
SWTYHFQLKDQIGTYAYFASTSMHRASGAFGALNVNQRSVIFVPYPKPDADFTLLVSDWY
K-MGHKELQRRLDSSRALP-PPDGLLING-------------ASKGLVFTGQHGKIYRFR
ISNVGISTSINFRIQGHMMTLVEVEGSHTLQEVYESLDIHVGQSVTVLVTLKAPVK----

DYFIVASTRFTKP----ILTTTGILSYQGSKIRPSHPLP-----IGPTYHIHWSMKQART
IRLNLTANAARPNPQGSFHYGTIPINRTFVLAN-GRAMINGKLRYTVNRVSYVNPATPLK
LADWFNIPG-VFNFKTIMNIP---TPGPSILGTSVFDVALHEYVEFVFQNNEGSIQSWHL
DGTSAYVVGYGSGTWNMAKRRGYNLVDAVSRHTFQVYPMSWTSILVSLDNKGMWNLRSQI
WSRRYLGQELYVRVWN------NEKSL-YTESEPPVNVLFCGKAKHPRLI----------

-
>SKS17
MKMASRKTTSLLNHLLLLGALTLLSS--LVIVKGESPYKFYTWTVTYGIISPLGVPQQVI
LINGQFPGPKLEVVTNDNIILNLINKLDQPFLLTWNGIKQRKNSWQDGVLGTNCPIQPNS
NFTYKFQTKDQIGTFNYFPSTAFHKAAGGFGAINVYARPGIPIPYPLPTADFTLLVGDWF
K-TNHKTLQQRLDSGGVLP-FPDGMLING-------------QTQ-STFSGDQGKTYMLR
ISNVGLSSTFNFRIQGHTMKVVEVEGSHVIQTDYDSLDIHVGQSLAVLVTLNQSPK----
DYYIVASTRFIRS----KLSVMGLLRYSNSRVPASGDPP-----ALPPGELVWSMRQART
FRWNLTANAARPNPQGSFHYGMISPTKTFVFSN-SAPLINGKQRYAVNGVSYVKSETPLK
LADHFGISG-VFSTNAIQSVP---SNSPPTVATSVVQTSHHDFLEIVFQNNEKSMQSWHL
DGYDFWVVGFGSGQWTPAKRSLHNLVDALTRHTTQVYPESWTTILVSLDNQGMWNMRSAI
WERQYSGQQFYLKVWN------SVQSL-ANEYNPPDNLQLCGKAVGRHV-----------

-
>SKS1
----------MAATCSLLASFLLCFALLSAVSFAADPFVSYDFRVSYLTASPLGVPQQVI
AVNGQFPGPLLNATTNYNVVVNVFNHLDEPLLLTWPGIQMRRNSWQDGVLGTNCPIPPRW
NFTYQFQVKDQIGSFFYSPSLNFQRASGGFGPIVINNRDIIPIPFPQPDGELIFIIGDWY
T-QDHKALRRALDSGKELG-MPDGVLINGKGPYKYN-SSVPDGIDYLTFHVEPGKTYRIR
VHNVGISTSLNFRIQNHSLLLVETEGHYTSQANFTDFDVHVGQSYSFLVTMDQDATS---
DYYIVASARFVNETVWQRVTGVAILHYSNSKGPVSGPLPV---PKTDVSSPWSAMSQPKT
IRQNTSASGARPNPQGSFHYGQINITNTYILRSLPPTIINGALRATLNGISFVNPSTPVR
LADRNKVKG-AYKLD-FPDRP--FNRPL-RLDRSMINATYKGFIQVVFQNNDTKIQSFHV
DGYSFFVVGMDFGIWSEDKKGSYNNWDAISRSTIEVYPGGWTAVLISLDNVGVWNIRVEN
LDRWYLGEETYMRITN------PEEDG-KTEMDPPDNVLYCGALKNLQKEQ----HHSAA
T-SILNGHLKLML--LMVLLASVFRFC--------------------------------
-
>SKS2
----------MAAT-DFFFAFVFSFALIFGFSFAGDPYVSYDFTLSYITASPLGVPQQVI
AVNGKFPGPVINATTNYNVHVNVLNHLDEPLLLTWPGVQMRRNSWQDGVLGTNCPIPPNW
NFTYDFQLKDQIGSYFYSPSLNFQRASGGFGALIINNRDLVPIPFTEPDGEIIFIIGDWY
T-QNHTALRRILDSGKELG-MPDGVLINGKGPFKYN-SSVPDGIEHETVNVDPGKTYRIR
VHNVGISTSLNFRIQNHKLLLIETEGRYTSQMNFTDFDVHVGQSYSFLVTMDQNATS---
DYYIVASARFVNETVWQRVTGVGILHYSNSKGPASGPLPV---SATDVNHPWSAMNQPRA
IKQNTSASGARPNPQGSFHYGQINITRTYILRSLPPTKINGKLRATLNGISFVNPSTPMR

LADDHKVKG-DYMLD-FPDRP--LDEKLPRLSSSIINATYKGFIQVIFQNNDTKIQSFHI
DGYAFYVVAMDFGIWSEDRNSSYNNWDAVARSTVEVYPGAWTAVLISLDNVGVWNIRVEN
LDRWYLGQETYMRIIN------PEENG-STEMDPPENVMYCGALQAMQKEQ----HHSSA
TKSMTNGQLILIFSMMMVLLSSFSSFC--------------------------------
-
>SKU5
-------------MDLFKILLLVFFVNISFCFAADPYSFYNFEVSYITASPLGVPQQVI
AINGKFPGPTINVTTNENLVVNVRNKLDEGLLLHWNGIQQRRVSWQDGVLGTNCPIPPKW
NWTYEFQVKDQIGSFFYFPSLHFQRASGGFGSFVVNPRAIIPVPFSTPDGDITVTIGDWY
I-RNHTALRKALDDGKDLG-MPDGVLINGKGPYRYNDTLVADGIDFETITVHPGKTYRLR
VSNVGISTSLNFRIQGHNLVLAESEGSYTVQQNYTSLDIHVGQSYSFLVTMDQNASS---
DYYIVASARVVNETIWRRVTGVGILKYTNSKGKAKGQLPP--GPQDEFDKTFS-MNQARS
IRWNVSASGARPNPQGSFKYGSINVTDVYVLRNMPPVTISGKRRTTLNGISFKNPSTPIR
LADKLKVKD-VYKLD-FPKRP--LTGPA-KVATSIINGTYRGFMEVVLQNNDTKMQSYHM
SGYAFFVVGMDYGEWTENSRGTYNKWDGIARSTIQVYPGAWSAILISLDNPGAWNLRTEN
LDSWYLGQETYVRVVN------PDENN-KTEFGHPDNVLYCGALSKLQKPQ----KVSSS
A-SKSIGFTSLSMVVMALVMMMMLQH---------------------------------
-
>SKS3
----------MRCFPPPLWCTSLVVFLSVTGALAADPYVFFDWTVSYLSASPLGTRQQVI
GINGQFPGPILNVTTNWNVVMNVKNNLDEPLLLTWNGIQHRKNSWQDGVLGTNCPIPSGW
NWTYEFQVKDQIGSFFYFPSTNFQRASGGYGGIIVNNRAIIPVPFALPDGDVTLFISDWY
T-KSHKKLRKDVESKNGLR-PPDGIVINGFGPFASN------GSPFGTINVEPGRTYRFR
VHNSGIATSLNFRIQNHNLLLVETEGSYTIQQNYTNMDIHVGQSFSFLVTMDQSGSN---
DYYIVASPRFATS---IKASGVAVLRYSNSQGPASGPLP---DPPIELDTFFS-MNQARS
LRLNLSSGAARPNPQGSFKYGQITVTDVYVIVNRPPEMIEGRLRATLNGISYLPPATPLK
LAQQYNISG-VYKLD-FPKRP--MNRHP-RVDTSVINGTFKGFVEIIFQNSDTTVKSYHL
DGYAFFVVGMDFGLWTENSRSTYNKGDAVARSTTQVFPGAWTAVLVSLDNAGMWNLRIDN
LASWYLGQELYLSVVN------PEIDIDSSENSVPKNSIYCGRLSPLQKDQAQRVNFSGS
QRSIFVTSRGILLALFAILVN-INRLCNTKKILDSTAKAQTDLSLVETDRISEKNRDRIS
Y

>SKS18
------------MRHVFVEVLVLISLVILELSYAFAPISSYQWVVSYSQRFILGGNKQVI
VINDMFPGPILNATANDIIVVNIFNNLPEPFLMTWNGLQLRKNSWQDGVRGTNCPILPGT
NWTYRFQVKDQIGSYFYFPTLLLQKAAGGYGAIRIYPPELVPVPFPKPDEEYDILIGDWF
Y-LDHTVMRASLDAGHSLP-NPDGILFNGR------------GPEETFFAFEPGKTYRLR
ISNVGLKTCLNFRIQDHDMLLVETEGTYVQKRVYSSLDIHVGQSYSILVTAKTDPVGIYR
SYYIFATARFTDS----YLGGIALIRYPGSPLDPVGQGP----LAPALQDFGSSVEQALS
IRMDLNVGAARSNPQGSYHYGRINVTRTIILHN-DVMLSSGKLRYTINGVSFVYPETPLK
LVDHFQLND-TIIPGMFPVYP---SNKTPTLGTSVVDIHYKDFIHIVFQNPLFGLESYHI

DGYNFFVVGYGFGAWSESKKAGYNLVDAVSRSTVQVYPYSWTAILIAMDNQGMWNVRSQK
AEQWYLGQELYMRVKGEGEEDPSTIPV-RDENPIPGNVIRCGKVR---------------

-
