# Supplementary material for: Ascorbic acid-mediated reactive oxygen species homeostasis modulates the switch from tapetal cell division to cell differentiation in Arabidopsis
Source: Plant Cell. 2023 Feb 14;35(5):1474–95. doi: 10.1093/plcell/koad037 (PMC10118275; doi:10.1093/plcell/koad037)
Supplement: koad037_Supplementary_Data [file koad037_supplementary_data.zip › TPC2022RA00796R3_Supplemental Figures 115 20230131.pdf]

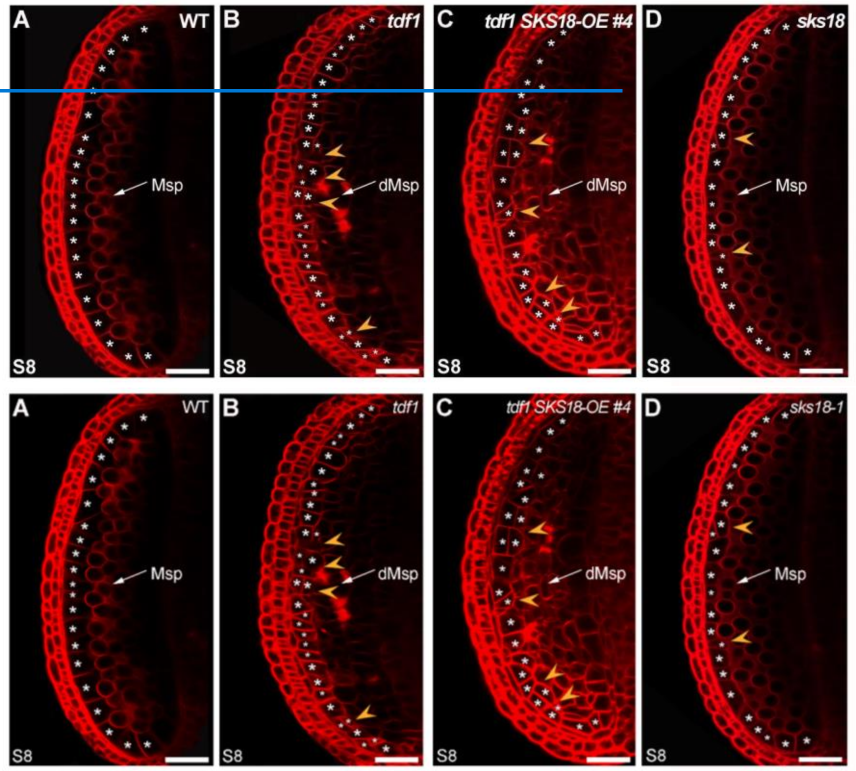

Supplemental Figure S1. Extra tapetal cells in *tdf1*, *tdf1 SKS18-OE #4* and *sks18-1* anthers (Supports Figure 1 and Figure 4).

(A-D) One abaxial locule in anther ~~is~~ was stained with FM4-64 in WT, *tdf1*, *tdf1 SKS18-OE #4* and *sks18-1* at stage 8. ~~The E~~Eextra tapetal cells (yellow arrowheads) are present in anthers from *tdf1*, *tdf1 SKS18-OE #4* and *sks18-1*. Msp, microspore; dMsp, defective microspore. Scale bars, 50 µm.

设置了格式: 英语(美国)
设置了格式: 英语(美国)
设置了格式: 英语(美国)
带格式的: 缩进: 左侧: -0.5 厘米, 右侧: -0.33 厘米
设置了格式: 英语(美国)
设置了格式: 英语(美国)
设置了格式: 英语(美国)
带格式的: 缩进: 左侧: -0.52 厘米, 右侧: -0.33 厘米
设置了格式: 英语(美国)
设置了格式: 英语(美国)

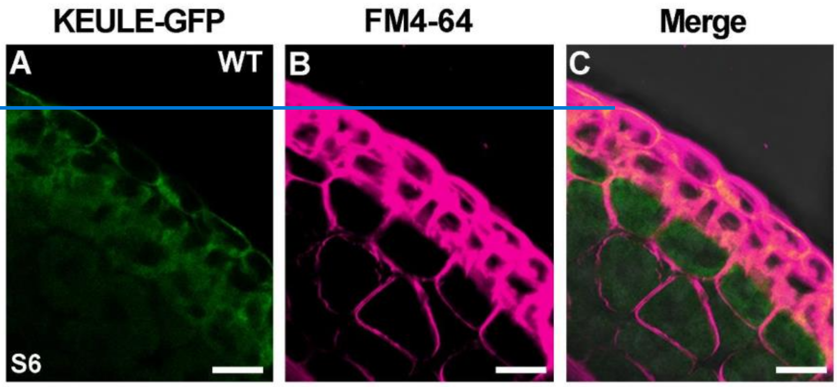

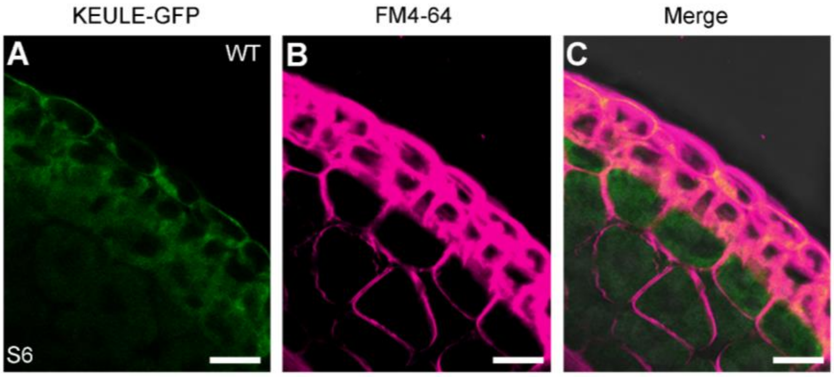

Supplemental Figure S2. Localization of KEULE-GFP ~~driven by *ProKEULE*~~in *KEULEpro:KEULE-GFP* transgenic plants (Supports Figure 1).
(A-C) ~~Expression~~ Fluorescence of KEULE-GFP from ~~*Pro*~~*KEULEpro:KEULE-GFP* anthers stained with FM4-64 in WT ~~anthers~~. KEULE-GFP shows a cytosolic localization in ~~the~~ non-dividing tapetal cells at stage 6. Scale bars, 20 μm.

设置了格式: 英语(美国)
设置了格式: 英语(美国)
设置了格式: 英语(美国)
带格式的: 缩进: 左侧: -0.5 厘米, 右侧: -0.24 厘米, 边框: 底端: (单实线, 自动设置, 0.5 磅 行宽)
带格式的: 缩进: 左侧: -0.5 厘米, 右侧: -0.33 厘米
设置了格式: 英语(美国)
设置了格式: 英语(美国)
设置了格式: 英语(美国)
设置了格式: 字体: 非倾斜
设置了格式: 英语(美国)
设置了格式: 英语(美国)
设置了格式: 英语(美国)
设置了格式: 英语(美国)
带格式的: 缩进: 左侧: -0.52 厘米, 右侧: -0.33 厘米
设置了格式: 英语(美国)
设置了格式: 英语(美国)

Supplemental Data. Wu, Hou et al. (2023). TDF1 promotes tapetal cell differentiation. Plant Cell.

~~Supplemental Data. Wu et al. (2023). Plant Cell.~~

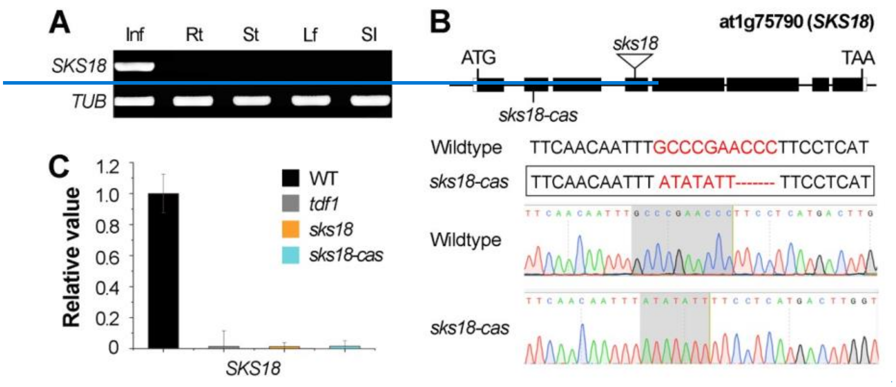

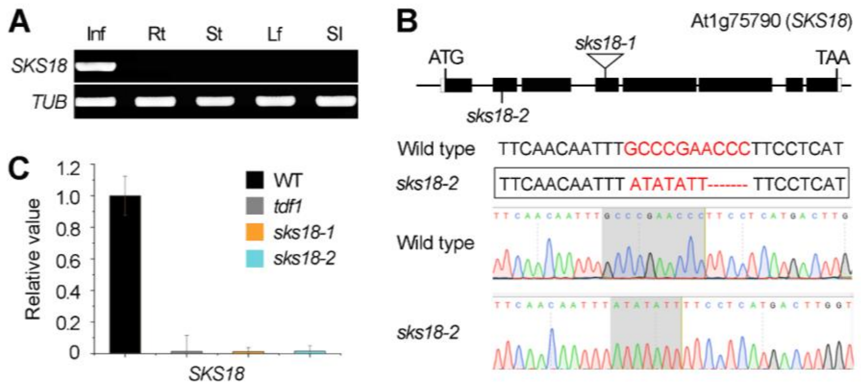

Supplemental Figure S3. Expression analys~~i~~es of *SKS18* and identification of the *sks18-2*~~*cas*~~ ~~allele~~ mutant (Supports Figure 2 and Figure 4).

(A) *SKS18* expression in various tissues using 28 cycles by RT-PCR analysis. Inf, inflorescence; Lf, leaf; Rt, root; St, stem; Sl, seedling. **(B)** Schematic ~~drawing~~ diagram of the ~~gene~~ *SKS18* locus indicating the mutated positions in the *sks18-1* and *sks18-*~~*cas*~~*2* mutants. The "-" shows the deleted bases. ~~The~~

CRISPR/~~-~~Cas9 T3 homozygous transgenic plants were used for phenotyp~~e~~ing ~~analysis~~. **(C)** *SKS18* expression ~~is detected~~ in inflorescences from WT, *tdf1*, *sks18-1* and *sks18-*~~*cas*~~*2* by ~~q~~RT-qPCR analysis. ~~Error bars represent the~~Data are means ± SD. Three biological repeats were performed, with similar results. Each biological replicate was performed with three technical replicates for ~~q~~RT-qPCR.

设置了格式: 英语(美国)

设置了格式: 英语(美国)

设置了格式: 英语(美国)

设置了格式: 英语(美国)

设置了格式: 英语(美国)

带格式的: 缩进: 左侧: -0.52 厘米, 右侧: -0.33 厘米

批注 **[PSC1]:** In this panel, Wildtype should be two words (Wild type)/

批注 **[楼2R1]:** I revised the words and used *sks18-2* instead of *sks18-cas*, thanks!

设置了格式: 英语(美国)

设置了格式: 英语(美国)

设置了格式: 英语(美国)

设置了格式: 英语(美国)

设置了格式: 英语(美国)

设置了格式: 英语(美国)

设置了格式: 英语(美国)

设置了格式: 英语(美国)

设置了格式: 英语(美国)

设置了格式: 英语(美国)

设置了格式: 英语(美国)

设置了格式: 英语(美国)

设置了格式: 英语(美国)

带格式的: 缩进: 左侧: -0.5 厘米, 右侧: -0.24 厘米, 边框: 底端: (单实线, 自动设置, 0.5 磅 行宽)

Supplemental Data. Wu, Hou et al. (2023). TDF1 promotes tapetal cell differentiation. Plant Cell.

Supplemental Data. Wu et al. (2023). Plant Cell.

设置了格式: 英语(美国)

设置了格式: 英语(美国)

设置了格式: 英语(美国)

带格式的: 缩进: 左侧: -0.5 厘米, 右侧: -0.33 厘米

设置了格式: 英语(美国)
设置了格式: 英语(美国)
设置了格式: 英语(美国)
带格式的: 缩进: 左侧: -0.5 厘米, 右侧: -0.24 厘米, 边框: 底端: (单实线, 自动设置, 0.5 磅 行宽)

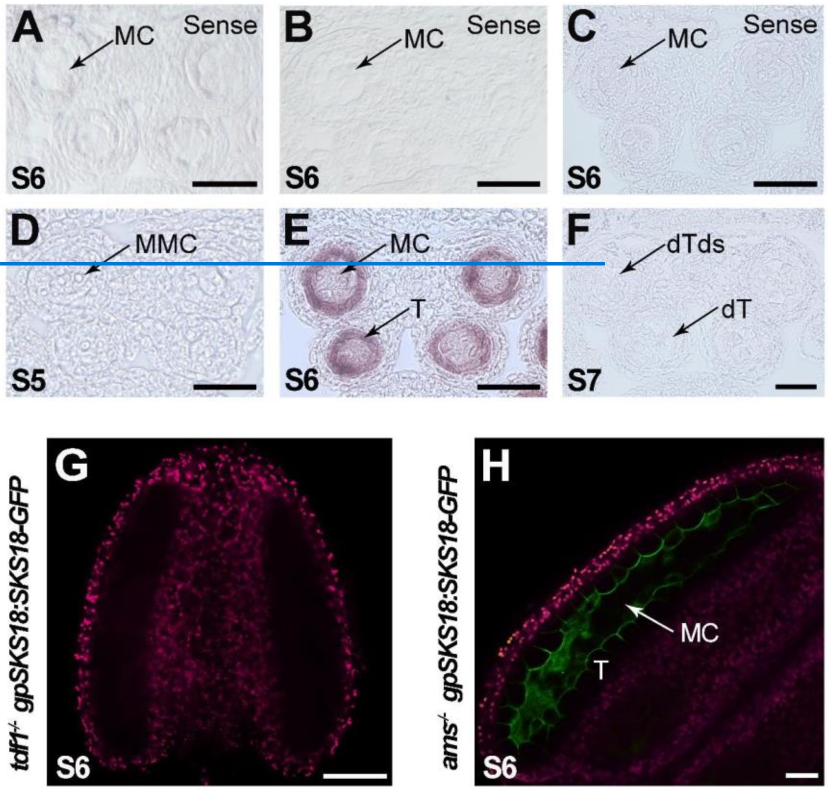

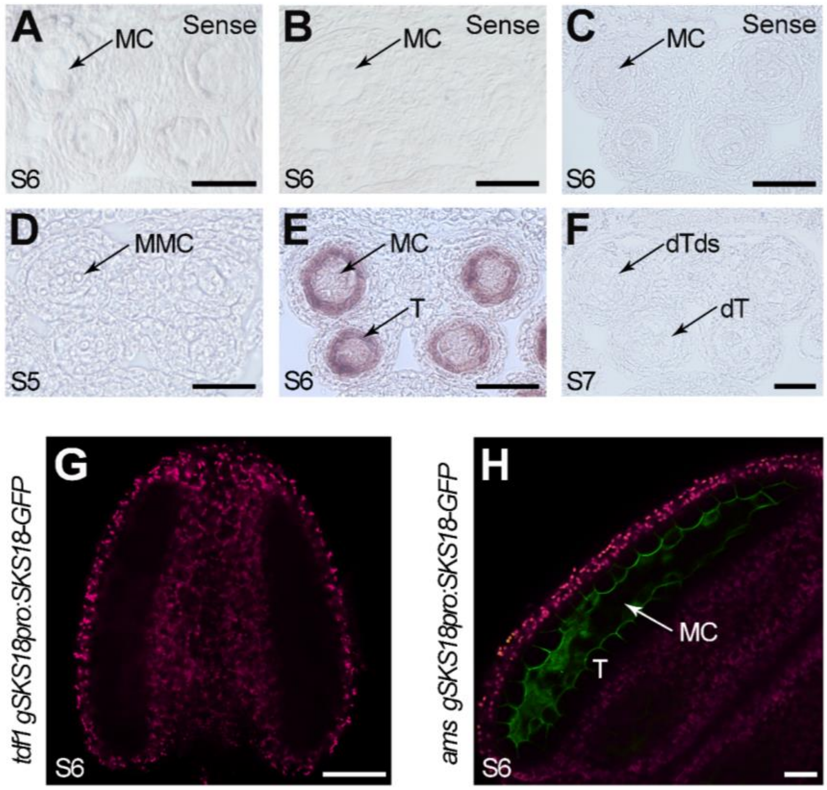

Supplemental Figure S4. *SKS18* transcript ~~gene~~ and SKS18 ~~protein expression~~accumulation patterns in ~~TF~~ *tdf1* and *ams*~~1~~ mutants (Supports Figure 2). RNA *in situ* hybridization of *SKS18* transcripts in anthers of WT **(A)** and *tdf1* **(B)** using a sense probe at stage 6. *SKS18* transcripts in *ams* anthers ~~of *ams*~~ using an antisense probe **(D-F)** and a sense probe **(C)**. Scale bars, 20 µm. *tdf1* **(G)** and *ams* **(H)** ~~expressing~~ harboring the ~~Pro~~*SKS18pro:SKS18-GFP* transgene. Chloroplast autofluorescence is shown in magenta. Scale bars, 20 µm. MMC, mother microspore cell; MC, meiocytes; T, tapetum; dT, defective tapetum; dTds, defective tetrads.

设置了格式: 英语(美国)
设置了格式: 英语(美国)
设置了格式: 英语(美国)
设置了格式: 英语(美国)
设置了格式: 字体: 非加粗
设置了格式: 字体: 非倾斜, 英语(美国)
设置了格式: 英语(美国)
设置了格式: 英语(美国)
设置了格式: 字体: 倾斜
设置了格式: 字体: 倾斜
设置了格式: 英语(美国)
设置了格式: 英语(美国)
带格式的: 缩进: 左侧: -0.52 厘米, 右侧: -0.33 厘米
设置了格式: 英语(美国)
批注 [PSC3]: The -/- should be deleted in all figures, since the lowercase names already convey the mutant status.
批注 [楼4R3]: I deleted the -/- in all figures, thanks.
设置了格式: 英语(美国)
设置了格式: 英语(美国)
设置了格式: 英语(美国)
设置了格式: 英语(美国)
设置了格式: 英语(美国)
设置了格式: 英语(美国)

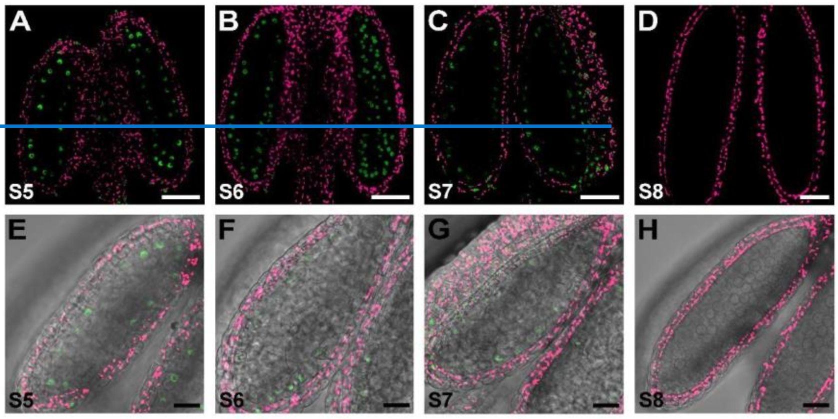

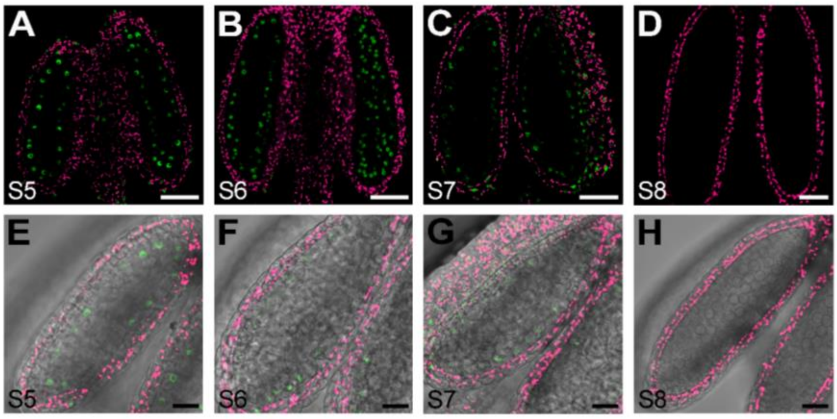

Supplemental Figure S5. TDF1 ~~shows~~ is a ~~nuclear~~nucleus- ~~localization~~ localized protein in tapetal cells (Supports Figure 3).

(A-D) Localization of VENUS-TDF1 ~~driven by~~in ~~*Pro*~~*TDF1pro:VENUS-TDF1* transgenic lines at stages 5-8. **(E-H)** Higher magnification view of VENUS-TDF1 location in anthers. Chloroplast autofluorescence is shown in magenta. Scale bars, 20 μm.

设置了格式: 英语(美国)
设置了格式: 英语(美国)
设置了格式: 英语(美国)
带格式的: 缩进: 左侧: -0.5 厘米, 右侧: -0.24 厘米, 边框: 底端: (单实线, 自动设置, 0.5 磅 行宽)

设置了格式: 英语(美国)
设置了格式: 英语(美国)
设置了格式: 英语(美国)
设置了格式: 英语(美国)
设置了格式: 英语(美国)
设置了格式: 字体: 倾斜, 英语(美国)
设置了格式: 英语(美国)
带格式的: 缩进: 左侧: -0.52 厘米, 右侧: -0.33 厘米
设置了格式: 英语(美国)
设置了格式: 英语(美国)

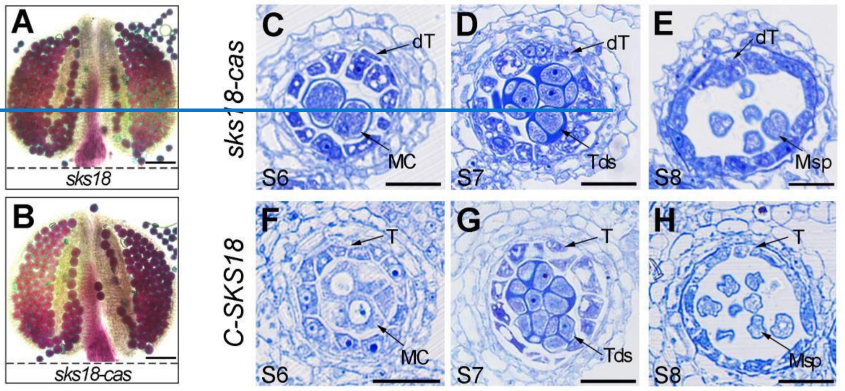

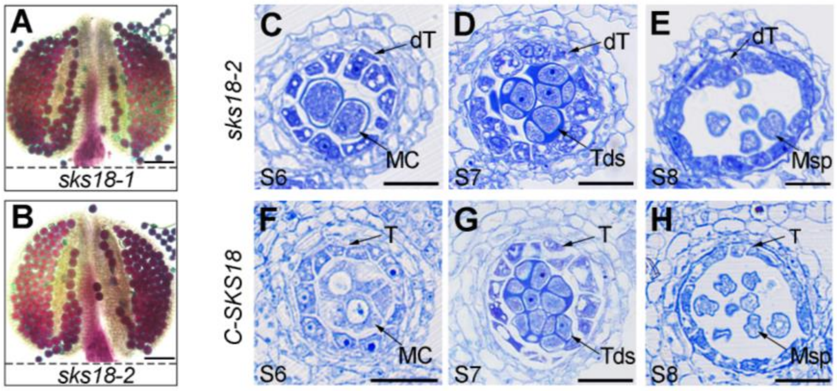

Supplemental Figure S6. *sks18-~~cas~~ 2* exhibits ~~the~~ extra tapetal cells in anthers (Supports Figure 4).

Alexander's staining of anthers from *sks18-1* **(A)** and *sks18-~~cas~~ 2* plants **(B)**. Scale bars, 20 μm. Semi-thin sections of anthers from *sks18-~~cas~~ 2* **(C-E)** and *C-SKS18* (the complementation~~ry~~ line of *SKS18* in *sks18*) **(F-H)** plants at indicated stages.
Scale bars, 20 μm. MC, meiocytes; Msp, microspore; T, tapetum; Tds, tetrads; dT, defective tapetum.

设置了格式: 英语(美国)
设置了格式: 英语(美国)
设置了格式: 英语(美国)
设置了格式: 英语(美国)
设置了格式: 英语(美国)
设置了格式: 英语(美国)
设置了格式: 英语(美国)
设置了格式: 英语(美国)
带格式的: 缩进: 左侧: -0.52 厘米, 右侧: -0.33 厘米
设置了格式: 英语(美国)
设置了格式: 英语(美国)
设置了格式: 英语(美国)
设置了格式: 英语(美国)

Supplemental Data. Wu, Hou et al. (2023). TDF1 promotes tapetal cell differentiation. Plant Cell.

设置了格式: 英语(美国)
设置了格式: 英语(美国)
设置了格式: 英语(美国)
带格式的: 缩进: 左侧: -0.5 厘米, 右侧: -0.24 厘米, 边框: 底端: (单实线, 自动设置, 0.5 磅 行宽)

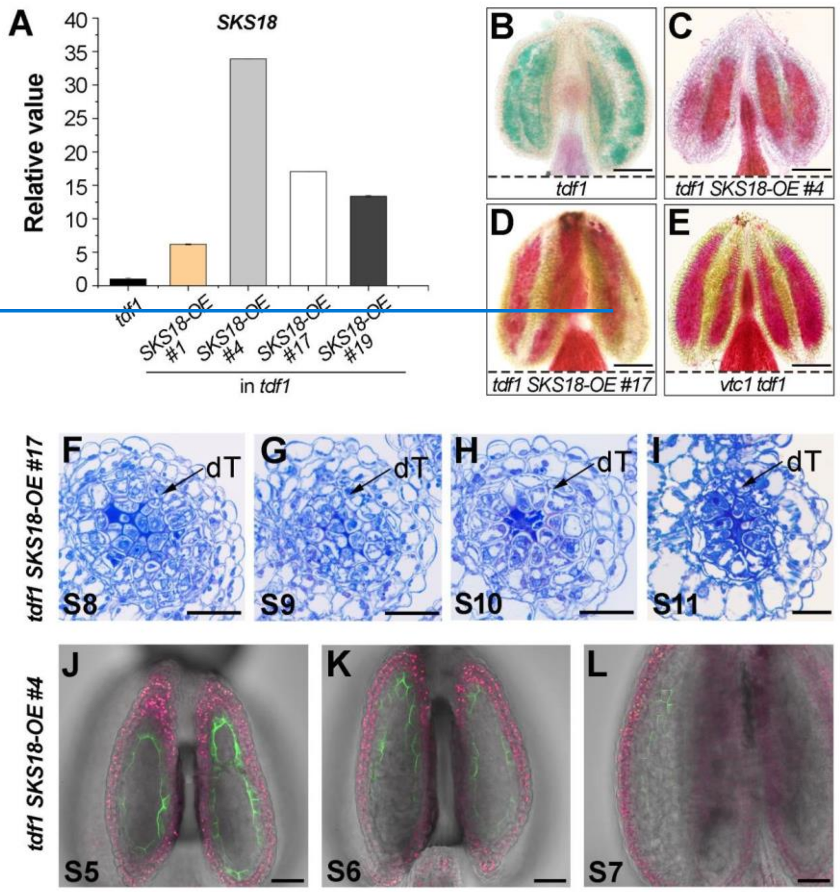

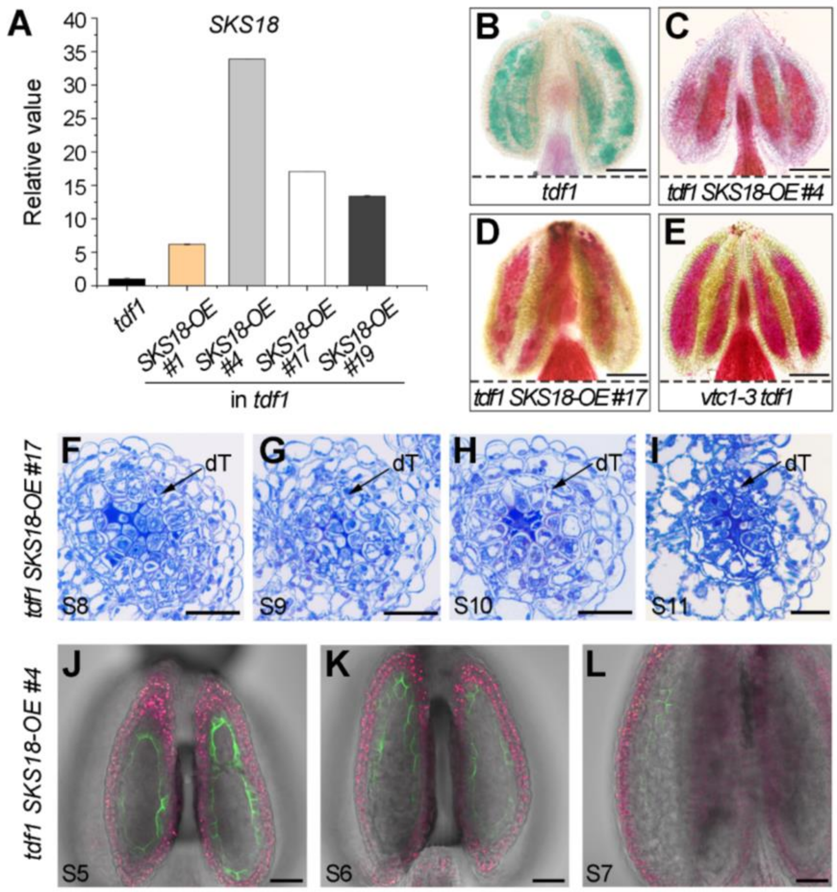

Supplemental Figure S7. Overexpression of *SKS18* partially rescues the defective tapetum ~~in~~ ~~of~~ seen in *tdf1* (Supports Figure 4).

(A) *SKS18* expression ~~is detected~~ in inflorescences from WT and different *tdf1 SKS18-OE* transgenic plants by ~~q~~RT-qPCR analysis. ~~Error bars represent the~~Data are means ± SD. Three biological repeats were performed, with similar results. Each biological replicate was performed with three technical replicates ~~for qRT-PCR~~. **(B-E)** Alexander's staining of anthers from *tdf1* **(B)**, *tdf1 SKS18-OE #4* **(C)**, *tdf1 SKS18-OE #17* **(D)** and *vtc1-3 tdf1* **(E)** plants. Scale bars, 20 μm. **(F-I)** Semi-thin sections of anthers from *tdf1 SKS18-OE #17* at the indicated stages. Scale bars, 20 μm. **(J-L)** SKS18-GFP ~~is~~ localizes~~d~~ to the periphery of tapetal cells in *tdf1 SKS18-OE #4* transgenic plants. Chloroplast autofluorescence is shown in magenta. Scale bars, 20 μm. dT, defective tapetum.

设置了格式: 英语(美国)
设置了格式: 英语(美国)
设置了格式: 英语(美国)
设置了格式: 英语(美国)
设置了格式: 英语(美国)
带格式的: 缩进: 左侧: -0.52 厘米, 右侧: -0.33 厘米
设置了格式: 英语(美国)
设置了格式: 英语(美国)
设置了格式: 字体: 加粗
设置了格式: 英语(美国)
设置了格式: 英语(美国)
设置了格式: 英语(美国)
设置了格式: 英语(美国)
设置了格式: 英语(美国)
设置了格式: 英语(美国)
设置了格式: 英语(美国)
设置了格式: 字体: 加粗, 英语(美国)
设置了格式: 英语(美国)

设置了格式: 英语(美国)

设置了格式: 英语(美国)

设置了格式: 英语(美国)

带格式的: 缩进: 左侧: -0.5 厘米, 右侧: -0.24 厘米, 边框: 底端: (单实线, 自动设置, 0.5 磅 行宽)

Supplemental Data. Wu, Hou et al. (2023). TDF1 promotes tapetal cell differentiation. Plant Cell.

~~Supplemental Data. Wu et al. (2023). Plant Cell.~~

设置了格式: 英语(美国)

设置了格式: 英语(美国)

设置了格式: 英语(美国)

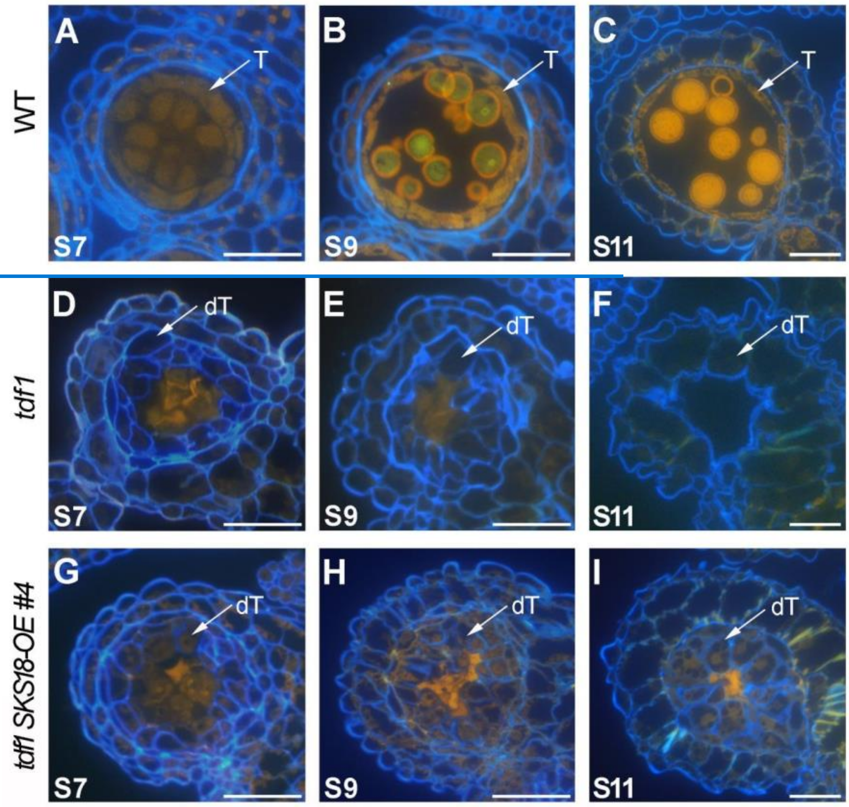

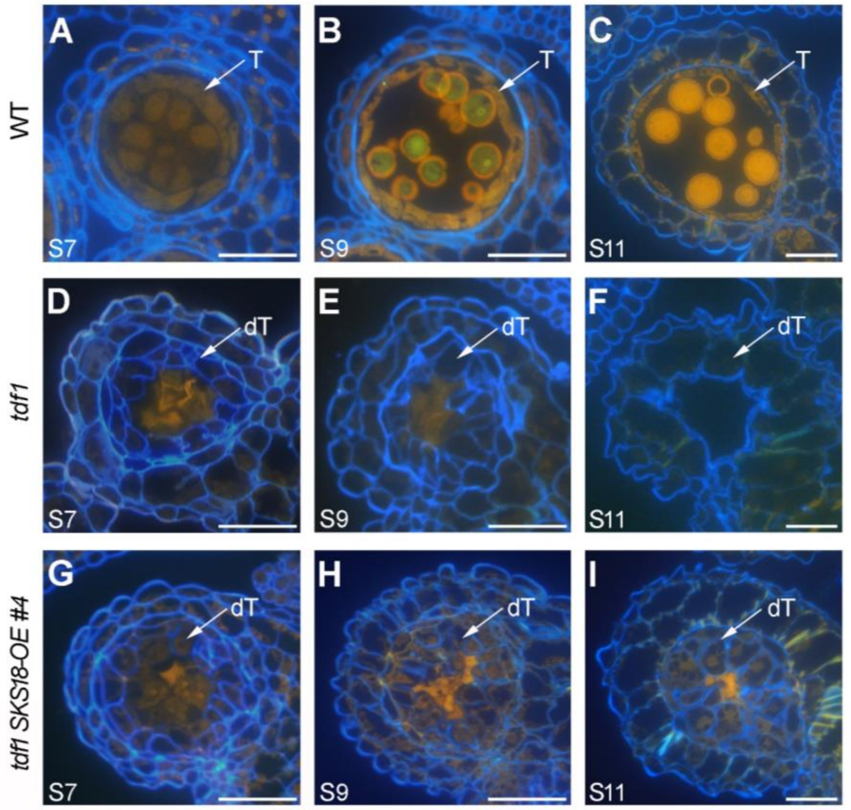

Supplemental Figure S8. Pollen wall materials biosynthesis in *tdf1 SKS18-OE* transgenic plants (Supports Figure 4).

Cytochemical staining of semithin sections of WT **(A-C)**, *tdf1* **(D-F)** and *tdf1 SKS18-OE #4* plants **(G-I)**. DIOC$_2$ stains the fatty acid contents with a fluorescent signal in orange and t~~T~~inopal stains the cellulose materials with a fluorescent signal in blue. Scale bars, 20 μm. T, tapetum; dT, defective tapetum.

设置了格式: 英语(美国)

设置了格式: 英语(美国)

设置了格式: 英语(美国)

带格式的: 缩进: 左侧: -0.5 厘米, 右侧: -0.24 厘米, 边框: 底端: (单实线, 自动设置, 0.5 磅 行宽)

设置了格式: 英语(美国)

设置了格式: 英语(美国)

带格式的: 缩进: 左侧: -0.52 厘米, 右侧: -0.33 厘米

设置了格式: 英语(美国)
设置了格式: 英语(美国)
设置了格式: 英语(美国)

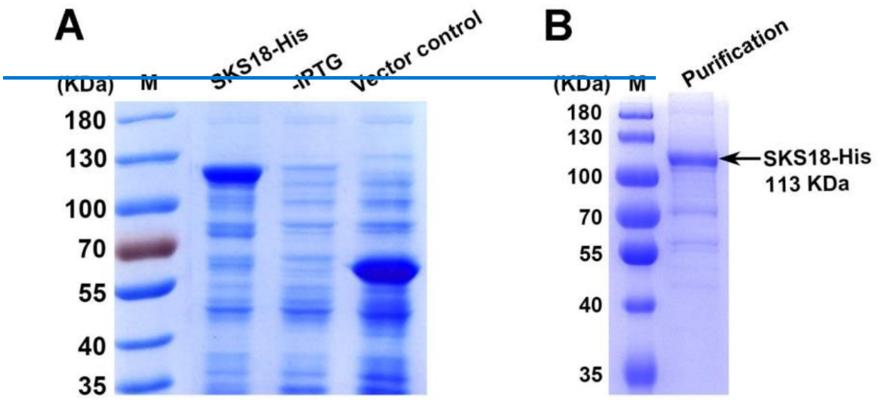

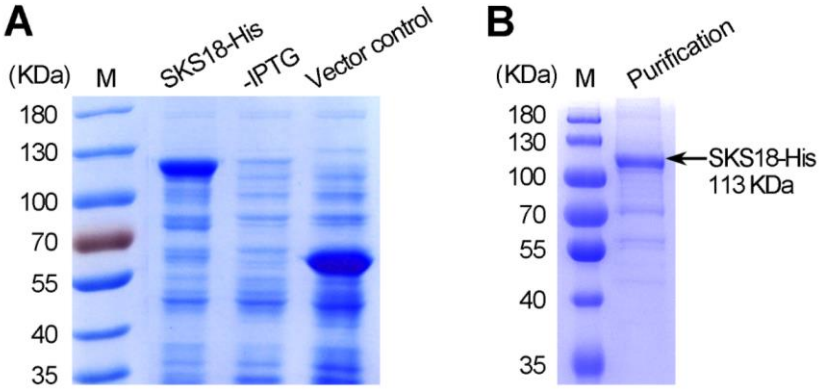

Supplemental Figure S9. SDS-PAGE analysis of recombinant SKS18 protein (Supports Figure 5).

(A) SDS-PAGE analysis of SKS18-His proteins used for *in vitro* enzymatic activity. **(B)** Purified SKS18 proteins were run in on an 8% gradient gel and stained with Coomassie Brilliant Bblue. M, protein markers.

设置了格式: 英语(美国)
带格式的: 缩进: 左侧: -0.52 厘米, 右侧: -0.33 厘米

Supplemental Data. Wu et al. (202

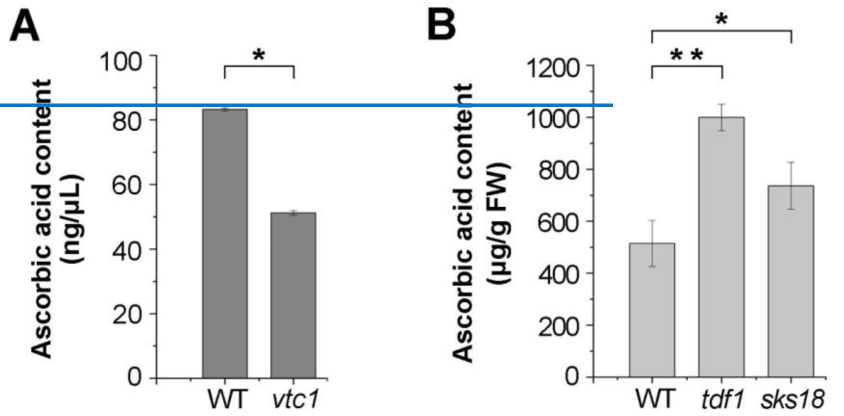

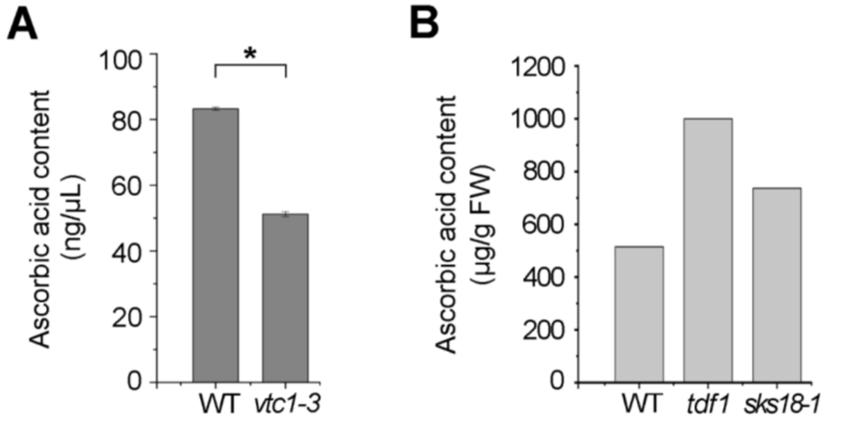

Supplemental Figure S10. AsA content measurements (Supports Figure 5). **(A)** Measurement of AsA content in *vtc1*-3 inflorescences under short-day light conditions. Data are means ± SD from three biological replicates. **P* < 0.05 (t-test). **(B)** The AsA content in inflorescences from WT, *tdf1* and *sks18*-1 plants was as determined by LC-MS/MS. Data are means ± SD from two biological replicates. Two biological replicates were performed**P* < 0.05; ***P* < 0.01 (t-test).

Supplemental Data. Wu et al. (202

设置了格式: 英语(美国)

设置了格式: 英语(美国)

设置了格式: 英语(美国)

带格式的: 缩进: 左侧: -0.5 厘米, 右侧: -0.33 厘米

设置了格式: 英语(美国)

批注 [PSC5]: Correct?

批注 [楼6R5]: Yes, you are right.

设置了格式: 英语(美国)

设置了格式: 英语(美国)

带格式的: 缩进: 左侧: -0.52 厘米, 右侧: -0.33 厘米

设置了格式: 英语(美国)

设置了格式: 英语(美国)

设置了格式: 英语(美国)

批注 [PSC7]: Do you mean that each bar is the mean of two values? This is not appropriate, as a mean and SD assume a normal distribution for the data, which cannot be assumed with n=2.
Perhaps you calculated the mean from all technical replicates of both biological replicates?

批注 [楼8R7]: This is my mistake! The each bar is the mean of two values from two biological replicates. I replaced the figure and revised the figure legends, thanks a lot!

3). Plant Cell.

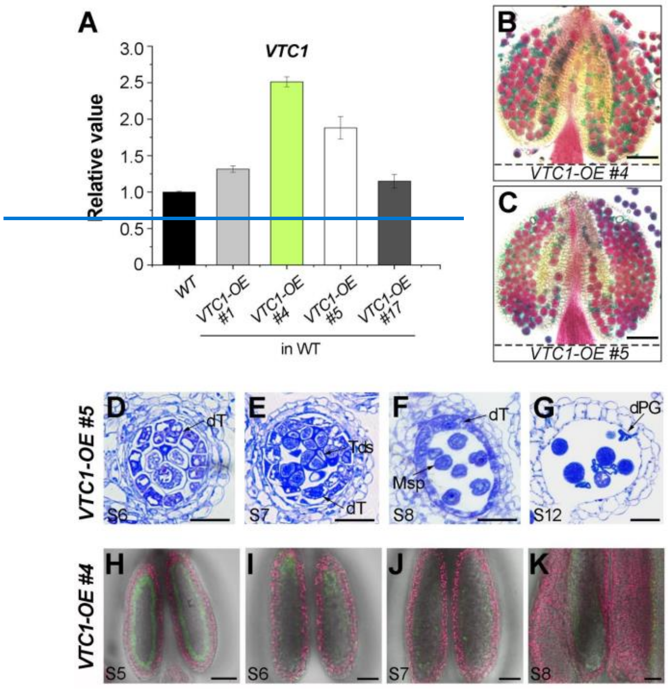

设置了格式: 英语(美国)

设置了格式: 英语(美国)

设置了格式: 英语(美国)

带格式的: 居中, 缩进: 左侧: -0.5 厘米, 右侧: -0.33 厘米

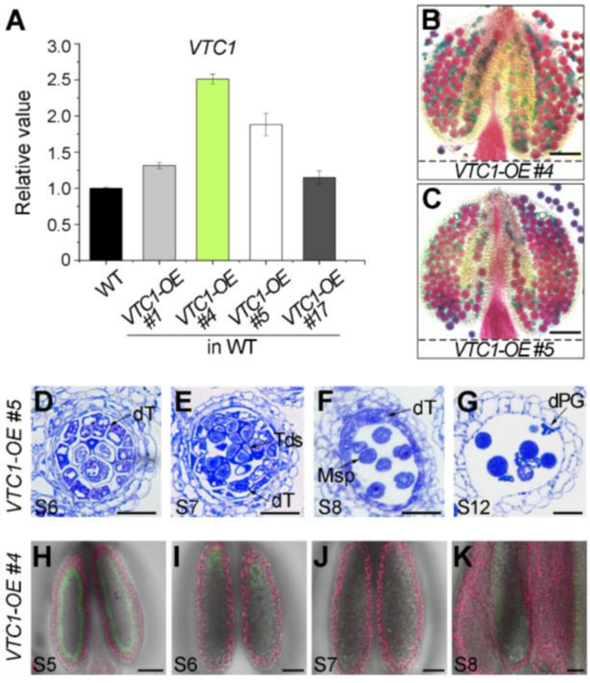

Supplemental Figure S11. ~~The~~ Eextra tapetal cells observed in the *VTC1-OE* transgenic plants (Supports Figure 5).

(A) *VTC1* expression ~~is detected~~ in inflorescences form WT and different *VTC1-OE* transgenic plants by ~~q~~RT-qPCR analysis. ~~Error bars represent the~~Data are means ± SD. Three biological repeats were performed, with similar results. Each biological replicate was performed with three technical replicates ~~for qRT-PCR~~. **(B and C)** Alexander's staining of anthers from *VTC1-OE #4* **(B)** and *VCT1-OE #5* plants **(C)**. Scale bars, 20 μm. **(D-G)** Semithin sections of anthers from *VTC1-OE #5* at the indicated stages. Scale bars, 20 μm. **(H-K)** VTC1-GFP protein ~~is located~~localizes in the tapetum from ~~the~~ *VTC1-OE #4* transgenic plants at stages 5-8. Chloroplast autofluorescence is shown in magenta. Scale bars, 20 μm. Msp, microspore; Tds, tetrads; dT, defective tapetum; dPG, degenerated pollen grain.

~~Supplemental Data. Wu et al. (202~~

设置了格式: 英语(美国)
设置了格式: 英语(美国)
设置了格式: 英语(美国)
设置了格式: 英语(美国)
带格式的: 缩进: 左侧: -0.52 厘米, 右侧: -0.33 厘米
设置了格式: 英语(美国)
设置了格式: 英语(美国)
设置了格式: 字体: 加粗
设置了格式: 英语(美国)
设置了格式: 英语(美国)
设置了格式: 英语(美国)
设置了格式: 英语(美国)
设置了格式: 英语(美国)
设置了格式: 英语(美国)

Supplemental Data. Wu, Hou et al. (2023). TDF1 promotes tapetal cell differentiation. Plant Cell.

3). Plant Cell.

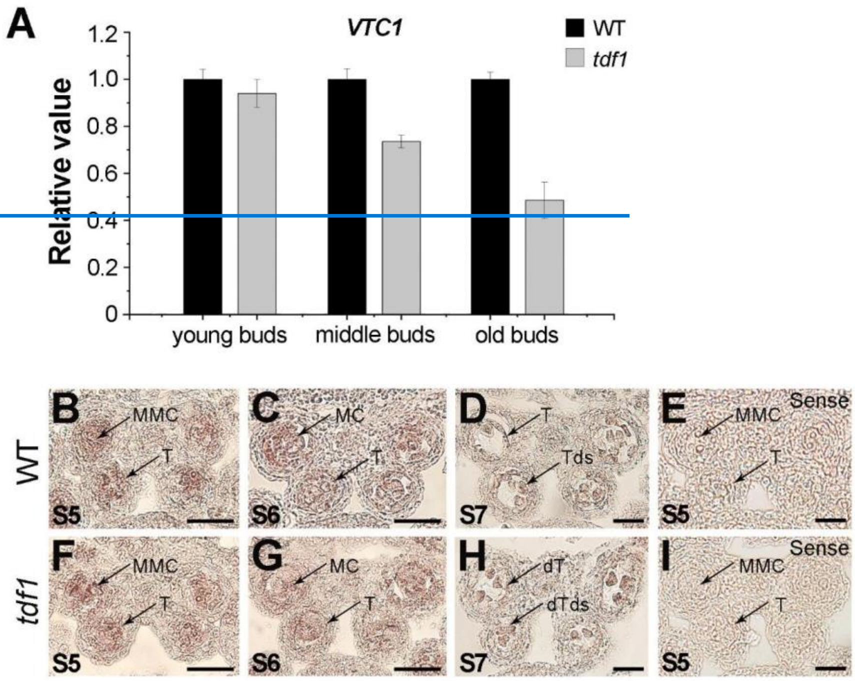

设置了格式: 英语(美国)

设置了格式: 英语(美国)

设置了格式: 英语(美国)

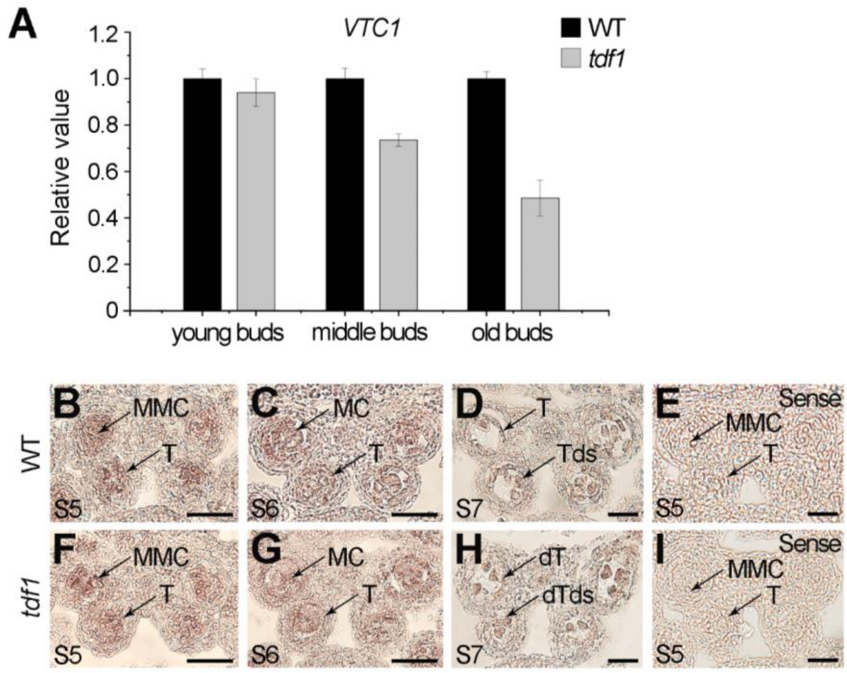

Supplemental Figure S12. Expression analysis of *VTC1* in the *tdf1* mutant (Supports Figure 6).
(A) Based on the developmental stages of anther, the buds were divided into three groups: young buds at stages 1-8; middle buds at stages 9-12; old buds beyond stage 12. *VTC1* expression ~~is~~ was ~~detected~~ measured in th~~e~~ose three groups from WT and *tdf1* plants by ~~q~~RT-qPCR analysis. ~~Error bars represent the~~ Data are means ± SD. Three biological repeats were performed with similar results. Each biological replicate was performed with three technical replicates ~~for qRT-PCR~~. **(B-H)** RNA *in situ* hybridization of *VTC1* transcript in anthers of WT **(B-D)** and *tdf1* **(F–H)** using an antisense probe. *VTC1* transcript in anthers of WT **(E)** and *tdf1* **(I)** using a sense probe. Scale bars, 20 μm. MMC, mother microspore cell; MC, meiocytes; T, tapetum; Tds, tetrads; dT, defective tapetum; dTds, defective tetrads.

~~Supplemental Data. Wu et al. (202~~

设置了格式: 英语(美国)
设置了格式: 英语(美国)
设置了格式: 英语(美国)
设置了格式: 英语(美国)
设置了格式: 英语(美国)
带格式的: 缩进: 左侧: -0.52 厘米, 右侧: -0.33 厘米
设置了格式: 英语(美国)
设置了格式: 英语(美国)
设置了格式: 英语(美国)
设置了格式: 英语(美国)
设置了格式: 字体: 加粗
设置了格式: 英语(美国)
设置了格式: 英语(美国)

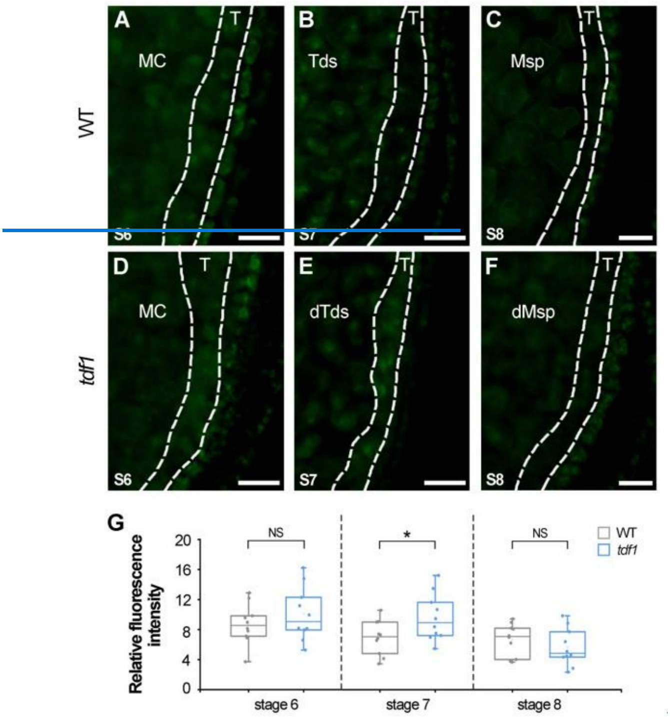

设置了格式: 英语(美国)

设置了格式: 英语(美国)

设置了格式: 英语(美国)

带格式的: 居中, 缩进: 左侧: -0.5 厘米, 右侧: -0.33 厘米

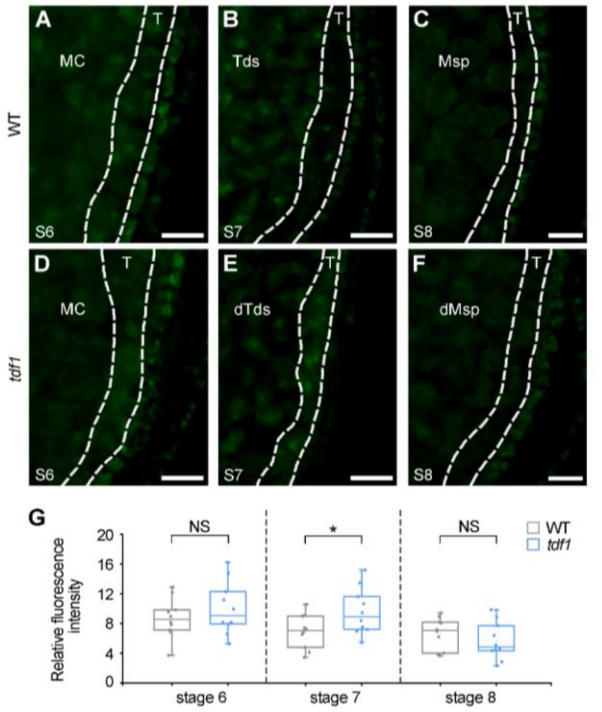

Supplemental Figure S13. Analyses of O_2^{-} in tapetum from ~~WT~~ wild-type and *tdf1* anthers (Supports Figure 7).

DHE staining analysis of O_2^{-} in tapetum (white dotted lines) from WT **(A-C)** and *tdf1* **(D-F)** anthers at stages 6-8. Scale bars, 20 μm. **(G)** Fluorescence quantification of O_2^{-} levels in tapetum from WT and *tdf1* at stages 6-8 based on DHE staining, shown as boxplots. The box ~~bounds~~ represents the interquartile range, the central line indicates ~~divided by~~ the median ~~(central lines)~~, and the Tukey-style whiskers extend to a maximum of 1.5× interquartile range from 25th and 75th percentiles. n = 10 (anthers at stages 6-8). *$P < 0.05$; NS, not significant (t-test). MC, meiocytes; Msp, microspore; T, tapetum; Tds, tetrads; dT, defective tapetum; dTds, defective tetrads; dMsp, defective microspore.

~~Supplemental Data. Wu et al. (202~~

设置了格式: 英语(美国)
设置了格式: 英语(美国)
设置了格式: 英语(美国)
设置了格式: 英语(美国)
设置了格式: 英语(美国)
设置了格式: 英语(美国)
设置了格式: 英语(美国)
设置了格式: 英语(美国)
带格式的: 缩进: 左侧: -0.52 厘米, 右侧: -0.33 厘米
设置了格式: 英语(美国)
带格式的: 缩进: 左侧: -0.52 厘米, 右侧: -0.33 厘米, 段落间距段后: 2.2 磅
设置了格式: 英语(美国)
设置了格式: 英语(美国)
设置了格式: 英语(美国)
设置了格式: 英语(美国)
带格式的: 缩进: 左侧: -0.5 厘米, 右侧: -0.33 厘米

Supplemental Data. Wu, Hou et al. (2023). TDF1 promotes tapetal cell differentiation. Plant Cell.

~~3). Plant Cell.~~

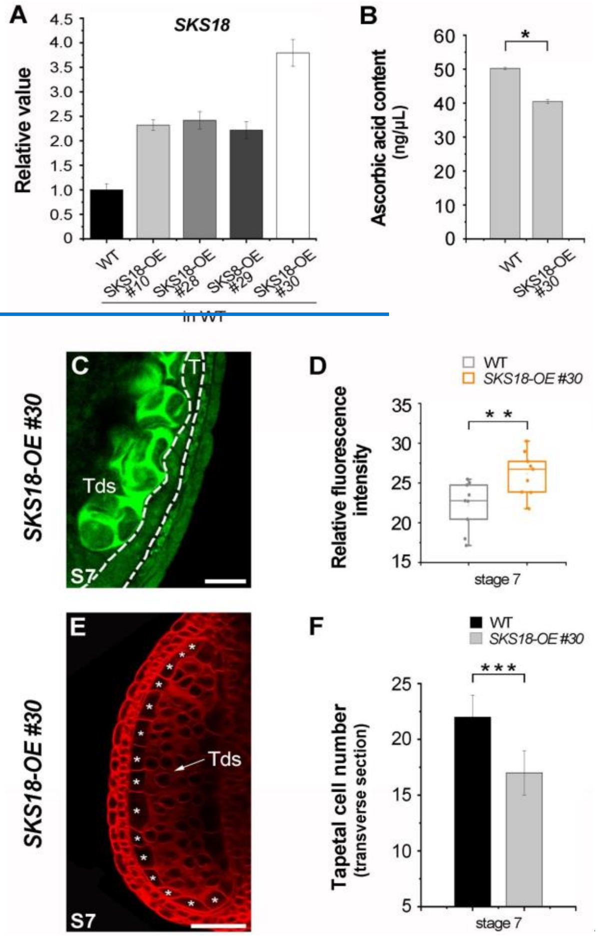

设置了格式: 英语(美国)

设置了格式: 英语(美国)

设置了格式: 英语(美国)

带格式的: 居中, 缩进: 左侧: -0.5 厘米, 右侧: -0.33 厘米

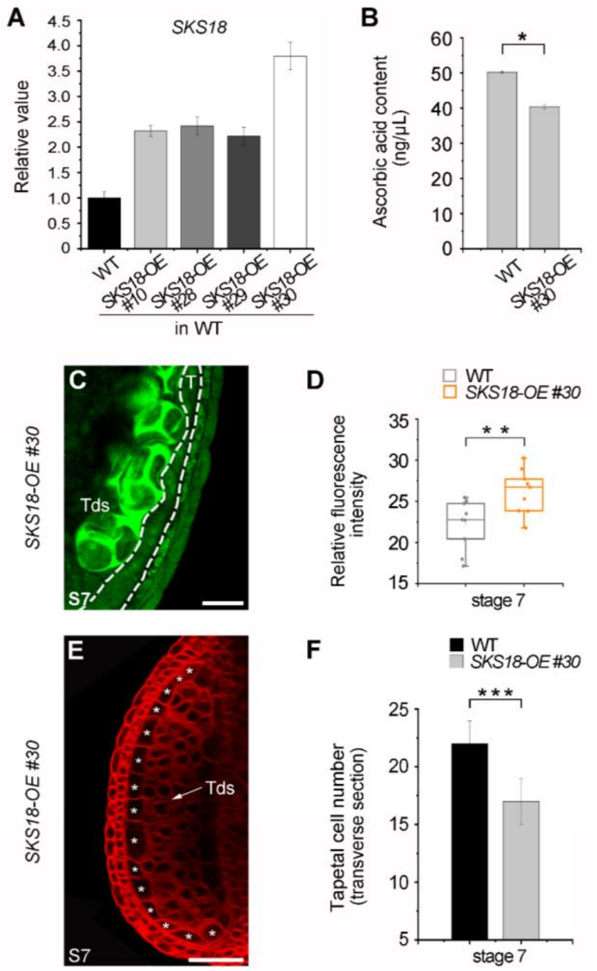

Supplemental Figure S14. The tapetal cells were decreased in the *SKS18-OE*

设置了格式: 英语(美国)
设置了格式: 英语(美国)
设置了格式: 英语(美国)
设置了格式: 英语(美国)
带格式的: 缩进: 左侧: -0.52 厘米, 右侧: -0.33 厘米

transgenic plants have fewer tapetal cells (Supports Figure 8).

(A) *SKS18* expression is detected in inflorescences form WT and different *SKS18-OE* transgenic plants by qRT-qPCR analysis. Error bars represent theData are means ± SD. Three biological repeats were performed, with similar results. Each biological replicate was performed with three technical replicates for qRT-PCR. **(B)** Measurement of AsA contents in inflorescences from WT and *SKS18-OE #30* plants. Data are means ± SD from three biological replicates. **P* < 0.05 (t-test). **(C)** H_2DCF-DA staining analysis of H_2O_2 in tapetum (white dot lines) from *SKS18-OE #30* anthers at stage 7. Scale bars, 20 μm. **(D)** Fluorescence quantification of H_2O_2 levels in tapetum from *SKS18-OE #30* based on H_2DCF-DA staining, shown as boxplots. The box bounds the interquartile range divided by the median (central lines), and the Tukey-style whiskers extend to a maximum of 1.5× interquartile range from 25th and 75th percentiles. n = 9 (anthers at stage 7). ***P* < 0.01 (t-test). **(E)** One abaxial locule in anther is was stained with FM4-64 and the tapetal cells (asterisks) are present in *SKS18-OE #30* plants at stage 7. Scale bars, 50 μm. **(F)** Statistical analysis showing tTapetal cell number based on the FM4-64 staining in WT and *SKS18-OE #30* plants from stage 7. Data are means ± SD. n=30 anthers. ****P* < 0.001 (t-test). T, tapetum; Tds, tetrads.

设置了格式: 英语(美国)
设置了格式: 英语(美国)
设置了格式: 英语(美国)
设置了格式: 英语(美国)
设置了格式: 英语(美国)
带格式的: 缩进: 左侧: -0.52 厘米, 右侧: -0.33 厘米
设置了格式: 英语(美国)
设置了格式: 英语(美国)
设置了格式: 英语(美国)
设置了格式: 英语(美国)
设置了格式: 英语(美国)
设置了格式: 英语(美国)
设置了格式: 英语(美国)
设置了格式: 英语(美国)
设置了格式: 英语(美国)
设置了格式: 英语(美国)

Supplemental Data. Wu et al. (2023). Plant Cell.

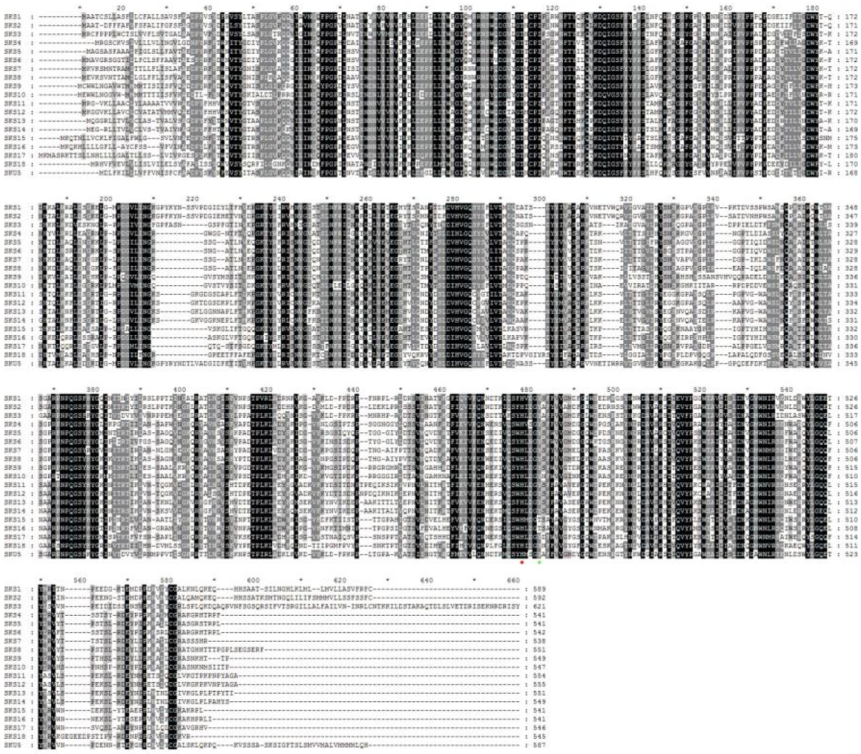

Supplemental Figure S15. Multiple protein sequence alignments of SKS family proteins members in Arabidopsis (Supports Figure 5). The sequences were aligned using ClustalW and displayed using BOXSHADE (http://www.ch.embnet.org/software/BOX_form.html). Red circle, the histidine residue for copper binding site; Green circle, the tyrosine residue.

设置了格式: 英语(美国)
设置了格式: 英语(美国)
设置了格式: 英语(美国)

批注 [PSC9]: You show only the alignment, but not the tree. Still, I would suggest providing a supplemental file with the alignment, in FASTA format.

批注 [楼10R9]: I provided the FASTA format in a PDF as the Supplemental Data Set 4, is it OK?

设置了格式: 英语(美国)
设置了格式: 英语(美国)
设置了格式: 英语(美国)
设置了格式: 英语(美国)
带格式的: 缩进: 左侧: -0.52 厘米, 右侧: -0.33 厘米
设置了格式: 英语(美国)
设置了格式: 英语(美国)
设置了格式: 英语(美国)
域代码已更改
域代码已更改

设置了格式: 英语(美国)

设置了格式: 英语(美国)

设置了格式: 英语(美国)
