# Supplementary material for: Ascorbic acid-mediated reactive oxygen species homeostasis modulates the switch from tapetal cell division to cell differentiation in Arabidopsis
Source: Plant Cell. 2023 Feb 14;35(5):1474–95. doi: 10.1093/plcell/koad037 (PMC10118275; doi:10.1093/plcell/koad037)
Supplement: koad037_Supplementary_Data [file tpc22.00796.PeerReviewReport.pdf › tpc22.00796.PeerReviewReport.pdf]

Ascorbic acid-mediated reactive oxygen species homeostasis modulates the switch from tapetal cell division to cell differentiation in Arabidopsis

Si-Yuan Wu, Ling-Li Hou, Jun Zhu, Yi-Chen Wang, Yu-Ling Zheng, Jian-Qiao Hou, Zhong-Nan Yang, Yue Lou

Corresponding author: Yue Lou (louyue5@shnu.edu.cn)

Review timeline:

**TPC2022-RA-00796**	Submission received:	August 18, 2022
	1st Decision:	September 17, 2022 *revision requested*
**TPC2022-RA-00796R1**	1st Revision received:	September 19, 2022
	2nd Decision:	November 24, 2022 *revision requested*
**TPC2022-RA-00796R2**	2nd Revision received:	January 7, 2023
	3rd Decision:	January 11, 2023 *accept with minor revision*
**TPC2022-RA-00796R3**	3rd Revision received:	January 14, 2023
	4th Decision:	January 18, 2023 *acceptance pending, sent to sci editor*
	Final acceptance:	Feb. 3, 2023

REPORT: (The report shows the major requests for revision and author responses. Minor comments for revision and miscellaneous correspondence are not included. The original format may not be reflected in this compilation, but the reviewer comments and author responses are not edited, except to correct minor typographical or spelling errors that could be a source of ambiguity.)

TPC2022-RA-00796 1st Editorial decision – *revision requested* **September 17, 2022**

We have received reviews of your manuscript entitled "Ascorbic acid-mediated ROS homeostasis modulates the switch from tapetal cell division to cell differentiation in Arabidopsis." Thank you for submitting your best work to The Plant Cell. The editorial board agrees that the work you describe is substantive, falls within the scope of the journal, and may become acceptable for publication, pending revision and potential re-review.

We ask you to pay attention to the points raised by the three reviewers in preparing your revision. They make a range of different points from somewhat major to quite minor, but these points all seem addressable and would improve your work.

--- Reviewer comments:

TPC2022-RA-00796R1 1st Revision received **September 19, 2022**

Reviewer comments and **author responses**:

Reviewer #1:

In this manuscript, the authors found that tdf1 mutant produces multilayered tapetum, resulting in male sterility. From past transcriptomic analyses of tdf1 mutant, they focused on SKS18 which encodes a multicopper oxidase-like protein to catalyse AsA, known as an antioxidant to ROS. TDF1 was evidenced to directly bind to the upstream MYB-binding site of SKS18 gene by ChIP-qPCR and subsequent EMSA. The authors showed that SKS18 is specifically expressed in tapetum and partly contributes to catalysing AsA and modulating ROS level by in situ hybridization, SKS18-overexpression in tdf1 background, enzymatic activity measurements, H2DCF-DA staining and so on. It was also proven that VTC1 contributes to synthesize AsA in tapetum, and TDF1 negatively regulates VTC1 expression and modulates AsA accumulation. Eventually the authors concluded that the balance of AsA contents in tapetum modulated by TDF1/SKS18 and VTC1 is important to switch from cell division to differentiation.
The story is well documented and supported by a lot of experiments conducted appropriately. Most findings are novel

and the text was well written. Thus I think this paper worthy to be published. However, I have several major and minor questions as follows.

Thanks very much for taking your time to review this manuscript. We really appreciate all your comments and suggestions!

Major points

(1) In Fig. 6, the authors quantified the intensity of H2DCF-DA signal, and claimed that ROS level is reduced in sks18 compared to WT (Fig. 6M). However, there is no description how the intensity is normalized in Method section (L574-581). For example, in Fig. 6H, the signal intensity seems weaker entirely in sks18, compared to that of WT, not only in tapetum, but also tetrads and other wall layers. A difference is subtle between the signal intensities of WT and sks18 tapetum. Thus I think it necessary to normalize the value by the value of internal control for example, otherwise we cannot compare the intensity as shown in Fig. 6M appropriately and precisely.

Answer: For the ROS intensity quantification, we consulted several references about ROS staining in anthers from both Arabidopsis and rice (Zheng et al., *PNAS*, 2019; Xie et al., *Plant Cell*, 2014; Hu et al., *Plant Cell*, 2011). In their articles, they also stained the anthers from WT and mutant by ROS dye, and compared the fluorescence signal of mutant with the one of WT. Therefore, it is a common method to regard the WT ROS intensity as a control and compare the sample intensity with the WT one. Since we had the similar experimental purpose, we followed their ROS staining and quantification method.

In our ROS staining experiment, we took the images with a relative consistent staining degree to exclude the staining interference. Even so, sometimes the tapetal cells in one image have different fluorescence intensities. Then, four different areas with different ROS intensities in a single tapetum were measured, and finally the means were calculated from about 10 independent pictures. All of these could reduce the error and improve our results quality.

It's true that the signals between WT and *sks18* are not easily distinguished on a visual level. As to Fig. 6H, the signals in other anther layers seem reduced due to our focusing the tapetum during photographing. But, the signals in tetrads did reduce in the most of *sks18* images. Given the SKS18 surrounds the tetrads at stage 7 (Fig. 2U), we supposed that the mutation of SKS18 may also affect the ROS accumulation in tetrads. In this revision, we replaced another *sks18* image at stage 7. In Fig. 7H, the ROS intensities of epidermis and endothecium were similar with those in WT, while signals in the tapetum and tetrads were relatively weaker. Additionally, we re-name the Y-axis in Fig. 7M; added the contents about how to quantify the tapetal ROS intensity in method section. We also provided the statistical data in the supplemental dataset table. Hoping all these data could better to show how we get this result and support our conclusions. Thanks for your precious suggestions!

(2) According to Tsukagoshi et al. (2010), increased level of hydrogen peroxide tends to repress cell division in root meristem area, and superoxide, another ROS, maintains cell division. So I think specifying what kinds of ROS repress the division and direct cells to differentiation in tapetum is important in this manuscript. What kinds of ROS does H2DCF-DA stain? In this sense, I recommend to use more ROS-staining chemicals to specify the species of ROS decreased in tdf1 tapetum.

Answer: Currently, the 2',7'-dichlorofluorescein diacetate (H_2DCFDA) is the most-used fluorescent dye to study hydrogen peroxide (H_2O_2) signaling dynamics (Kim et al., *Plant J*, 2021; Fichman et al., *Mol. Plant*, 2019;). Our results also support the finding that the elevated H_2O_2 repress cell division to promote cellular differentiation (Tsukagoshi et al., *Cell*, 2010). Therefore, it is worth to find whether superoxide ($O_2^{\cdot-}$) levels will be affected or not. We used dihydroethidium (DHE), a fluorescent dye, to detect the $O_2^{\cdot-}$ levels in anthers from WT and *tdf1* at stages 6-8 (Fig. S13). In WT, compared with the H_2O_2 levels, the whole $O_2^{\cdot-}$ levels showed an opposing accumulation tendency (Fig. S13, A-C and G). This finding supports that the accumulation of $O_2^{\cdot-}$ is not primarily required when the tapetum goes through the cell differentiation. However, at stage 7, the $O_2^{\cdot-}$ levels in *tdf1* were higher than that in WT (Fig. S13, B and E), suggesting that the increased $O_2^{\cdot-}$ levels may also induce the tapetal cell division. Moreover, in *tdf1* tapetum, the change of $O_2^{\cdot-}$ levels varied much less than that of H_2O_2 (Fig. 7M and S13G). Based on these results, we supposed that H_2O_2 may play a dominant role in controlling the transition from cell division to differentiation in the tapetum.

It's interesting why $O_2^{\cdot-}$ levels were not reduced when AsA is over-accumulated in *tdf1* tapetum. H_2O_2 is more stable

than $O_2^{\cdot -}$ (Pitzschke et al., *Antioxid. Redox Signal*, 2006) and $O_2^{\cdot -}$ can be transformed into H_2O_2 through enzymatic activity such as peroxidase (Elstner and Heupel, *Anal. Biochem*, 1976). AsA is an inhibitor of peroxidase (Fry, *Annu. Rev. Plant Physiol*, 1986). We therefore infer that high AsA contents might inhibit some peroxidases and make $O_2^{\cdot -}$ unable to transform into H_2O_2, which let $O_2^{\cdot -}$ accumulate in *tdf1*. Anyway, this is a very nice suggestion which makes our work more accurate and we added the relevant results in the text, thanks!

(3) SKS18-dependent AsA catalysis and its impact on scavenging ROS seems very limited in tapetum. The reasons are as follows; first, the tapetal cell number is slightly increased in sks18, but the increase level is very low, compared to tdf1. Second, the authors claim that SKS18-OE in tdf1 background partially recovers defects in tapetum, based on partial recovery of tapetal cell numbers (Fig. 3) and fatty acid expression (Fig. S6). Especially, DIOC2 signals suggesting the recovery are very faint in defective tapetum in Fig. S6. Third, sks18 anthers produce seemingly normal microspores (Fig. 3J). Finally, the authors claim that ROS level is reduced in sks18 compared to WT (Fig. 6M). However, as far as judged from H2DCF-DA staining images, the staining level in tapetum is not so different between WT and sks18 (Fig. 6B, H) and the quantification results (Fi. 6M) didn't convince me due to the reason as described in question (1).

Rather, negative regulation of VTC1 by TDF1 seems a major pathway to modulate AsA and ROS levels. Despite of such important roles, there are few descriptions about TDF1-dependent regulatory mechanisms of VTC1 expression. So, the authors should show the expression pattern of VTC1 in tdf1 mutant by their past transcriptomic data or in situ hybridization and discuss it more in the text. I think it beneficial for better understanding of readers.

Answer: We had performed the expression of *VTC1* in *tdf1* inflorescences (discarding buds beyond stage 12) by qRT-PCR, and the results showed no obvious difference in change of the *VTC1* transcripts between WT and *tdf1*. Then, we wondered whether VTC1 proteins in *tdf1* anthers were affected or not. As you say, it's still necessary to mention this information in the manuscript! Therefore, this time we divided the buds into three groups based on the anther stages. The result showed that *VTC1* transcripts in *tdf1* were similar with those in WT before stage 9 (Fig. S12A), meanwhile the VTC1 proteins were still highly enriched in *tdf1* anthers (Fig. 6F-I). Moreover, RNA *in situ* hybridization confirmed that the expression pattern of *VTC1* in WT and *tdf1* showed no obviously difference at stages 5-7 (Fig. S12, B-I). So, all these results show that TDF1 mainly regulates the accumulation of VTC1 protein instead of the *VTC1* transcripts.

Minor points

(4) Regarding to the question (3), contribution of VTC1 in modulation of AsA and ROS levels seems larger than that of SKS18. However, in Fig. 8, VTC and SKS18 are equivalently placed in TDF1 downstream. I am afraid of it misleading many readers.

Answer: In this manuscript, we found that TDF1 acts as a negative regulator of AsA accumulation via controlling AsA synthesis and AsA oxidation pathways. This is one of the novelties in our findings. So, in this model, TDF1-VTC1 module and TDF1-SKS18 module primarily represent two independent AsA metabolism pathways. Although the current results show that the contribution of VTC1 seems larger than that of SKS18, but we cannot exclude the possibility that other SKS members may also involve in this modulation. In fact, we have found that other SKS proteins locate in the tapetum, too. It's possible that *sks* double and/or multi-gene mutants will have more severe phenotype in tapetal cells. Based on these considerations, we mainly want to elucidate how TDF1 regulates the AsA homeostasis instead of emphasizing which pathway contributes more to affect the AsA accumulation. Hoping all of these could provide you a reasonable explanation.

(5) It was difficult for me to understand a logic described in L154-160 describing KEULE-GFP analysis. Does "a high probability to observe GFP signals related to a misoriented cell plat pattern" mean that cells increased in tdf1 mutation are silent in division activity? I recommend to revise these sentences to explain the results more correctly.

Answer: We have revised these sentences and added a column diagram to show our statistical data (Fig. 1P), thanks!

Reviewer #2:

Comments to Authors: The work presented in this paper is a great addition to the tapetal development story. The authors have carefully leveraged the available mutants in Arabidopsis and successfully used morphological markers

of tapetal differentiation to show that TDF1 inhibits extra cell divisions in the tapetum, a key marker of the transition to a differentiated cell type (and a common phenotype in early male-sterile mutants where tapetal divisions persist!). The connection with the AsA antioxidant and ROS homeostasis via SKS18 is well-established in this study and fits with a lot of the established knowledge around the important of ROS in plant development. Overall, the study is well-executed, and clearly a large amount of work went into the experiments. There are a few places in the text where imprecise language or style make conclusions confusing or ambiguous; I've highlighted these and a range of English-language style corrections in my minor revisions list below. The language revision that I take most issue with is in the abstract where TDF1 is claimed to inhibit "tapetal cell division and initiates cell differentiation". As the authors point out in many other places in the text, the ceasing of cell division is part of the switch from specification to differentiation, but the data presented here does not prove that TDF1 is the single factor to directly initiate differentiation; I've suggested more moderate language below. Differentiation is a process defined (historically) by morphological markers and claiming that you've found "the switch" is a high bar to clear.
In addition to these minor revisions, and some figure organization changes (change those magenta arrowheads to a different color!), there is one key piece of data that I'd like to see re-done or differently interpreted: the quality of the GFP images in Figure 5 (see my comments in major revisions). In addition to improving those Figure 5 images, I also want some clarification added to the in situ hybridization images, and quantification of the KEULE-GFP in Figure 2 added to improve the ability of the reader to interpret the data themselves - not just take the authors words on trends in images.

Thanks very much for taking your time to review this manuscript. We really appreciate all your comments and suggestions! It's our honor that you so patiently modify our language, which indeed improves our manuscript a lot!

Major Revisions:

In situ hybridizations (Line 205, Figure 2B and 2L). The text describes that the expression pattern is not affected between WT and ams, but to my eye 2L is much lighter than 2B and forms a complete ring around the locule compared to the WT that is broken. Were the WT sections poor, leading to broken locules? Were these in situs run in separate batches? When directly comparing in situ hybridization pattern and intensity it's important that the slides be dehydrated and taken through the hybridization and washing steps in the same batch. If these were not, or if the sections were damaged, they should be repeated. I would also include the sense control for the tdf1 samples. It's standard procedure to include the sense for all probes, but here only the WT and ams samples are shown hybridized to the sense probe; including all sense probes as a supplement if they don't fit in the main text is acceptable. If everything was in a single batch, please include that comment in the methods section.

Answer: Thanks for your suggestion to improve the quality of images. In this revision, we replaced Fig. 2B where the ring is a complete one. Actually, RNA *in situ* hybridizations between WT and *tdf1* were performed in a single batch, while the one for *ams* was performed alone. That's exactly right that two groups run in separate batches should not be compared with their signal intensities! That's our mistake! Therefore, in the text, we modified the description that the signal in *ams* could still be observed at stage 6. We also added the comment in the methods section as you mentioned. Moreover, combining the other reviewer's suggestions, we put the data about *ams* into the supplementary information (Fig. S4), which also included the images with all sense probes. Hoping these adjustments could meet the requirements, thanks again!

Figure 1N-1S: These images should have been capture at higher magnification and/or with more tapetal cells clearly in focus. Looking at the merges (1P and 1S) each basically has only 1-3 cells in focus to look at the KEULE-GFP expression pattern, and this does not give the impression claimed in the text of "high and low probability" of observing the cell plates in these cells. Images either need to be replaced with images where more cells are visible and in-focus, or the number of cells showing cytoplasmic versus cell-plate localization should be quantified similar to what is shown for cell number in panel 1M. Presumably the authors already have images they could do this quantification from, so it should be straightforward to add if higher quality representative images are too onerous to procure.

Answer: I totally agree with your comments! The part of KEULE-GFP including the data presentation and language description were not shown well that confused all the reviewers. We re-wrote this part in the text. As you say, it's too onerous to procure the perfect representative images. Therefore, we used the original images to indicate the

localization of KEULE-GFP (Fig 1. N and O). Based on our images, we quantified the number of tapetal cells showing cell-plate localization versus the total tapetal cells and provided a column diagram to indicate that the frequency of cell-plate appearance in *tdf1* was higher than those of WT (Fig. 1P). The relevant statistical data was also provided into the supplemental dataset table. Thanks for your great advice!

Figure 5A-5J. Without the white dotted lines outlining the tapetum in these images, I would often be unable to identify its location (and I've looked at 1000s of anther images). These images are not publication-quality in my opinion. Can the DIC/brightfield be left out? (The images in Figure 7 have no DIC and are easier to interpret). Could they be re-taken at higher magnification or with a cell-wall counterstain to better separate the layers? A different fixation method might also improve the resolution and clarity of the tissue. While I can agree with the authors that there is a difference between A-E and F-J, I cannot confirm any description about the changes between stages across developmental time based on this image set.

Answer: Thanks for your suggestion to improve the quality of images! In this revision, we discarded the DIC/brightfield and made those images with high resolution (Fig. 6A-J). We also adjusted the light of chloroplast autofluorescence which was shown in red. Usually, those autofluorescence only appear in the layers of epidermis and endothecium which could serve as a marker to separate the outer anther layers with the tapetum. Hoping these images could provide you a better judgment and achieve the publication-quality.

Minor Revisions (Itemized by line number in the text):

Line 26. Moderate the language around TDF1 directly triggering differentiation, as that link is not concretely proven by the data in this study. I'm more comfortable with "...inhibits tapetal cell division leading to cell differentiation." Instead of "division and differentiation" as is currently written.

Answer: I've changed this sentence; this modification makes our content more precise, thanks!

Line 59. Where "the mutation" is reference, the authors need to be more specific. What kind of mutation is this? Is it a null mutant? I know it's likely specified in the reference, but please state it plainly in the text here.

Answer: *vtc1* mutant is a point mutation, which was mutagenized with ethyl methanesulfonate. The mutation changes a single cytosine to thymine at position +64 relative to the first base of initiator methionine. It is not a null mutant (Conklin et al., 1999). I added this information and quoted the reference in the text, thanks.

Line 67 - 69. The sentence "In parallel, AAO mRNA and activity increase in non-dividing cells whereas they are low during cell division (Kato and Esaka, 1999; Pignochi et al., 2003), supporting that AAO is involved in mitotic activity." Seems contradictory with the sentence just before it. It may be wording or order of phrases. Consider rewriting this bit about the AAO mitotic activity connection.

Answer: I rewrote this sentence, thanks.

Line 80. Replace "microspores/developing" with "microspores and/or developing..." and add a citation to the end of this sentence to support this. It's a common observation, but worth citing!

Answer: I revised it and quoted the reference (Pacini et al., *Plant Systematics and Evolution*, 1985), thanks.

Lines 85 - 91. Make this into 2 sentences about tapetal morphological observations. Overall lines 85 - 98 are a good description of how tapetal differentiation is classically defined. Including a sentence that points out that tapetal "differentiation" has typically been defined by morphological features, including those listed would help with understanding for readers less well-versed in the topic.

Answer: This is a nice suggestion! I separated this into two sentences. Thanks for improving our language description.

Line 157 - 159. What qualified as "high" and "low" probability in these images? These should be updated with numbers, see a comment about Figure 1 N-P in major revisions.

Answer: I revised these results and provided a more straightforward image as you suggested (Fig. 1P), thanks.

Line 207 - 208. States that SKS18-GFP fluorescence was not affected, but it looks to have become more microspore-localized than tapetal. Please comment on this change in the text.

Answer: That's my mistake that the previous description was not accurate! Based on the fluorescence observation, we tried to elucidate that the SKS18-GFP protein can still be expressed in the tapetum even lack of the expression of AMS. Therefore, AMS is not required to activate the expression of *SKS18*. Since the *ams* swollen tapetal cells will occupy the locular space and crush the tetrads, we supposed that the locule-localization of SKS18-GFP is more likely the leakage from the tapetum. In this revision, a complete image without DIC/brightfield was shown (Fig. S4H) and I revised the description, thanks.

Line 216. Define how this promoter length was determined or predicted.

Answer: According to the TAIR information, we used the 1391bp fragment as the *SKS18* promoter. The genetic complementation proved that this promoter length is suitable for activating the *SKS18* expression. I slightly revised the sentence in the text, thanks.

Line 228. Somewhere in this paragraph there should be mention whether co-expression of these proteins is expected in planta. (Hopefully this is obvious to readers, but it's good to state the validity of a leaf protoplast assay).

Answer: In this revision, the nuclear localization of VENUS-TDF1 in the tapetum at stages 5-7 was shown in Fig. S5. This data could prove that proteins of TDF1 and SKS18 are co-expression in anthers. I also mentioned this information in the text. This suggestion makes our content more precise, thanks!

Line 239. Instead of "has no effect" it would be more accurate to describe the effect as not significantly different. The trend looks like there might a bit of inhibition, but the statistics cannot confirm that.

Answer: I revised this sentence following your suggestion, thanks.

Line 251. Why only occasionally? Please comment on why you think this is less than 100% of tapetum that becomes multilayered.

Answer: The phenotype of *sks18* is weaker than that of *tdf1*. Therefore, when we observed *sks18* by semi-thin sections, sometimes we cannot capture its multilayered phenotype. We used this word is to express that the phenotype of *sks18* is not as obvious as *tdf1*. Evidently, this word creates the ambiguity. I deleted it in this revision, thanks.

Figure 1A-1H. The red dotted boundaries are hard to see, please make then bolder.

Answer: I revised them following your suggestion, it did emphasize the phenotype and give a better visual effect, thanks.

Figure 1I-1L. The magenta arrowheads are not high contrast with the red cell walls. Change them here (and in all images) to be a higher contrast color! White or yellow would work well. These pictures were a bit grainy, make sure that the high-resolution revisions are included in the final upload.

Answer: I changed the color of arrowheads including all images, also provided the high-resolution images in this revision, thanks.

Figure 2X-Y, Z-B1. Please add more white space or a thin vertical line between these panels and those to their left. The letter order changes in a confusing way in panels P- Y. I wonder if this figure couldn't be separated into 2 where panels Z-B1 become their own figure?

Answer: This is a good suggestion! I separated the Figure 2 and also adjusted the layout in Figure 2, thanks.

Figure 2W. Could an apoplastic marker have been used to confirm that the change in localization is really to the apoplast?

Answer: *SKU5* is the first gene to be studied in the SKS members (Sedbrook et al., *Plant Cell*, 2002). In their article, since SKU5 has a peripherally-localized fluorescence in the root, they treated roots with mannitol to plasmolyze the cells. After plasmolysis, some signals were associated with Hechtian stands. We therefore followed their experimental protocol, and did find that the SKS18-GFP fluorescence was left from the plasma membrane to the apoplast. In this revision, we discard the white dot line and labelled the sites of apoplost, plasma membrane and cell

wall (Fig. 2P). Hoping this adjustment could provide a clear SKS18-GFP localization in the apoplast.

Figure 3U-X. Why are stages S6-S7 for sks18 compared with stages S7-S8 for the overexpression experiment? These stages should either be equivalent, or the reason for the difference should be explained in the text.

Answer: You are right! We replaced S8 with S6 for the *SKS18* overexpression line (Fig. 4W) and put the S8 into the Fig. S1, thanks.

Figure 3Y. Put the legend in the same order as the bars appear.

Answer: I changed this, thanks.

Reviewer #3:

tdf1 is a male-sterile mutant which has various stamen defects, including extra tapetal cells, abnormal vacuolation in all types of anther wall cells, and collapsed microspores. In this research the authors focused on the extra tapetal cell phenotype of tdf1. They showed that tdf1 tapetal cells did not differentiate properly but had prolonged cell division activity during development, suggesting that TDF1 inhibits tapetal cell divisions and promotes tapeal cell differentiations.
The authors found that TDF1 directly promoted the expression of an ascorbate oxidase gene Skewed5-similar 18 (SKS18) in the tapetal cells. TDF1 also negative regulated an ascorbate acid (AsA) biosynthetic enzyme VITAMIN C DEFECTIVE1 (VTC1). Increasing AsA level in inflorescences by knocking out of SKS18 or overexpressing VTC1 lead to increased numbers of tapetal cells. Overexpressing SKS18 in tdf1 mutant decreased AsA level and alleviated the tapetum defects, including extra tapetal cells. A mutation of VTC1 gene in tdf1 could also mitigate the defective tapetum. It was showed that increased levels of AsA were associated with decreased levels of reactive oxygen species (ROS) in tapetal cells and lower ROS levels could result in extra tapetal cells. Therefore, the authors proposed that AsA balances cell division and cell differentiation in tapetum through mediating ROS homeostasis. This manuscript showed detailed observations and analysis of tapetal cell differentiation and the mechanism underlying this process. The manuscript is written in a clear and accessible manner. It will advance our understanding of TDF1's controlling of tapetal cell differentiation.

Thanks very much for taking your time to review this manuscript. We really appreciate all your comments and suggestions!

However, it seems a little overstated to draw the conclusion that AsA balances cell division and cell differentiation in tapetum. This research suggested that AsA levels and tapetal cell function are not always tightly correlated, since tdf1 SKS18-OE plants have lower AsA level compared with VTC1-OE lines, but the former have much severe tapetal cell developmental defects. Besides, a two-fold increase in AsA levels in sks18 or VTC1-OE lines only result in roughly ten percent increase in tapetal cell number compared with WT, indicating that the cell division activity in majority of the tapetal cells are not affected by AsA accumulation.

Answer: Thanks for you so carefully evaluating our results! In this manuscript, we linked the phenotype of over-proliferated tapetal cells with the regulation of AsA metabolism by TDF1. We first found that *SKS18* is one of the targets controlled by TDF1. Then, according to the biochemical function of SKS18, we detected the AsA accumulation in different genetic materials.

TDF1 is a TF expressed at early anther stage (Zhu et al., *Plant J*, 2008 and Fig. S5), the expression of many downstream genes is altered and pathways directly or indirectly affected by its mutation (Lou et al., *New phytol*, 2018). It can be imaged that other factors may also involve in the regulation of AsA accumulation. Recently, we find other SKS members are expressed in the tapetum, suggesting that a functional redundancy may exist. The double and/or triple mutants probably will show a stronger defect in tapetal cell number. Alternatively, it cannot be excluded that pathways such as auxin and cytokinin signaling also function in this process. Therefore, when comparing the number of tapetal cells and the change of AsA levels, the same genetic background is considered such as *tdf1* versus *tdf1 SKS18-OE*, which could better to analyze the linkage between the AsA content and cell number.

It's true that not many tapetal cell are induced to divide in *sks18* or *VTC1-OE*, although they had a two-fold increase in AsA levels. However, there is a tendency that the more AsA levels induce the more tapetal cells. *tdf1* had the

highest level of AsA and the largest number of tapetal cells (Fig. 5B, 1M). While *sks18* had the lowest AsA levels and produces a weak phenotype (Fig. 5B, 4Y).

Based on our analysis of staining and statistics, we showed that, in the tapetum, increasing AsA content is able to promote the cell division while decreasing AsA content to cease the cell division. Thus, it's sure that AsA accumulation is one of pathways affecting the process of cell division and/or differentiation in the tapetum. This finding provides a breakthrough to explain a molecular mechanism underlying the regulation of tapetal cell division and/or differentiation. Hoping all of these could provide you a reasonable explanation.

In the meanwhile, all the mutants or transgenic lines used in this research had increased levels of AsA and increased tapetal cell numbers compared with WT. If AsA balances division and differentiation in tapetal cells, we would expect to see fewer tapetal cells if AsA levels in WT anthers was further decreased. It would be interesting to see the AsA levels and tapetal phenotypes in the vtc1 mutant or SKS18 overexpression lines.

Answer: This is a nice suggestion! We screened the *ProDYT1550:SKS18-GFP* overexpression lines and confirmed that the *SKS18-OE #30* showing the highest *SKS18* expression in T1 generation. We then quantified the number of tapetal cells in its T2 generation. Statistic data showed that the number of tapetal cells in these transgenic lines decrease compared with those of WT, which did agree with your assumption (please see below). We also found that the size of some tapetal cells is enlarged (please see below). We infer that overexpressing SKS18 may further decrease the AsA levels and impact on ROS homeostasis, which not only affects the cell division but also the cell expansion in the tapetum. In plants, the step of cell differentiation is often marked by an increase in cell size (De Veylder et al., *Nat. Rev. Mol. Cell. Biol,* 2007), we therefore supposed that the tapetal differentiation instead of cell division may prevail in those *SKS18* overexpression lines. Since these results still need more experimental confirmation, we only show the relevant results in this response. We believe that addressing this question will gain further insights into mechanism underlying the specification and development of tapetum. Thanks a lot!

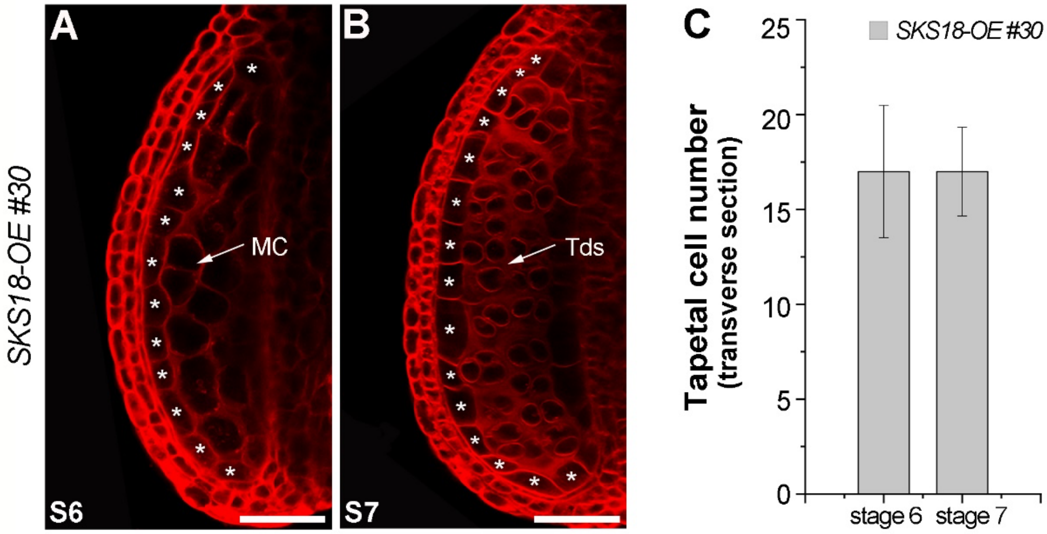

(A-B) One abaxial locule in anther is stained with FM4-64 in *SKS18-OE #30* transgenic plants. Arrowheads indicate the tapetum. Scale bars, 50 µm. (C) Statistic analysis showing tapetal cell number based on the FM4-64 staining in *SKS18-OE #30* from stages 6-7. Data are represented as mean ± SD. n=28 anthers. MC, meiocytes; T, tapetum; Tds.

Although none of the tapetal cells differentiate properly in tdf1, it seems that only a small fraction of them have extra cell divisions. Do the rest of the tapetal cells have normal cell division? If so, why do they have abnormal cell differentiation? It would also be helpful if the authors could comment on why some tapetal cells divide periclinally while others not.

Answer: In this manuscript, we quantified the number of tapetal cells both in WT and *tdf1* at stages 6-8 (Fig. 1I-M). It can be found that only a small fraction of WT tepetal cells undergo anticlinal divisions from stage 6 to stage 7, and they almost no more proliferate from stage 7 to stage 8, supporting that most of them entry into cell differentiation. However, *tdf1* had an obviously increase of tapetal cells (increased 23.9% at stage 6, 50.9% at stage 7 and 56.4% at

stage 8 compared to the WT) shown in Fig.1M. Moreover, the multilayered areas of *tdf1* are quite distinguished (Fig. 1J and 1L). The tapetum development is strictly programmed by many regulators, even one change may severely affect its development and/or function. For instance, precocious or delayed tapetal PCD leads to the pollen abortion (Phan et al., *Plant Cell*, 2011; Xie et al., *Plant Cell*, 2014). Thus, such abnormal proliferations of cells in *tdf1* largely destroy the whole tapetal developmental process, which cannot provide the nutrition and materials to microspores. Evidently, our data also showed that none fatty acid content was synthesized in *tdf1* tapetum beginning at stage 7 (Fig. S8D-F). These results together prove that *tdf1* has the supernumerary tapetal cells which are a serious phenotype and it hinders the normal differentiation in almost all of the tapetal cells. Hoping all of these could provide you a reasonable explanation.

Throughout the manuscript, different indicators such as pollen grain viability, tapetal cell number, and tapetal cell morphology have been used to describe tapetal defects for different plants. It would be necessary to do consistent quantification of the tapetal cell defects for different genotypes, in order to have proper comparisons between plants.

Answer: In this manuscript, we used several genetic materials which including WT, *tdf1*, *sks18*, *SKS18-OE*, *VTC1-OE*, *vtc1 tdf1*, *APX1-OE* and *CAT3-OE*. Among these materials, we tried our best to do consistent quantification just as you mentioned. For example, these materials are all performed by the semi-thin sections for morphology (Figs. 4, 5, 6 and 8).

Actually, the supernumerary tapetal division of *tdf1* was preliminarily discovered by semi-thin sections in our previous study (Zhu et al., *Plant Journal*, 2008). However, to better quantify the over-proliferated tapetal cells, this time we used the FM4-64 dye to stain the tapetum in the most of materials including WT, *tdf1*, *sks18*, *SKS18-OE* and *VTC1-OE* (Figs. 1, 4 and 5). Our results showed that the quantification results based on FM4-64 staining were quite consistent with the morphological observations in those five groups. Therefore, we didn't stain the *vtc1 tdf1* anthers by FM4-64 because their phenotypes shown in semi-thin sections are so reminiscent of those in *SKS18-OE* (Fig. 4P-T and Fig. 6L-N). Additionally, the semi-thin sections from *APX1-OE* and *CAT3-OE* are also similar with those of *sks18* and *VTC1-OE* (Fig. 4F-H, Fig. 5C-E and Fig. 8L-O). We considered these morphological data could still support our conclusions even without FM4-64 staining.

As to the Alexander's staining, it's a common way to quickly distinguish the pollen grains viability. We used this traditional method to stain the materials including WT, *sks18*, *VTC1-OE*, *APX1-OE* and *CAT3-OE*. Although there is still no mature pollen grain formed in *SKS18-OE* and *vtc1 tdf1,* their anthers have a slightly difference to the *tdf1* (Fig. S7B-E). These data also support the restored tapetal phenotype in *tdf1* (Fig. 4P-T and Fig. 6L-N). In this revision, we added these images into the supplementary information. Thanks!

I am also not quite convinced that the tapetum in sks18 or VTC1-OE lines can be called multilayered tapetums, since majority of the tapetal cells are arranged tightly in one layer and only a small part of the locules have extra tapetal cells lied periclinally.

Answer: Thanks for your suggestion! I moderated language in the text and figure legends.

My minor concerns about the manuscript:

In Fig 1, what do the factors and arrowheads show in Q and N?

Answer: The KEULE-GFP has two localizations; one is the cell-plate appearance in interphase cells (Fig. 1N and 1O, arrowheads) and another is the cytoplasmic localization in non-dividing cells (Fig. 1N-O and Fig. S2). According to these observations of KEULE-GFP localization, we quantified the number of tapetal cells showing cell-plate localization versus the total tapetal cells in both WT and *tdf1*. However, this original part of KEULE-GFP including the data presentation and language description were not shown well that confused all the reviewers. Therefore, we re-wrote this part in the text, used the images to indicate the localizations of KEULE-GFP and explained what the arrowheads mean in figure legends (Fig 1. N and O). We also provided a column diagram to indicate that the frequency of cell-plate appearance in *tdf1* was higher than those of WT (Fig. 1P). The relevant statistical data was also provided into the supplemental dataset table. Thanks!

In Fig 2, the data about ams mutant seem not quite related to the major theme of the manuscript, which would be more appropriate to put them in supplementary data.

Answer: I put them into the supplementary information as you suggested (Fig. S4), thanks.

In Fig 2Z and 2B1, for the bars that are are not labeled with *, does it mean that they do not have significant differences from each other?

Answer: In this revision, we labeled other bars as you suggested (Fig. 3). Most of them showed no significant differences, while the low LUC activity was confirmed by the statistics when co-expression the constructs of *Pro35S:AMS* and *ProSKS18:LUC* (Fig. 3C), thanks for your good advice!

In Fig 2A1, there are multiple bands in the EMSA images. Please explain what are the rest.

Answer: The bottom bands in the image are the free probes, which are usually abundant showing the darkest one. We think others are probably resulted from the nonspecific bindings. Because it's hard to completely purify the MBP, we supposed that other proteins may also bind with the probes via other motifs. The Plant Cell has a detailed requirement for the EMSA assay. We followed this requirement and included both mutant probes and unlabeled WT probes to demonstrate sequence-specific binding. Therefore, we believed that this EMSA assay could support our conclusions, thanks.

What are the red fluorescent signals in Fig 7D to 7K?

Answer: These are the autofluorescences from the chloroplasts. I added this annotation into the figure legends, thanks!

What do Fig 7D to 7K tell us? Do they here show the expression pattern of the pDYT1550 promoter? They actually show that this tapetum-specific promoter is also active in other anther wall cell layers.

Answer: The Fig. 7D-7K tell that these genes are activated in the tapetum by the pDYT1550 promoter. As you say, some fluorescence signals also appear in the outer anther layers. It has been showed that DYT1-EYFP driven by its native promoter is expressed faintly in anther lobe including those outer anther layers (Cui et al., *Plant Cell*, 2016). Therefore, we think that pDYT1550 promoter can also activate genes express in the epidermis and endothecium. I revised the description in the text and figure legends. Hoping these data could provide you a reasonable explanation.

More attention should be paid to the grammar in this manuscript. There are lots of mistakes in the use of tenses and plurals. For example, "ROS" is a plural.

Answer: Yes, it is. In this revision, we carefully revised our language including the whole text and figure legends, thanks for your suggestions!

TPC2022-RA-00796R1 2nd Editorial decision – *revision requested* November 24, 2022

We have received reviews of your manuscript entitled "Ascorbic acid-mediated ROS homeostasis modulates the switch from tapetal cell division to cell differentiation in Arabidopsis." Thank you for submitting your best work to The Plant Cell. The editorial board agrees that the work you describe is substantive, falls within the scope of the journal, and may become acceptable for publication, pending revision and potential re-review.

As you will read below, Reviewer #3 has only minor comments that are easily addressed. However, Reviewer #1 still has some remaining concerns about the ROS measurements. I discussed this with Reviewer 1, who reminded me that they were skeptical of ROS level measurements in the previous version. The reduction of ROS levels in the tdf1 mutant is clear, yet that in sks18 mutant is subtle and difficult to observe in fluorescent images (Fig. 7G-I).

In the revision, you added the measurement of O2- level, but the reviewer felt that this is not a direct answer to their question. Instead of methodological improvements, you described that the signal intensity was calculated by average of four different points in same sample. As the reviewer mentions, ROS staining is unstable, displaying patchy signal and variation across anthers. The data in Fig. 8 showing that OE of ROS scavengers leads to multilayered tapetum are indirect evidence of ROS involvement in tapetum development.

In addition, the data behind the box plot in Figure 7M are not shown, and a single image of ROS staining image is insufficient and inconclusive.

I ask that in a revision, you address this directly. This would require more robust evidence that could include the

inclusion of multiple images used for the statistics in Fig. 7M boxplots, with the selected regions in each photo indicated (this could go in a supplemental figure).

I would like for you to directly address this concern of the reviewer, in a way that satisfies the reviewer. If you cannot provide better supporting data for the role of ROS, you would need to substantially weaken those parts of your claims - removing the ROS quantification and modifying the text and title.

Please contact us if there are ambiguous comments or if you wish to discuss the revision.

TPC2022-RA-00796R2 2nd Revision received January 7, 2023

Reviewer comments and **author responses**:

Reviewer #1:

To answer my previous questions, the authors added several results, such as Fig. S13 and Fig. 1P. However, the revisions this time didn't convince me, especially in SKS18 function in AsA-mediated control of ROS levels in tapetum.

The authors answered to my previous question as follows; "In our ROS staining experiment, we took the images with a relative consistent staining degree to exclude the staining interference. Even so, sometimes the tapetal cells in one image have different fluorescence intensities. Then, four different areas with different ROS intensities in a single tapetum were measured, and finally the means were calculated from about 10 independent pictures. All of these could reduce the error and improve our results quality".

I understand the results of ROS staining are often unstable. However, I do NOT think that the calculation of ROS intensity by "measurements of median (or average) of four different areas in a single tapetum" is a correct way, because the median/average value may easily fluctuate depending on which four areas the authors selected. The box plots in Fig. 7M display the range of median values of 10 biological replicates, but not reflect the statistical dispersion of fluctuation among four areas. Thus, I cannot validate the quantification of ROS difference between WT and sks18 at all in Fig. 6. The result in Fig. S13, which was newly added in the revised manuscript, is as well.

The overexpression of ROS scavengers, APX1-OE and CAT3-OE, resulted in multilayered tapetum, like as in tdf1, sks18 and VTC1-OE plants, and considering together with the above results, the authors concluded that "the appropriate AsA-mediated ROS scavenging constrains cell divisions in tapetum (L470-472)". Yes, I agree with the idea that ROS level is somehow important for tapetum development. However, it is an indirect evidence to connect the AsA function to ROS-level control in tapetum.

Conclusively, I agree with the idea that TDF1 promotes SKS18 and represses VTC1 at protein levels, and that ROS is important for tapetum development, as figured in Fig. 9 model. However, from the above reasons, I don't think the idea that AsA mediates ROS levels in tapetum development, and the title "Ascorbic acid-mediated ROS homeostasis modulates the switch from tapetal cell division to cell differentiation in Arabidopsis", are fully supported by the results submitted.

Answer: Thanks for you patiently reviewing our manuscript again. First, we are appreciated these precious comments! Considering your opinions, we did take the ROS intensity quantification very seriously! We wondered whether there are other better ways for ROS quantification, which might be able to bypass the instability of ROS staining. We then discussed this issue with Prof. Chao Li who established that pollination in WT Arabidopsis triggers a significant reduction of ROS levels in stigmatic papilla cells (Liu et al., *Science*, 2021). Unfortunately, that quantifying the ROS intensity based on the images from ROS staining seems a general and well-acknowledged method at present. Therefore, instead of methodological improvements, we thought about our ROS measurement again. Just as you said, no matter how many partial regions we selected, there are the biases resulting from this measurement. So, we selected the whole tapetal cells from one abaxial locule in each anther and calculated their means this time (the images were shown in the Supplemental data 3). This adjustment could avoid the disadvantage from the subjective choose as you mentioned.

During this measurement, we tried our best to use those images with homogeneous staining and similar staining intensities. Considering your doubt of the ROS levels between WT and *sks18*, we provided all the WT and *sks18* fluorescent images used for the statistics in Fig. 7M box plots (Supplemental data 3). There are 58 images at all. I labelled the tapetum with white line and attached with its mean in each image. I also made six figures which combined each image for better comparison between WT and *sks18* from stages 6-8. As you can see, when the ROS intensities in microsporocytes were similar or even stronger in *sks18* than that in WT, the tapetal ROS intensities of WT were higher than that of *sks18*. For example, the tapetal ROS intensity of WT (mean = 18.613) was higher than that of *sks18* (12.821), but their intensities in microsporocytes were opposite. The similar case like the tapetal ROS intensity in WT (mean = 11.587) vs in *sks18* (mean = 9.919). At stage 7, the tetrad ROS signals of WT were generally stronger than those of *sks18*. Given that SKS18 protein surrounds the tetrads (Fig. 2N), we cannot exclude the possibility that the ROS accumulation in *sks18* tetrads might be also affected. Thus, although sometimes the epidermis and endothecium were separated or incomplete, we still compared the groups of WT and *sks18* under the similar ROS signals of epidermis and endothecium as possible. For example, the tapetal ROS intensity of WT (mean = 23.502) was higher than that of *sks18* (mean = 15.138). You can also find this case in the groups between WT (mean = 24.742) and *sks18* (mean = 14.882). Consistently, the tapetal ROS signals of WT were higher than those of *sks18* at stage 8, such as the tapetal ROS intensity of WT (mean = 17.636) vs *sks18* (mean = 12.52); WT (mean = 10.528) vs *sks18* (mean = 7.436), but their ROS signals of epidermis and endothecium had no obvious difference. Finally, the statistical analysis showed there is still a difference of tapetal ROS levels between WT and *sks18* (Fig. 7M). We also re-measured the tapetal ROS levels among anthers from *tdf1* and *VTC1-OE*, as a result, their tapetal ROS tendencies did not change (Fig. 7M). However, two places were changed according to the new measurement: 1) the value from the previous Fig. 7C (WT at stage 8) was abnormal, so we replaced a new image with normal value. 2) There was no significant difference of $O_2^{\cdot-}$ levels between WT and *tdf1* at stage 8 (Fig. S13G). In this revision, the box plots were also added with the individual data points (Figs. 7M, S13G and S14D). Hoping all these adjustments and data could gain your recognition!

I understood that since you were skeptical of SKS18 function in AsA-mediated ROS levels, so overexpression of ROS scavengers may not enough to show the extra tapetal cells resulted from the reduction of ROS levels is connected with AsA function. Therefore, we provided the results from the *ProDYT1550:SKS18-GFP* overexpression lines (*SKS18-OE*) in this revision. We had confirmed that the expression of *SKS18* was increased in *SKS18-OE #30* line (Fig. S14A) and showed that overexpression of *SKS18* decreased the number of tapetal cells compared with those of WT (Fig. S14, E and F). Then, a low level of AsA in *SKS18-OE #30* inflorescences was detected (Fig. S14B). Next, we measured the tapetal ROS intensities in *SKS18-OE #30* anthers. We found that the tapetal ROS levels in *SKS18-OE #30* were higher than that in WT at stage 7 (Fig. S14D). I think this is a direct evidence to connect AsA function to ROS-levels control in tapetum. Additionally, we noticed that the ROS intensities in some *SKS18-OE #30* tetrads were extremely high (Fig. S14C), which was opposite to the ROS accumulating pattern in the *sks18* tetrads. These phenotypes supported our assumption that SKS18 might also modulate the ROS levels in the tetrads and explain why the ROS intensities of *sks18* tetrads were weaker than that of WT. I provided these ROS images of *SKS18-OE* in the Supplemental data 3, too.

In conclusion, these results together showed that the reduction of AsA accumulation resulted from SKS18 overexpression increases ROS levels, which inhibits the tapetal cell divisions (Fig. S14). Combining our previous VTC1-OE data that the over-accumulated AsA reduces ROS levels which induces the extra tapetal cells (Figs. 5B-J, 7J-M), we believed all these results could support our claims that AsA-mediated ROS homeostasis modulates the switch from tapetal cell division to cell differentiation in Arabidopsis. Last but not the least, thanks for your all comments to improve the quality of our manuscript!

Reviewer #3:

The authors made significant efforts to answer the previous comments. I do not have major comments this time. However, there are many grammatical mistakes in the text which I think would disrupt the flow of the manuscript. Below are some examples.

Thanks for your comments to improve the quality of our manuscript!

In the abstract, "either knockout of SKS18 or VTC1 overexpression raises AsA concentrations, resulting in multilayered tapetum" should be revised, as has been moderated in the text.

Answer: I revised it in the abstract, thanks.

L323: what do the stained purple remnants suggest?

Answer: In the Alexander's staining solution, malachite green is used for staining pollen wall and acid fuchsin for staining cytoplasm and mitochondria. In the typical stain, aborted pollen grains without cytoplasm are green while nonaborted pollen grains are red or purple (Alexander, 1969). Thus, some remnants stained purple in the *tdf1 SKS18-OE* line suggests that the cytoplasm of those aborted pollen are not totally degenerated, although the mature pollen grains still could not be produced. I revised this sentence and added the reference, thanks.

TPC2022-RA-00796R2 3rd Editorial decision – *accept with minor revision* January 11, 2023

We have received a quick review of your manuscript entitled "Ascorbic acid-mediated ROS homeostasis modulates the switch from tapetal cell division to cell differentiation in Arabidopsis." On the basis of the advice received, the board of reviewing editors would like to accept your manuscript for publication in The Plant Cell. This acceptance is contingent on revision based on the comments of our reviewers. In particular, please consider the comment from Reviewer #1 about adding some text to the methods section about how you performed the ROS staining and quantification. That reviewer (and I) was quite appreciative of the effort to which you went to address their concern - so thank you for that!

TPC2022-RA-00796R3 3rd Revision received January 14, 2023

Reviewer comments and **author responses**:

Reviewer #1:

commend the authors for taking my comments seriously and performing many additional experiments in this version. Fig. S13 confirms the quantification of superoxide levels, and certainly shows that the ROS level is significantly increased in the tdf mutant. The authors also showed that SKS18-OE inhibits tapetal cell division. These additional experiments succeeded to provide further support for the hypothesis presented in Fig. 9.

As a minor comment, I could find no description about ROS staining and quantification in Methods section. .

RESPONSE: Answer: I added these descriptions as a separated paragraph in Methods section, thank you!

TPC2022-RA-00796R3 4th Editorial decision – *acceptance pending* January 18, 2023

Major comments (include only the customized paragraphs from decision letter).

Final acceptance from Science Editor Feb. 3, 2023
